# Microglial targeted therapy relieves cognitive impairment caused by *Cntnap4* deficiency

**DOI:** 10.1002/EXP.20220160

**Published:** 2023-05-10

**Authors:** Wenlong Zhang, Huaqing Chen, Liuyan Ding, Jie Huang, Mengran Zhang, Yan Liu, Runfang Ma, Shaohui Zheng, Junwei Gong, Juan C. Piña‐Crespo, Yunlong Zhang

**Affiliations:** ^1^ Department of Neurology The First Affiliated Hospital of Guangzhou Medical University Guangzhou China; ^2^ Key Laboratory of Neurological Function and Health School of Basic Medical Sciences Guangzhou Medical University Guangzhou China; ^3^ School of Life Sciences Westlake University Hangzhou China; ^4^ Westlake Laboratory of Life Sciences and Biomedicine Hangzhou China; ^5^ Shenzhen Key Laboratory of Gene and Antibody Therapy Center for Biotechnology and Biomedicine State Key Laboratory of Chemical Oncogenomics State Key Laboratory of Health Sciences and Technology Institute of Biopharmaceutical and Health Engineering Shenzhen International Graduate School Tsinghua University Shenzhen China; ^6^ School of Traditional Chinese Medicine Jinan University Guangzhou China; ^7^ Degenerative Diseases Program Center for Genetic Disorders and Aging Research Sanford Burnham Prebys Medical Discovery Institute La Jolla California USA

**Keywords:** *Cntnap4*, memory processing, microglial targeted delivery, PLX3397, pro‐inflammatory response

## Abstract

Contactin‐associated protein‐like 4 (Cntnap4) is critical for GABAergic transmission in the brain. Impaired Cntnap4 function is implicated in neurological disorders, such as autism; however, the role of Cntnap4 on memory processing is poorly understood. Here, we demonstrate that hippocampal *Cntnap4* deficiency in female mice manifests as impaired cognitive function and synaptic plasticity. The underlying mechanisms may involve effects on the pro‐inflammatory response resulting in dysfunctional GABAergic transmission and activated tryptophan metabolism. To efficiently and accurately inhibit the pro‐inflammatory reaction, we established a biomimetic microglial nanoparticle strategy to deliver FDA‐approved PLX3397 (termed MNPs@PLX). We show MNPs@PLX successfully penetrates the blood brain barrier and facilitates microglial‐targeted delivery of PLX3397. Furthermore, MNPs@PLX attenuates cognitive decline, dysfunctional synaptic plasticity, and pro‐inflammatory response in female heterozygous *Cntnap4* knockout mice. Together, our findings show loss of *Cntnap4* causes pro‐inflammatory cognitive decline that is effectively prevented by supplementation with microglia‐specific inhibitors; thus validating the targeting of microglial function as a therapeutic intervention in neurocognitive disorders.

## Graphical abstract

Hippocampal *Cntnap4* deficiency was associated with cognitive impairment via inducing pro‐inflammatory response in females. A biomimetic microglial nanoparticle system (MNPs@PLX) was established for precise microglial delivery of FDA‐approved PLX3397 to attenuate cognitive decline, dysfunctional synaptic plasticity, and pro‐inflammatory response in female heterozygous *Cntnap4* knockout mice. This study indicates that microglial supplementation of PLX3397 may serve as an intervention for cognitive impairment.

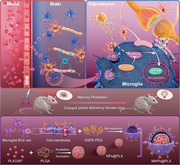

## 1 INTRODUCTION

Contactin‐associated protein‐like 4 (Cntnap4, also known as Caspr4) is a transmembrane protein member of the neurexin superfamily, which is critical for neurological development and synaptic function.^[^ ^1^, ^2^ ^]^ In the mouse brain, Cntnap4 is expressed in developing interneurons in the olfactory bulb, hippocampus, and deep cerebellar nuclei; and in dopaminergic (DA) neurons in the substantia nigra.^[^ ^2^ ^]^ Like neurexins, Cntnap4 contains a carboxy‐terminal binding site for postsynaptic density protein of 95 kilodaltons (PSD‐95), disc large, zona occludens (PDZ) domains.^[^ ^2^ ^]^ Through its PDZ‐containing domain, Cntnap4 interacts with Mint1, calcium/calmodulin‐dependent serine protein kinase, and Ligand Numb‐protein X2 to regulate GABAergic (γ‐aminobutyric acid‐producing) transmission and neuronal differentiation.^[^ ^3^ ^]^ As verification of its important role in neurological development and GABAergic transmission, the *CNTNAP4* gene has recently been identified as a novel susceptibility determinant for autism spectrum disorders (ASDs), childhood‐onset schizophrenia, and epilepsy.^[^ ^4^, ^5^, ^6^ ^]^ Intriguingly, Cntnap4 is also associated with neurodegenerative diseases, and one of *CNTNAP4* intronic copy number variation (CNV) polymorphisms (R.6782.1del/del variant) has been reported to be associated with aging‐related diseases in females, including cognitive impairment, late onset Alzheimer's disease (LOAD), and Parkinson's disease (PD).^[^ ^7^ ^]^ Our recent work revealed that *Cntnap4* deficiency in DA neurons results in pathology associated with PD.^[^ ^8^ ^]^ In the hippocampus, Cntnap4 is predominantly expressed in parvalbumin (PV)‐positive GABAergic basket cells of the dentate gyrus and pyramidal cell layers CA1‐CA3.^[^ ^2^ ^]^ However, whether Cntnap4 regulates hippocampal cognitive function remains undetermined.

Microglia play an important role in regulating cognitive function. Under normal conditions, microglia prune synapses to modulate neuronal activity, synaptic plasticity as well as learning and memory via C1q, C3, and CR3 complement signaling or the CD47 signaling pathway.^[^ ^9^ ^]^ Whilst overactivation of microglia damages synaptic transmission and memory function via releasing pro‐inflammatory cytokines and inducing inflammation.^[^ ^10^ ^]^ Interestingly, microglia–interneuron communication regulates neural circuits and microglial function which are related to learning and memory.^[^ ^11^ ^]^ Besides, GABA was reported to suppress inflammatory response.^[^ ^12^ ^]^ Since PV‐expressed Cntnap4 was indicated to regulate GABAergic transmission, we ask whether Cntnap4 influences inflammatory reaction in cognitive impairment via GABA.

**SCHEME 1:**
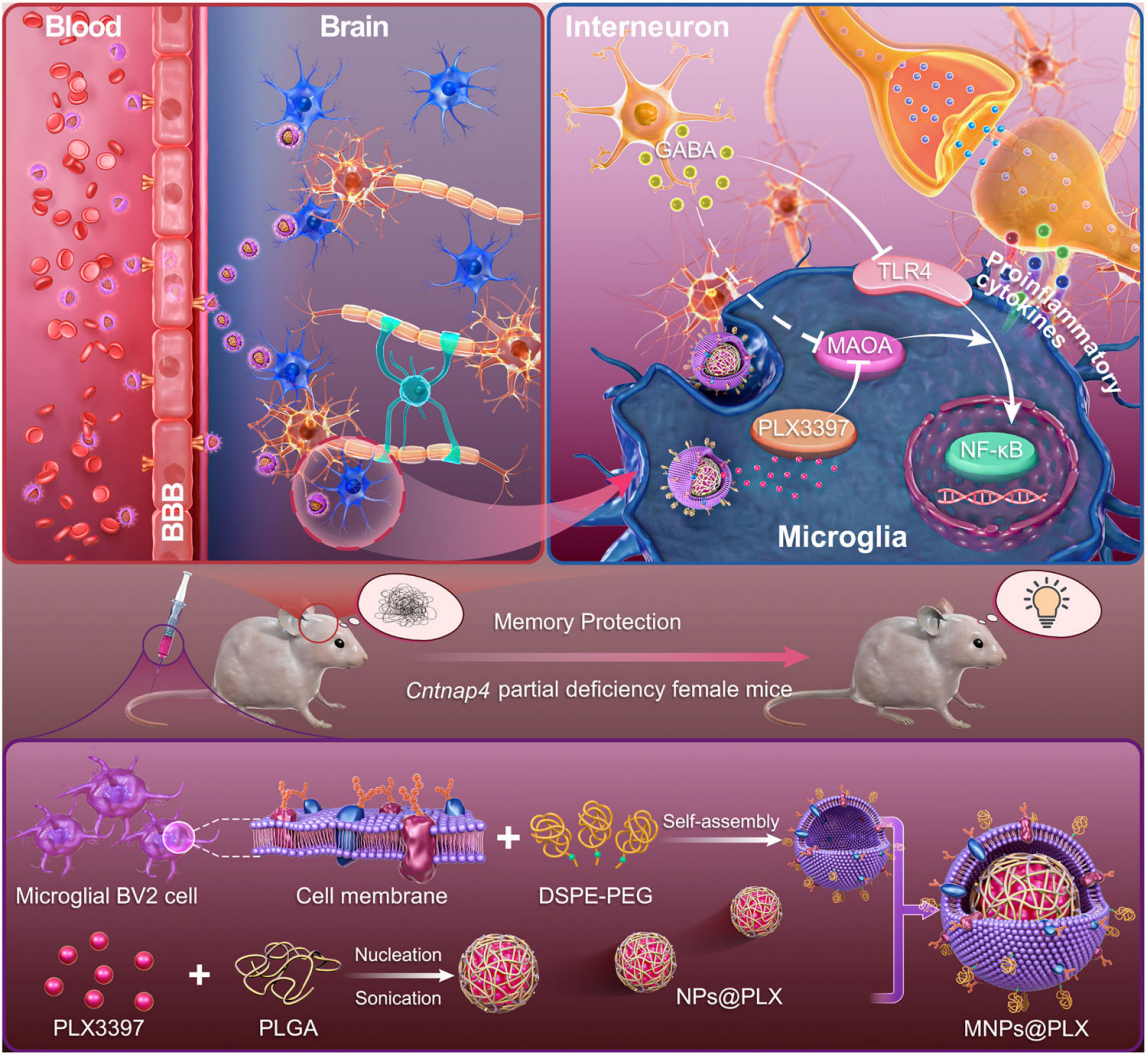
Schematic illustration showing the underlying mechanism of *Cntnap4* deficiency‐induced memory loss and the therapeutic process of MNPs@PLX. (Top row) Under normal conditions, interneurons release GABA to protect memory via inhibition of the pro‐inflammatory TLR4/NF‐κB signaling pathway, which is activated by MAOA. In Cntnap4^+/−^ mice, which display dysfunctional GABAergic transmission, increased MAOA activity, and increased TLR4/NF‐κB signaling, MNPs@PLX penetrates the blood‐brain barrier (BBB) by binding cell surface receptors on the brain endothelial cells and accumulates in microglia. The released PLX3397 then inhibits the TLR4/NF‐κB signaling pathway by inactivating MAOA. (Middle row) MNPs@PLX treatment rescues the impaired cognitive function caused by *Cntnap4* deficiency by suppressing the inflammatory response. (Bottom row) The biomimetic nanocarriers (MNPs@PLX) are prepared by extracting microglial BV2 cell membranes using ultracentrifugation; preparing PLX3397‐coated PLGA nanoparticles as the core; and coating the isolated microglial BV2 cell membranes on the surface of the nanoparticles by extrusion. DSPE, 1,2‐distearoyl‐sn‐glycero‐3‐phosphoethanolamine‐*N*‐folatec; PEG, poly (ethylene glycol).

In this study, for the first time, we explored the function of Cntnap4 in hippocampal memory processing. We report that hippocampal *Cntnap4* deficiency in female mice, including mice injected with adeno‐associated virus (AAV)‐packaged Cntnap4 shRNA and heterozygous *Cntnap4* knockout (Cntnap4^+/−^) mice, manifests as cognitive impairment and dysfunctional synaptic transmission. The underlying mechanisms may involve pro‐inflammatory response resulting from impaired GABAergic transmission, given that GABA supplementation rescues the cognitive decline and dysfunctional synaptic transmission in female Cntnap4^+/−^ mice. To modulate *Cntnap4* deficiency‐induced inflammatory response, we employed PLX3397, a FDA‐approved colony stimulation factor 1 receptor (CSF1R) inhibitor.^[^ ^13^, ^14^ ^]^ PLX3397 is usually given in chow to deplete microglia in the brain, however, its low efficiency at crossing the blood‐brain barrier (BBB) hinders its application.^[^ ^13^, ^14^ ^]^ To efficiently and accurately inhibit microglial activation, we established a biomimetic microglial nanoparticle (BMNPs) strategy to deliver PLX3397. The BMNPs platform (termed MNPs@PLX) was designed with an encapsulated PLX3397‐coated poly lactic‐*co*‐glycolic acid (PLGA) nanoparticles core and a microglial BV2 cell membrane shell (Scheme 1). Our results reveal that MNPs@PLX can successfully penetrate the BBB and facilitate microglial targeted delivery of PLX3397 (Scheme 1). Furthermore, MNPs@PLX attenuated *Cntnap4* loss‐induced cognitive decline, dysfunctional synaptic plasticity, and pro‐inflammatory response (Scheme 1). These effects may result from MNPs@PLX‐mediated inhibition of monoamine oxidase A (MAOA) activation (Scheme 1). Taken together, our results suggest that *Cntnap4* loss is a cause of cognitive decline that is associated with inflammatory response, for which microglial supplementation of PLX3397 may serve as an intervention.

## 2 RESULTS

### 2.1 Hippocampal *Cntnap4* knockdown impairs cognitive function and synaptic plasticity

**FIGURE 1:**
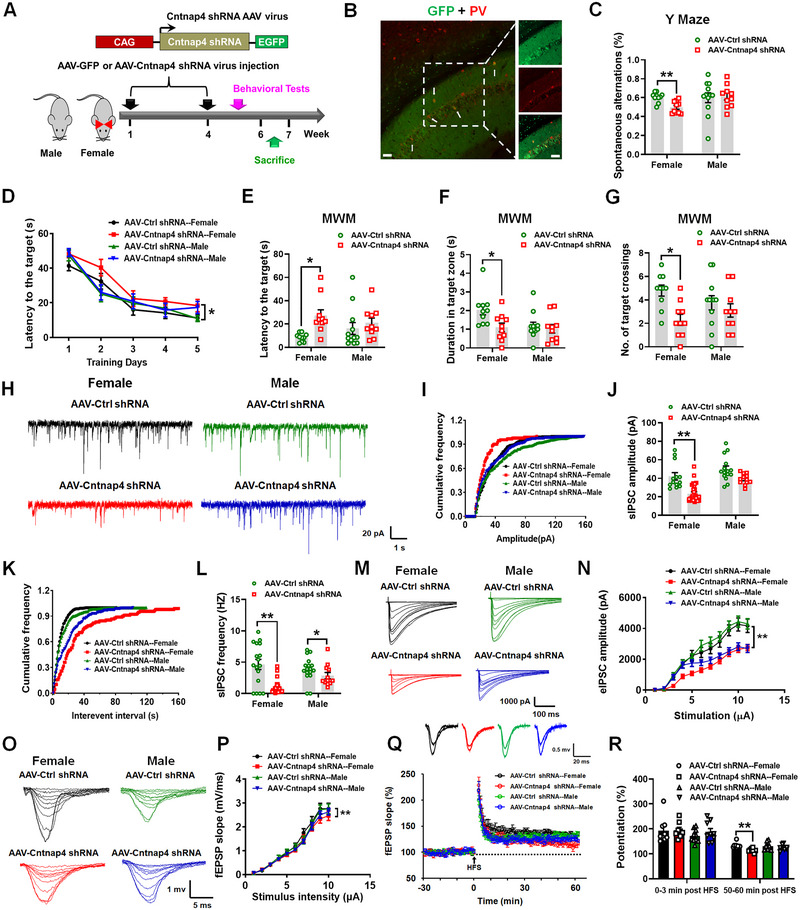
*Cntnap4* knockdown impairs cognitive function and synaptic plasticity in female mice. A) Experimental design for *Cntnap4* knockdown in male and female mice. B) Immunofluorescence staining of GFP with PV in the hippocampus. The white arrows indicate the co‐localization of GFP+ and PV+ cells. Scale bar in left panel is 50 µm, and in right panel is 25 µm. C) Y maze results for mice with *Cntnap4* knockdown in the hippocampus are presented as spontaneous alternations (%). D) The escape latency over a 5‐day training course. In the probe test, mice were analyzed for E) escape latency, F) time spent in the target zone, and G) number of target crossing. *n* = 10 in the adeno‐associated virus (AAV)‐Ctrl shRNA—female, AAV‐Cntnap4 shRNA—female, and AAV‐Cntnap4 shRNA—male groups, *n* = 12 in the AAV‐Ctrl shRNA—male group. H) Representative traces of GABA receptor‐mediated spontaneous inhibitory postsynaptic currents (sIPSC). All sIPSCs were recorded at a holding potential of −70 mV. I,J) Cumulative frequency plots of the sIPSC amplitude and quantitative analysis of the amplitude of GABA receptor‐mediated sIPSCs. *n* = 12−29. K,L) Cumulative frequency plots of the inter‐event interval and quantitative analysis of the frequency of GABA receptor‐mediated sIPSCs. *n* = 12−29. M,N) Representative traces of evoked inhibitory postsynaptic currents (eIPSCs) stimulus intensities and stimulus‐response curves of hippocampal pyramidal neurons from the indicated treatment groups. *n* = 12−22. O) Representative traces of input‐output stimulus intensities. P) Input–output relations generated by stimulating SCs and recording in CA1 stratum radiatum. *n* = 8−10. Q) Effects of hippocampal *Cntnap4* deficiency on the long‐term synaptic potentiation (LTP) at the SC‐CA1 synapses. The images show representative traces of field excitatory postsynaptic potentials (fEPSP) recordings of responses before and 50 min after high‐frequency stimulation (HFS; arrow). R) Quantitative analysis of data in (Q). The level of fEPSP potentiation was determined as a mean of the levels at 0−3 min and 50−60 min after high‐frequency stimulation. *n* = 8−10. Results are expressed as the mean ± SEM. ^**^ *p* < 0.01, ^*^ *p* < 0.05 versus AAV‐Ctrl shRNA group. Statistical significance was determined by two‐way ANOVA and Bonferroni tests for post hoc comparisons.

To investigate the role of Cntnap4 in sex‐specific cognitive function, we delivered a *Cntnap4* AAV‐shRNA virus that targets a previously identified sequence into the hippocampi of male and female mice.^[^ ^8^ ^]^ Four weeks later, behavioral tests were performed (Figure 1A). The knockdown efficiency of AAV‐Cntnap4 shRNA in the hippocampus was confirmed (Figure S1A,B, Supporting Information), and the AAV was also confirmed to mainly label PV+ cells but not Iba1 or GFAP+ cells (Figure 1B, Figure S1C, Supporting Information). In an assessment of locomotor activity, *Cntnap4* knockdown mice showed no obvious changes in the time spent in the center and the number of entries to the center in the open field (Figure S2A,B, Supporting Information); however, the total traveled distance and movement speed were increased in female and male mice (Figure S2C,D, Supporting Information). Notably, *Cntnap4* knockdown in female but not male mice resulted in worse performance on the Y maze alternation test of working memory, with impaired learning response and increased escape latency over a 5‐day training phase in female knockdowns as compared with controls (Figure 1C,D). Furthermore, in the Morris water maze test of spatial memory, female *Cntnap4* knockdown mice took longer to reach the target, spent less time in the target zone, and underwent fewer target crossings, as compared with female control mice (Figure 1E–G). Moreover, *Cntnap4* knockdown in females but not males increased the total traveled distance, movement speed, and total arm entries in the Y maze (Figure S3A–C, Supporting Information), though no significant differences were observed in total distance and swimming speed in the 5‐day training and probe trial tests (Figure S3D–G, Supporting Information).

As Cntnap4 modulates GABAergic transmission, which is critical for the hippocampal memory processing,^[^ ^15^ ^]^ we evaluated the effects of *Cntnap4* knockdown on hippocampal spontaneous inhibitory postsynaptic currents (sIPSC). The results show that both the amplitude and frequency were significantly decreased in female *Cntnap4* knockdown mice, as compared with female controls (Figure 1H–L). The frequency of sIPSC was also decreased in male *Cntnap4* knockdown mice, as compared with male controls (Figure 1K,L). Consistently, the amplitude of evoked inhibitory postsynaptic currents (eIPSCs) was lower in hippocampal pyramidal neurons from both female and male *Cntnap4* knockdown mice (Figure 1 M,N). Furthermore, the input‐output response of basal transmission in CA1 was significantly impaired in female but not male *Cntnap4* knockdown mice (Figure 1O,P). Additionally, the post‐tetanic potentiation and long‐term synaptic potentiation (LTP) at Schaffer collateral pathway (SC‐CA1) synapses were reduced in hippocampal slices, and the LTP amplitude at 50−60 min after LTP induction was significantly decreased in *Cntnap4* knockdown female but not male mice (Figure 1Q,R). Besides, there was no difference in action potentials (APs) between both female and male mice injected with Ctrl shRNA and Cntnap4 shRNA (Figure S4, Supporting Information). These data suggest that *Cntnap4* knockdown impairs cognitive performance, hippocampal inhibitory synaptic transmission, and synaptic plasticity.

### 2.2 Hippocampal *Cntnap4* deficiency in female mice induces a pro‐inflammatory reaction

**FIGURE 2:**
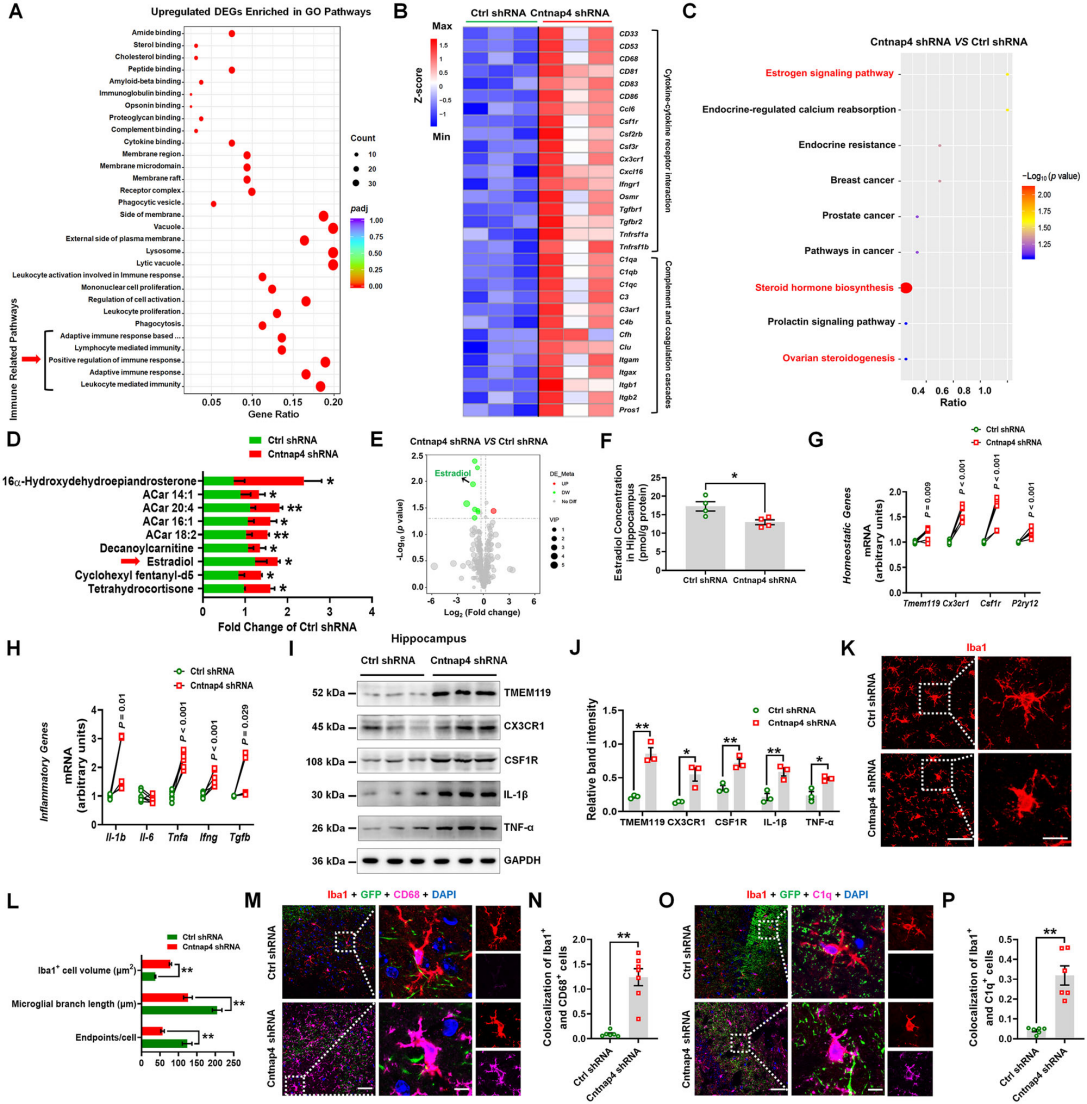
*Cntnap4* knockdown in female mice induces pro‐inflammatory response. A) GO pathways enriched by upregulated differential expressed genes (DEGs) in hippocampi of adeno‐associated virus (AAV)‐Cntnap4 shRNA versus AAV‐Ctrl shRNA groups. B) Hierarchical clustering of DEGs enriched in the immune related pathways between the AAV‐Ctrl shRNA and AAV‐Cntnap4 shRNA groups. C–E) Differential metabolites identified by metabolomics analysis and the enriched pathways between the AAV‐Ctrl shRNA and AAV‐Cntnap4 shRNA groups. F) Estradiol levels in the hippocampus were evaluated by ELISA. G,H) The mRNA expression levels of *Tmem119*, *Cx3cr1*, *Csf1r*, *P2ry12*, *Il‐1b*, *Il‐6*, *Tnfa*, *Ifng*, and *Tgfb* in the hippocampus between the AAV‐Ctrl shRNA and AAV‐Cntnap4 shRNA groups. *n* = 6 per group. I,J) Representative blots and quantification showing TMEM119, CX3CR1, CSF1R, IL‐1β and TNF‐α expression in hippocampi of AAV‐Ctrl shRNA and AAV‐Cntnap4 shRNA groups. *n* = 3 per group. K) Immunofluorescence staining of Iba1‐positive cells in hippocampi of mice injected with AAV‐Ctrl shRNA or AAV‐Cntnap4 shRNA. Scale bars, 40 µm. Magnified images are shown in the right column. Scale bars, 10 µm. L) Quantification of endpoint voxels, branch length, and volume of Iba1‐positive cells. *n* = 8 per group. M–P) Immunofluorescence staining and quantification of Iba1 colocalization with M) CD68 or O) C1q in hippocampi of AAV‐Ctrl shRNA and AAV‐Cntnap4 shRNA groups. *n* = 6−7. Scale bars, 40 µm. Magnified images are shown in the right columns. Scale bars, 8 µm. Results are expressed as the mean ± SEM. ^**^ *p* < 0.01, ^*^ *p* < 0.05 versus AAV‐Ctrl shRNA. Statistical significance was determined using the Student's *t*‐test.

To explore the mechanisms underlying hippocampal *Cntnap4* knockdown‐induced cognitive decline, we performed RNA‐seq. The volcano map of differential expressed genes (DEGs), Venn diagram, and PCA plot between Ctrl shRNA and Cntnap4 shRNA groups are shown (Figure S5A–C, Supporting Information). Upregulated DEGs were enriched in immune related pathways, such as “Positive regulation of immune response” in GO pathways (Figure 2A, Figure S5D, Supporting Information), and “Cytokine‐cytokine receptor interaction,” “Apoptosis” and “Complement and coagulation cascades” in Kyoto encyclopedia of genes and genomes (KEGG) pathways (Figure S5E,F, Supporting Information). Notably, upregulated DEGs were associated with microglial activation (*CD33*, *CD68*, *CD81*, *CD83*, *CD86*, *Ccl6*, *Csf1r*, *Cx3cr1*, *Cxcl16*, *Tnfrsf1a*) (Figure 2B). On the other hand, downregulated DEGs were enriched in the “Memory,” “Long‐term memory,” and “Steroid metabolic process” GO pathways and “Cortisol synthesis and secretion,” “Ovarian steroidogenesis,” and “Steroid biosynthesis” KEGG pathways (Figure S5G,H, Supporting Information). Because cognitive decline is found in females and downregulated DEGs were linked with sterol metabolic and biosynthesis process, we next examined differential metabolites between Ctrl shRNA and Cntnap4 shRNA by metabolomic analysis. Intriguingly, downregulated hippocampal metabolites in the positive ion mode, especially including estradiol, were enriched in the “Estrogen signaling,” “Steroid hormone biosynthesis,” and “Ovarian steroidogenesis” KEGG pathways (Figure 2C–F). Furthermore, upregulated metabolites in the negative ion mode included prostaglandin E2 (PGE2) and 6α‐prostaglandin I1, while hippocampal metabolites were enriched in the “inflammatory mediator regulation of TRP channels” pathway (Figure S6A–C, Supporting Information). Notably, *Cntnap4* knockdown significantly increased both homeostatic genes (*Tmem119*, *Cx3cr1*, *Csf1r*, and *P2ry12*) and inflammatory genes (*Il‐1b*, *Tnfa*, *Ifng*, and *Tgfb*), as well as the protein expressions of TMEM119, CX3CR1, CSF1R, IL‐1β and TNF‐α (Figure 2G–J). Consistently, *Cntnap4* knockdown increased the volume of Iba1+ cells, while reducing the process complexity and endpoint voxels (Figure 2K,L), suggesting that *Cntnap4* knockdown promotes a change in the microglia from a “resting” phenotype to an “activated/immune‐responsive” phenotype. Co‐staining of Iba1 with CD68 and C1q, two classic microglial activation markers, verified that *Cntnap4* knockdown activates microglia in the hippocampus (Figure 2 M–P). We also examined the effect of estrogen administration on the pro‐inflammatory response, and we found estrogen suppressed microglia activation in the hippocampus of mice injected with AAV‐Cntnap4 shRNA (Figure S7, Supporting Information). Collectively, these data show that *Cntnap4* knockdown in female induces an immune response that may be associated with reduced estradiol levels.

### 2.3 Female heterozygous *Cntnap4* knockout mice manifest cognitive decline and activated immune response

**FIGURE 3:**
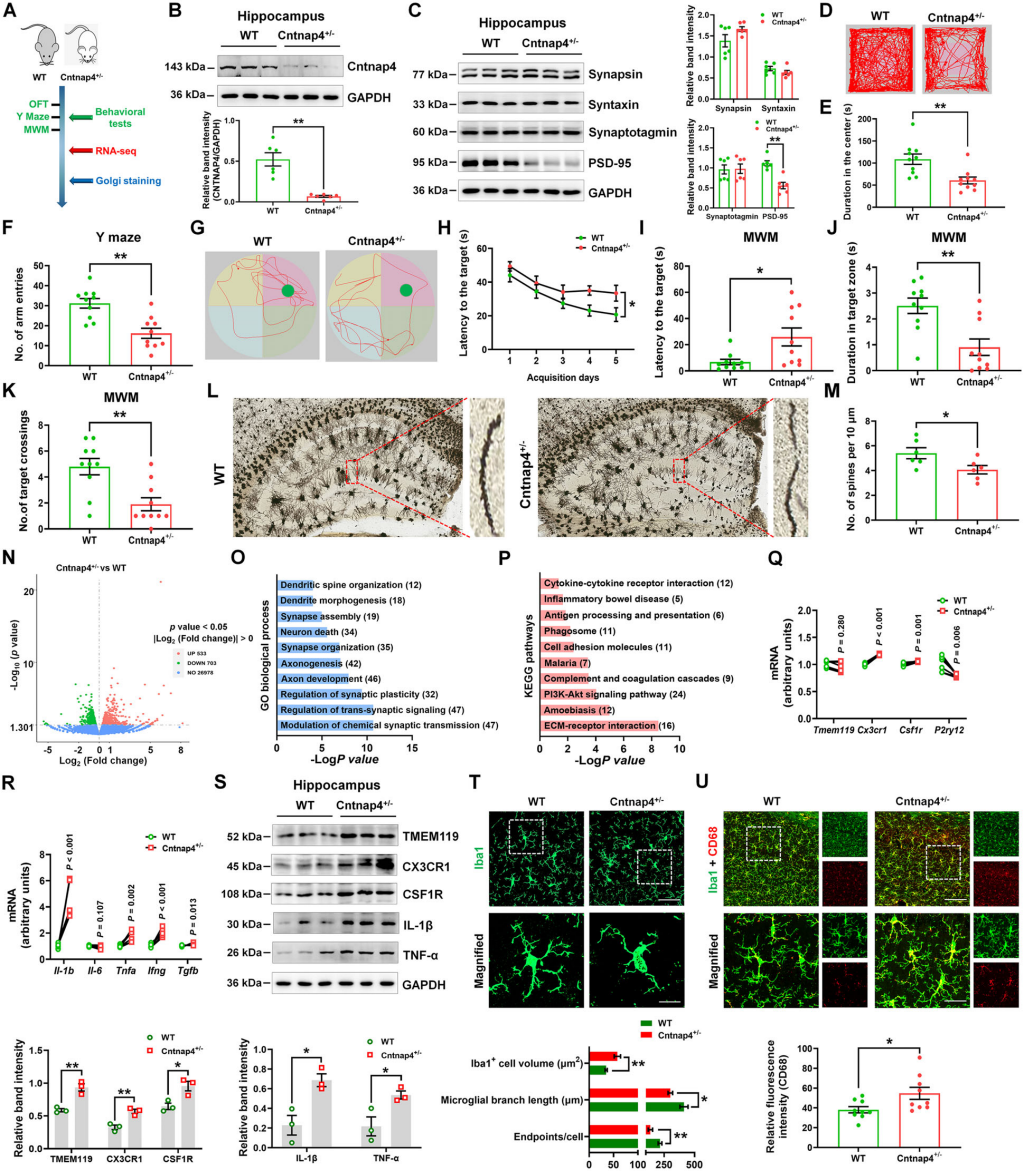
Female Cntnap4^+/−^ mice exhibit cognitive loss and increased pro‐inflammatory response. A) Experimental design for wild‐type (WT) and Cntnap4^+/−^ mice. B,C) Representative blots and quantification showing Cntnap4, Synapsin, Syntaxin, Synaptotagmin, and PSD‐95 expression in hippocampi of WT and Cntnap4^+/−^ mice. *n* = 6 per group. D,E) Representative traces and duration time in the open field for WT and Cntnap4^+/−^ mice. F) Number of arm entries in the Y maze for WT and Cntnap4^+/−^ mice. G) Representative traces in the water maze. H) Escape latency over a 5‐day training course. In the probe tests, mice were analyzed I) for escape latency, J) time spent in the target zone, and K) number of target crossing. *n* = 10 per group. L) Golgi staining of hippocampi. M) Quantification of the number of spines per 10 µm in hippocampi of WT and Cntnap4^+/−^ mice. *n* = 6 per group. N) Volcano plot showing the differential expressed genes (DEGs) between WT and Cntnap4^+/−^ mice. O) GO pathways enriched by downregulated DEGs in Cntnap4^+/−^ versus WT mice. P) Kyoto encyclopedia of genes and genomes (KEGG) pathways enriched by upregulated DEGs in Cntnap4^+/−^ versus WT mice. Q,R) The mRNA expression levels of *Tmem119*, *Cx3cr1*, *Csf1r*, *P2ry12*, *Il‐1b*, *Il‐6*, *Tnfa*, *Ifng*, and *Tgfb* in hippocampi of WT versus Cntnap4^+/−^ mice. *n* = 3 per group. S) Representative blots and quantification showing TMEM119, CX3CR1, CSF1R, IL‐1β and TNF‐α expression in hippocampi of WT and Cntnap4^+/−^ mice. *n* = 3 per group. T) Immunofluorescence staining and quantification of endpoint voxels, branch length and volume of Iba1‐positive cells in hippocampi of WT and Cntnap4^+/−^ mice. Scale bars, 40 µm. Magnified images are shown in the bottom row. Scale bars, 10 µm. *n* = 6−7. U) Immunofluorescence staining of Iba1 and CD68 cells in hippocampi of WT and Cntnap4^+/−^ mice. Scale bars, 100 µm. Magnified images are shown in the bottom row. Scale bars, 30 µm. Quantification of CD68 intensity was presented under the image. *n* = 9 per group. Results are expressed as the mean ± SEM. ^**^ *p* < 0.01, ^*^ *p* < 0.05 versus WT. Statistical significance was determined using the Student's *t*‐test.

To further verify that *Cntnap4* deficiency in female mice is associated with impaired cognitive function, we conducted behavioral and mechanistic analyses of female WT and heterozygous *Cntnap4* knockout (Cntnap4^+/−^) mice (Figure 3A,B). *Cntnap4* deficiency reduced hippocampal expression of PSD‐95, a major synaptic protein that is critical for plasticity and memory,^[^ ^16^ ^]^ while it showed no obvious effects on presynaptic proteins, including Synapsin, Syntaxin, and Synaptotagmin (Figure 3C). Furthermore, female Cntnap4^+/−^ mice spent less time in the center zone of the open field and exhibited reduced number of arm entries in the Y maze (Figure 3D–F). Consistent with the AAV‐Cntnap4 shRNA results, female Cntnap4^+/−^ mice needed more time to reach the platform in the 5‐day training phase and probe trial tests, with reduced duration times and crossings in the target quadrant but no changes in swimming speed (Figure 3G–K, Figure S8A–D, Supporting Information). Morphologically, female Cntnap4^+/−^ mice had a reduced number of spines in the hippocampus (Figure 3L,M). We also profiled the hippocampi from WT and Cntnap4^+/−^ female mice by RNA‐seq (Figure 3N, Figure S9A,B, Supporting Information), which showed that downregulated DEGs were enriched in GO pathways associated with synaptic plasticity, such as “Modulation of chemical synaptic transmission,” “Regulation of synaptic plasticity,” and “Dendritic spine organization,” while upregulated DEGs were enriched in KEGG pathways associated with immune response, such as “Complement and coagulation cascades,” “Antigen processing and presentation,” and “Cytokine‐cytokine receptor interaction” (Figure 3O,P). Additional DEG enriched pathways and the expression patterns for individual DEGs within relevant pathways are shown (Figures S10 and S11, Supporting Information). As further confirmation, both homeostatic genes (*Cx3cr1* and *Csf1r*) and inflammatory genes (*Il‐1b*, *Tnfa*, and *Ifng*), as well as the protein expressions of TMEM119, CX3CR1, CSF1R, IL‐1β, and TNF‐α were increased in the hippocampi of female Cntnap4^+/−^ mice (Figure 3Q–S). Consistently, microglia were activated in the hippocampi of female Cntnap4^+/−^ mice (Figure 3T). Increased hippocampal CD68 expression further verified the activation of microglia in female Cntnap4^+/−^ mice (Figure 3U). These results confirm that reduced synaptic plasticity and increased immune response result from *Cntnap4* deficiency in female mice.

### 2.4 GABA supplementation rescues *Cntnap4* deficiency‐induced cognitive impairment and microglia activation

**FIGURE 4:**
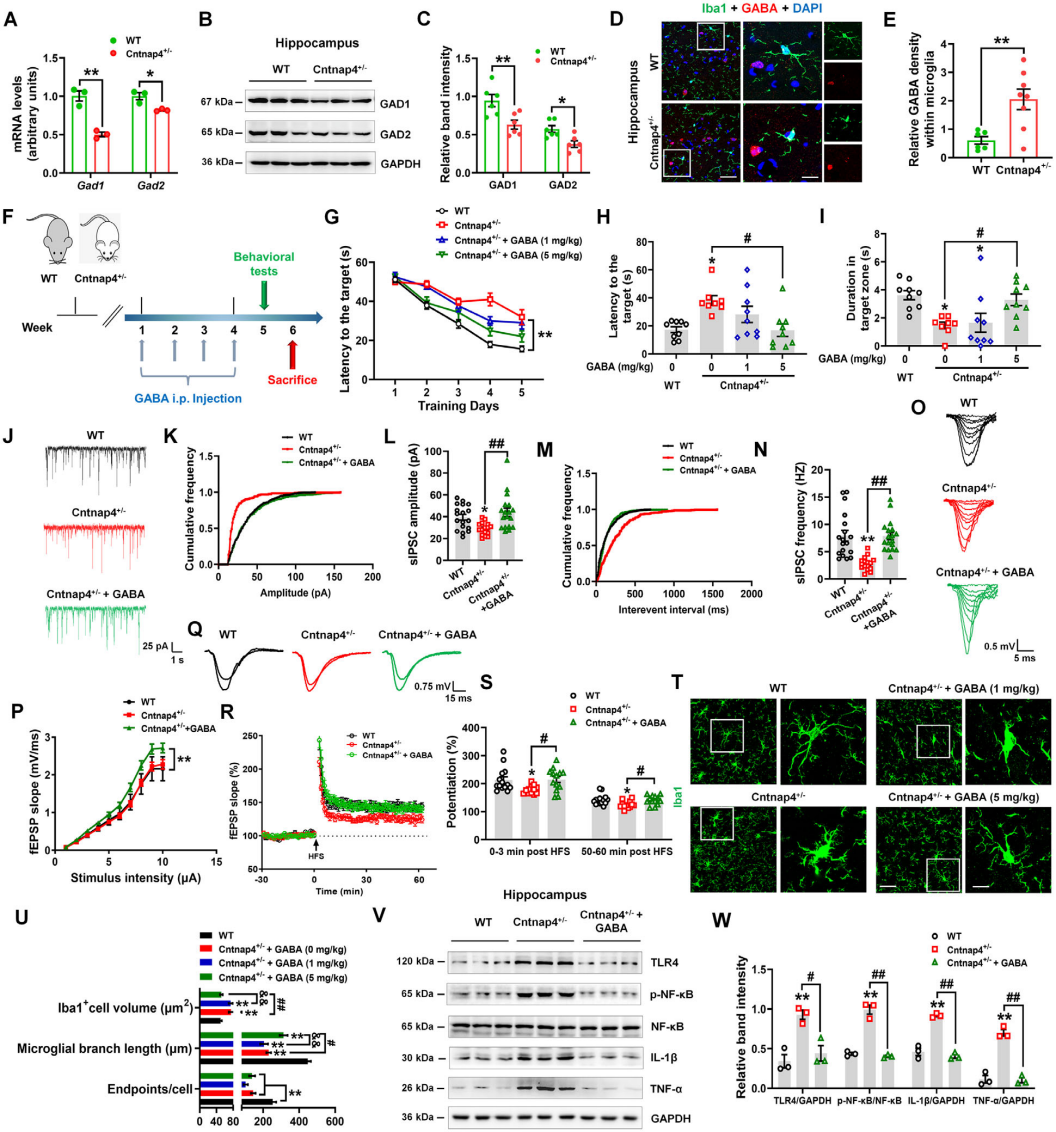
GABA supplementation rescues cognitive impairment and immune response in female Cntnap4^+/−^ mice. A) The mRNA expression levels of *Gad1* and *Gad2* in hippocampi of wild‐type (WT) and Cntnap4^+/−^ mice. *n* = 3 per group. B,C) Representative blots and quantification showing GAD1 and GAD2 expression in hippocampi of WT and Cntnap4^+/−^ mice. *n* = 6 per group. D,E) Immunofluorescence staining and quantification of Iba1 colocalization with GABA in hippocampi of WT and Cntnap4^+/−^ mice. *n* = 6−8. Scale bars, 40 µm. Magnified images are shown in the right column. Scale bars, 10 µm. F) Experimental design for GABA administration in Cntnap4^+/−^ mice. G) The escape latency over a 5‐day training course. In probe tests, mice were analyzed for the H) escape latency and I) time spent in the target zone. *n* = 8−9. J) Representative traces of GABA receptor‐mediated sIPSCs. All sIPSCs were recorded at a holding potential of −70 mV. K,L) Cumulative frequency plots of sIPSC amplitude, and quantitative analysis of the amplitude of GABA receptor‐mediated sIPSCs. *n* = 16−18. M,N) Cumulative frequency plots of the inter‐event interval, and quantitative analysis of the frequency of GABA receptor‐mediated sIPSCs. *n* = 16−18. O) Representative traces of input‐output stimulus intensities. P) Input–output relations generated by stimulating the SCs and recording in the CA1 stratum radiatum. *n* = 12−15. Q,R) Effects of GABA treatment in Cntnap4^+/−^ mice on the LTP at the SC‐CA1 synapses. The images show representative traces of fEPSP recordings of responses before and 50 min after high‐frequency stimulation (HFS; arrow). S) Quantitative analysis of data in R. The level of fEPSP potentiation was determined as a mean of 0−3 min and 50−60 min after high‐frequency stimulation. *n* = 12−14. T) Immunofluorescence staining of Iba1‐positive cells in hippocampi of GABA‐treated Cntnap4^+/−^ mice. Scale bars, 40 µm. Magnified images are shown in the right columns. Scale bars, 10 µm. U) Quantification of endpoint voxels, branch length, and volume of Iba1‐positive cells. *n* = 8−9 per group. V,W) Representative blots and quantification showing TLR4, p‐NF‐κB, NF‐κB, IL‐1β and TNF‐α expression in hippocampi of GABA‐treated Cntnap4^+/−^ mice. *n* = 3 per group. Results are expressed as the mean ± SEM. ^**^ *p* < 0.01, ^*^ *p* < 0.05 versus WT; ^##^ *p* < 0.01, ^#^ *p* < 0.05 versus Cntnap4^+/−^; ^&&^ *p* < 0.01 versus Cntnap4^+/−^ + GABA (1 mg kg^−1^). Statistical significance was determined using Student's *t*‐test (for panels (A, C, and E)) and one‐way ANOVA and Tukey's tests for post hoc comparisons (for panels (G–I, L, N, P, S, U, and W)).

Because *Cntnap4* deficiency disrupts GABAergic transmission and GABA has anti‐inflammatory functions,^[^ ^6^, ^17^ ^]^ we assessed the expression status and potential therapeutic role of GABA in female Cntnap4^+/−^ mice. The mRNA and protein expression of GAD1 and GAD2, two critical enzymes in GABA synthesis, were decreased in Cntnap4^+/−^ mice (Figure 4A–C). Moreover, the TLR4/NF‐κB pathway was activated in Cntnap4^+/−^ mice, which is consistent with increased microglial activation (Figure S12, Supporting Information). Surprisingly, however, we found increased GABA+ cells surrounding the microglia and enhanced GABA immunoreactivity within the microglia (Figure 4D,E), suggesting that GABA may be increased in Cntnap4^+/−^ mice as a compensatory response to defend the microglial immune response. On the basis of these findings, we tested the potential therapeutic role of GABA in shaping synaptic plasticity in Cntnap4^+/−^ mice (Figure 4F). Consistently, we found impaired spatial memory of Cntnap4^+/−^ mice in the water maze, as compared with WT (Figure 4G–I). Notably, 5 mg kg^−1^ GABA supplementation attenuated the latency time to the platform in the 5‐day training phase and probe trial test and increased the duration time in the target quadrant (Figure 4G–I). In contrast, GABA showed no obvious effects on behavioral performance in the open field and Y maze tests or on swimming speed in the water maze (Figures S13A–F, and S14A–D, Supporting Information). We also found reduced amplitude and frequency of hippocampal sIPSC and LTP induction in the Cntnap4^+/−^ mice, which were consistent with the results from AAV‐Cntnap4 shRNA injection (Figure 4J–S). Intriguingly, 5 mg kg^−1^ GABA supplementation rescued the decreased amplitude and frequency of hippocampal sIPSC and increased the field excitatory postsynaptic potentials (fEPSP) slope and LTP amplitude at both 0−3 min and 50−60 min after LTP induction in Cntnap4^+/−^ mice (Figure 4J–S). As expected, 5 mg kg^−1^ GABA supplementation significantly suppressed microglia activation, and decreased the protein expressions of TLR4/NF‐κB pathway, as well as IL‐1β and TNF‐α in the hippocampus of Cntnap4^+/−^ mice (Figure 4T–W). We also evaluated the anti‐inflammatory property of GABA in LPS‐treated microglial BV2 cells in vitro and found that GABA supplementation (50−1000 µM) significantly abrogated LPS‐induced *Il‐1b*, *Il‐6* and *Tnfa* mRNA expression (Figure S15A, Supporting Information). Moreover, 50 µM GABA inhibited TLR4/NF‐κB pathway activation and supernatant levels of IL‐1β, IL‐6, and TNF‐α in LPS‐treated BV2 cells (Figure S15B–D, Supporting Information). Therefore, these results support a therapeutic role for GABA supplementation in *Cntnap4* deficiency‐associated cognitive dysfunction.

### 2.5 Preparation, characterization, and brain‐targeting validation of BMNPs

**FIGURE 5:**
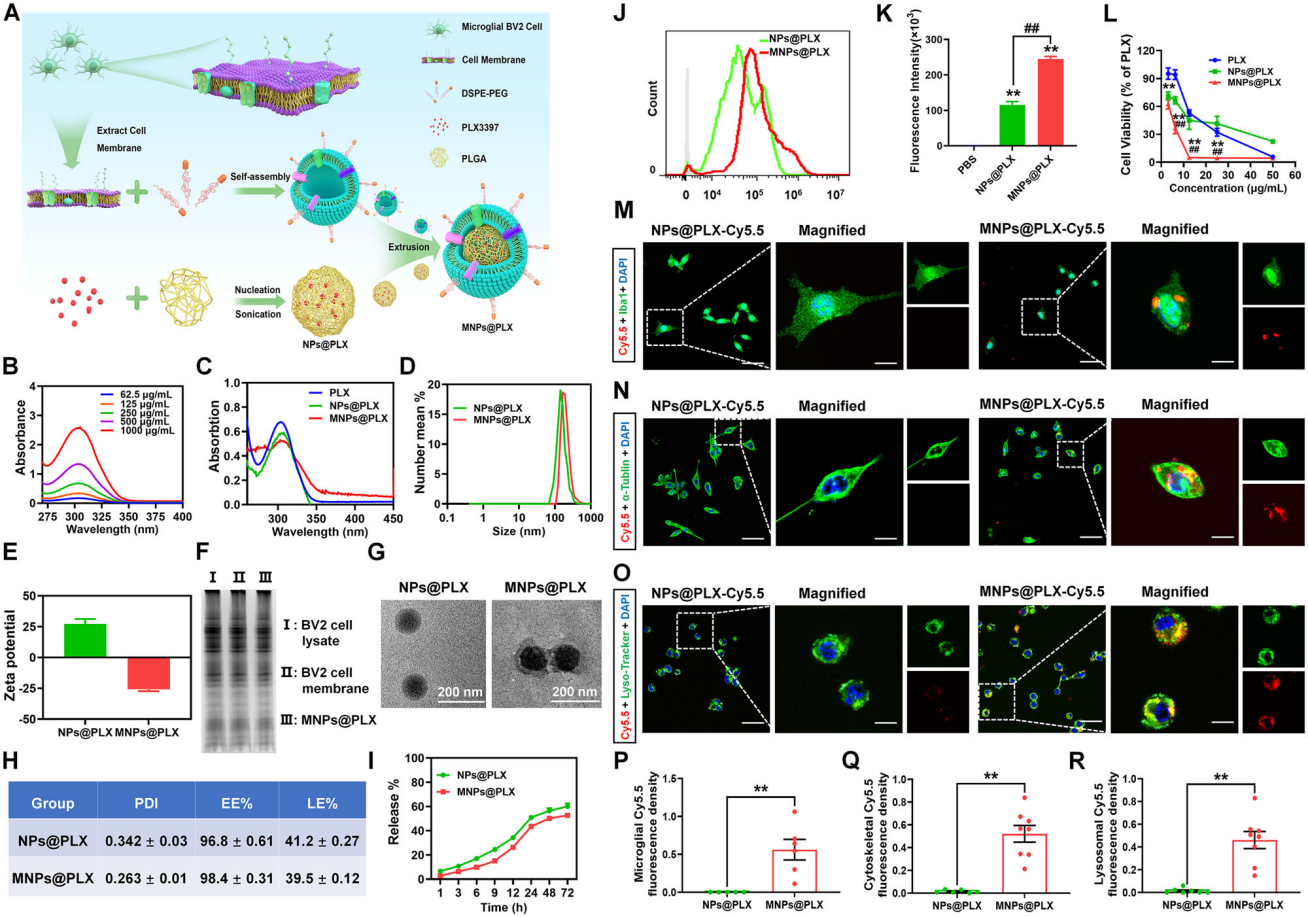
Biological effects of MNPs@PLX. A) Schematic illustration of the preparation of MNPs@PLX. B) UV–vis absorbance of PLX3397 at increasing doses. C) UV–vis absorbance of PLX3397, NPs@PLX and MNPs@PLX. D) Particle size analysis of NPs@PLX and MNPs@PLX. E) Zeta potential of NPs@PLX and MNPs@PLX. *n* = 3 per group. F) Representative blots of BV2 cell lysates, BV2 cell membranes, and MNPs@PLX. G) Representative transmission electron microscopy images. Scale bars, 200 nm. H) The polydispersity index (PDI), encapsulation efficiency (EE%), and loading efficiency (LE%) of NPs@PLX and MNPs@PLX were determined by UV spectrophotometry. *n* = 3 per group. I) The drug release kinetics of NPs@PLX and MNPs@PLX. *n* = 3 per group. J,K) Flow cytometry analysis of cellular uptake of NPs@PLX and MNPs@PLX. *n* = 3 per group. L) Cell viability of BV2 cells treated with PLX3397, NPs@PLX, or MNPs@PLX. M–O) Representative images of Iba1, cytoskeletal α‐Tubulin, and lysosomal tracker (Lyso‐tracker) in BV2 cells after 3 h incubation with Cy5.5‐labelled NPs@PLX or MNPs@PLX. Scale bars, 50 µm. Magnified images are shown in the right columns. Scale bars, 10 µm. P–R) Quantitative fluorescence analysis of M‐O. *n* = 5−8. Results are expressed as the mean ± SEM. ^**^ *p* < 0.01 versus control; ^##^ *p* < 0.01 versus NPs@PLX. Statistical significance was determined using one‐way ANOVA and Tukey's tests for post hoc comparisons (for panels (K and L)) and the Student's *t*‐test (for panels (P–R)).

Next, we sought to investigate whether interrupting microglia using PLX3397, a FDA‐approved CSF1R inhibitor, could also rescue the synaptic dysfunction and immune response in Cntnap4^+/−^ mice. The use of PLX3397, a well‐established microglial inhibitor, is limited because of its low efficiency at crossing the BBB.^[^ ^13^ ^]^ Therefore, we established a BMNPs platform (termed MNPs@PLX) for targeted delivery of PLX3397 into microglia (Figure 5A). The preparation of membrane‐biomimetic MNPs@PLX consisted of three steps: first, microglial BV2 cell membranes were extracted using ultracentrifugation; second, PLX3397‐coated PLGA nanoparticles were prepared as the core (termed NPs@PLX); and third, the isolated microglial BV2 cell membranes were coated on the surface of the nanoparticles by extrusion. The absorption spectra of PLX3397, NPs@PLX, and MNPs@PLX had the similar peaks, demonstrating that PLX3397 was efficiently loaded into NPs@PLX and MNPs@PLX (Figure 5B,C, Figure S16, Supporting Information). Dynamic light scattering results revealed that the average hydrodynamic diameter was higher for MNPs@PLX (190 nm) than for NPs@PLX (141 nm) (Figure 5D). The ζ‐potential of NPs@PLX (≈ +25 mV) showed a positive charge (≈ −25 mV) after being coated with cell membrane, and SDS‐PAGE, as well as TEM results, indicated that MNPs@PLX were successfully coated with BV2 cell membranes (Figure 5E–G). The polydispersity index (PDI), encapsulation efficiency (EE%), loading efficiency (LE%), and release behaviors before and after coating with BV2 cell membranes are shown in Figure 5H,I. Flow cytometry results of BV2 cells incubated with Cy5.5‐labelled NPs@PLX and MNPs@PLX suggested excellent targeting of MNPs@PLX (Figure 5J,K), and MNPs@PLX displayed enhanced toxic effects on BV2 cells (Figure 5L). Cellular uptake was further confirmed by co‐staining with Iba1 and cytoskeletal α‐Tubulin, and after coating with membranes, MNPs@PLX accumulated much more efficiently in the lysosomes, suggesting that it was successfully released upon degradation (Figure 5 M–R).

**FIGURE 6:**
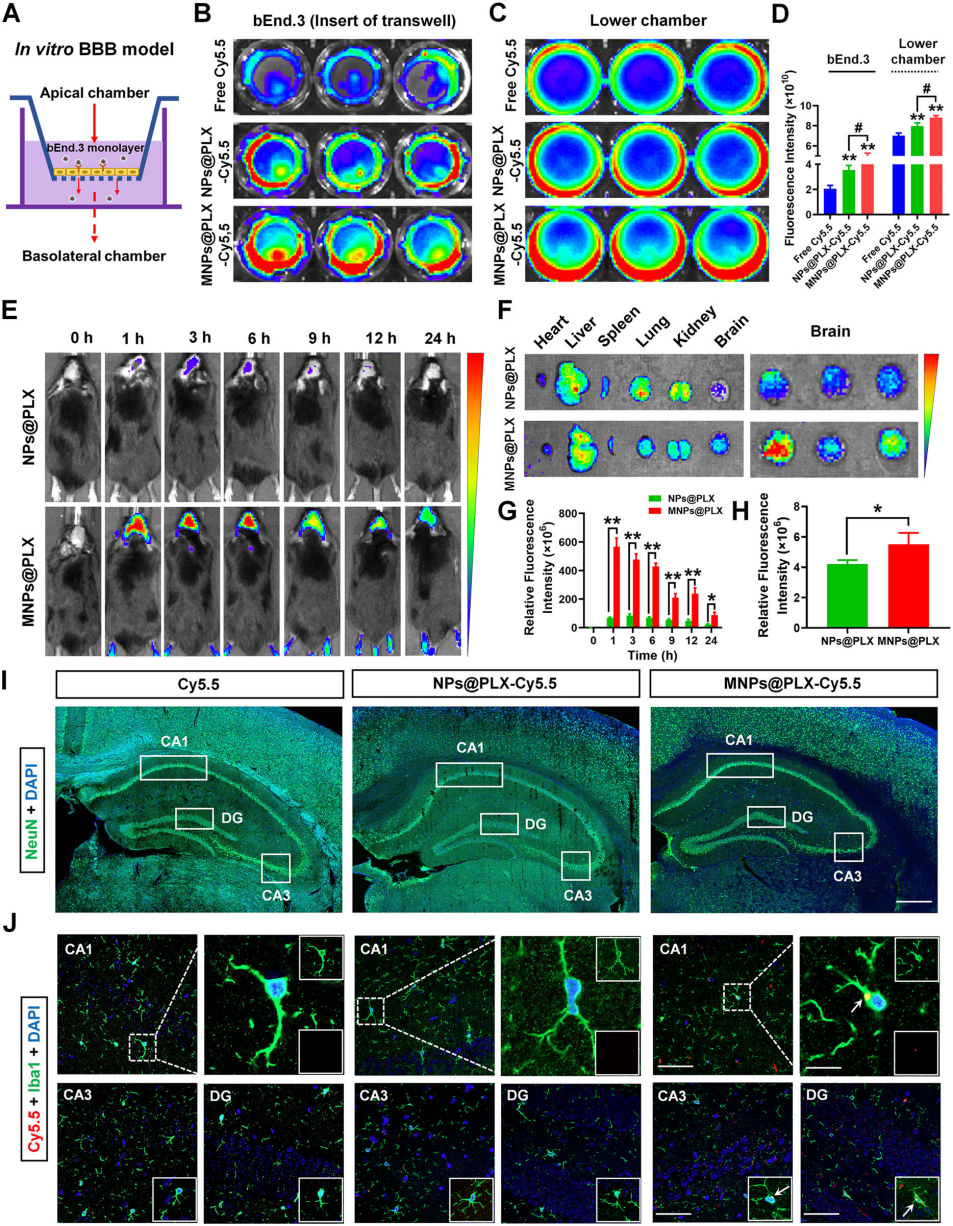
Brain‐targeted delivery of MNPs@PLX. A) Schematic diagram of the in vitro blood‐brain barrier (BBB) model. B–D) Fluorescence images and quantitation of the bEnd.3 monolayer and basolateral chamber in the transwell after 3 h incubation with Cy5.5, NPs@PLX‐Cy5.5, or MNPs@PLX‐Cy5.5. *n* = 3 per group. E) Real time fluorescence imaging of mice after intravenous injection of Cy5.5‐labelled NPs@PLX or MNPs@PLX. F–H) Ex vivo imaging and corresponding fluorescence analysis of sacrificed tissues (heart, liver, spleen, lung, kidney, and brain) after intravenous injection of Cy5.5‐labelled NPs@PLX or MNPs@PLX. *n* = 3 per group. I) Representative images of NeuN+ cells in the CA1, CA3, and DG areas of the hippocampus. Scale bars, 200 µm. J) Representative images of Iba1 staining at 6 h post‐injection in hippocampal CA1, CA3, and DG areas of mice treated with Cy5.5, NPs@PLX‐Cy5.5, or MNPs@PLX‐Cy5.5. Scale bars, 40 µm in CA1, CA3, and DG. Magnified images are shown in the right panel for CA1. Scale bars, 10 µm. White arrows in the enlarged details show the presence of nanoparticles in microglia. Results are expressed as the mean ± SEM. ^**^ *p* < 0.01, ^*^ *p* < 0.05 versus Control; ^#^ *p* < 0.05 versus MNPs@PLX. Statistical significance was determined using one‐way ANOVA and Tukey's tests for post hoc comparisons (for panel (D)) and the Student's *t*‐test (for panels (G and H)).

We further tested the brain targeting and biodistribution of MNPs@PLX. Our established in vitro BBB permeability test showed that fluorescence signals in the bEND.3 cell monolayer and lower chamber were higher after incubation with Cy5.5‐lablled MNPs@PLX than NPs@PLX, suggesting higher BBB permeability of MNPs@PLX (Figure 6A–D). In vivo fluorescence imaging and ex vivo results further support the stable and persistent brain distribution of MNPs@PLX compared with NPs@PLX (Figure 6E–H). Although MNPs@PLX also accumulated in the liver after administration at 24 h, its signal gradually decreased at 48 and 72 h (Figure S17A,B, Supporting Information). Furthermore, MNPs@PLX but not NeuN or GFAP, were obviously accumulated in the microglia in the CA1, CA3, and DG regions of the hippocampus (Figure 6I,J, Figure S18A,B, Supporting Information), and flow cytometry results also supported the microglial targeting specificity of the BMNPs strategy (Figure S19A,B, Supporting Information). MNPs@PLX administration in WT mice also inhibited *Csf1r* and *Cx3cr1* expression in the hippocampus (Figure S20A,B, Supporting Information). Additionally, MNPs@PLX administration had no obvious effects on the behavioral performance in the open field, Y maze, and water maze (Figure S21A–N, Supporting Information). These findings indicate that our established BMNPs strategy successfully penetrates the BBB and effectively localizes PLX397 to hippocampal microglia.

### 2.6 MNPs@PLX ameliorates cognitive decline and impaired synaptic plasticity by suppressing pro‐inflammatory events in female Cntnap4^+/−^ mice

**FIGURE 7:**
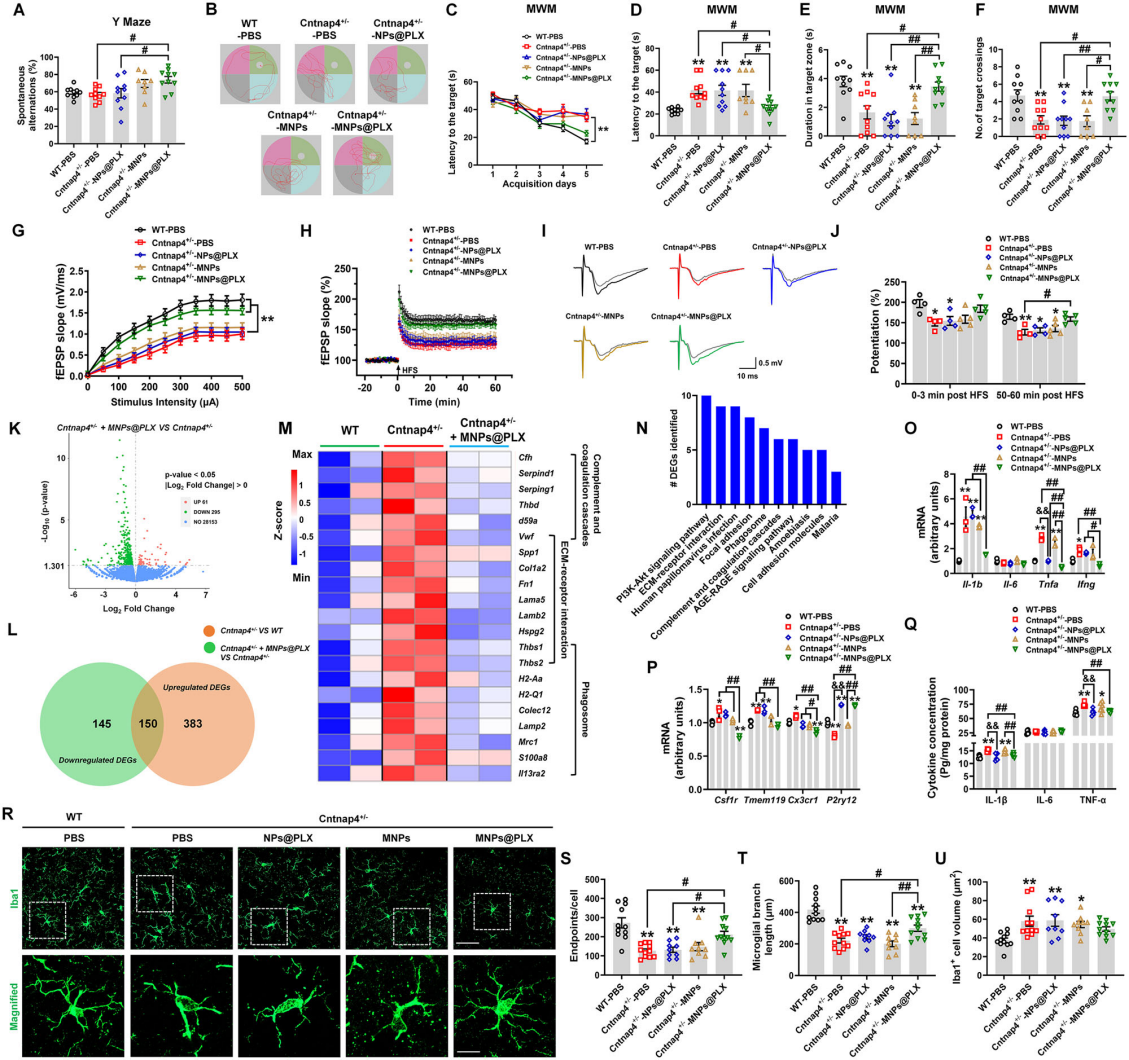
MNPs@PLX attenuates cognitive impairment, impaired synaptic plasticity, and pro‐inflammatory reaction in female Cntnap4^+/−^ mice. A) Y maze results for Cntnap4^+/−^ mice treated with NPs@PLX, MNPs or MNPs@PLX are presented as spontaneous alternations (%). B) Representative traces in the water maze. C) Escape latency over a 5‐day training course. In probe tests, mice were analyzed for the D) escape latency, E) time spent in the target zone, and F) number of target crossing. *n* = 8−10. G) Input–output relations generated by stimulating the SCs in the CA1 stratum radiatum. *n* = 4−7. H) LTPs at SC‐CA1 synapses in hippocampi of Cntnap4^+/−^ mice treated with NPs@PLX, MNPs or MNPs@PLX. I) Representative traces of fEPSP recordings of responses before and 50 min after high‐frequency stimulation (HFS; arrow). J) Quantitative analysis of LTP data in (H). The level of fEPSP potentiation was determined at a mean of 0−3 min and 50−60 min after high‐frequency stimulation. *n* = 4−5. K) Volcano plot showing the differential expressed genes (DEGs) in Cntnap4^+/−^ + MNPs@PLX versus Cntnap4^+/−^mice. L) DEGs in *Cntnap4* deficiency versus wild‐type (WT) mice that were reversed by MNPs@PLX. M) Hierarchical clustering of DEGs enriched in “Complement and coagulation cascades,” “ECM‐receptor interaction,” and “Phagosome” pathways. N) Kyoto encyclopedia of genes and genomes (KEGG) pathways enriched by MNPs@PLX‐reversed DEGs. O,P) mRNA expression levels of *Il‐1b*, *Il‐6*, *Tnfa*, *Ifng*, *Csf1r*, *Tmem119*, *Cx3cr1*, and *P2ry12* in hippocampi of Cntnap4^+/−^ mice treated with NPs@PLX, MNPs or MNPs@PLX. *n* = 3 per group. Q) The protein expression of IL‐1β, IL‐6, and TNF‐α in hippocampi of Cntnap4^+/−^ mice treated with NPs@PLX, MNPs or MNPs@PLX. *n* = 6 per group. R) Immunofluorescence staining of Iba1‐positive cells in hippocampi of Cntnap4^+/−^ mice treated with NPs@PLX, MNPs or MNPs@PLX. Scale bars, 40 µm. Magnified images are shown in the bottom panels. Scale bars, 10 µm. Quantification of S) endpoint voxels, T) branch length, and U) volume of Iba1‐positive cells. *n* = 9−11 per group. Results are expressed as the mean ± SEM. ^**^ *p* < 0.01, ^*^ *p* < 0.05 versus WT‐PBS; ^##^ *p* < 0.01, ^#^ *p* < 0.05 versus Cntnap4^+/−^ + MNPs@PLX; ^&&^ *p* < 0.01 versus Cntnap4^+/−^ + NPs@PLX. Statistical significance was determined using one‐way ANOVA and Tukey's tests for post hoc comparisons.

Next, we explored the therapeutic capacity of MNPs@PLX in female Cntnap4^+/−^ mice. Equivalent molecular doses of NPs@PLX, MNPs@PLX, MNPs, or the control vehicle were administered intravenously in female Cntnap4^+/−^ mice for 12 continuous days. The next day, behavioral tests were performed to evaluate the exploratory activity and cognitive function of the mice (Figure S22A, Supporting Information). MNPs@PLX did not alter the behavioral performance in the open field (Figure S22B–F, Supporting Information), but it increased the spontaneous alternations (Figure 7A), reduced the latency time to the platform in the 5‐day training phase and probe trial test and increased the duration time and crossings in the target quadrant (Figure 7B–F). There was no effect on the swimming speed (Figure S23A–D, Supporting Information).

Consistent with its ability to improve spatial memory in the water maze, MNPs@PLX increased the fEPSP slope and LTP amplitude at 50−60 min after LTP induction (Figure 7G–J). Therefore, we performed RNA‐seq of hippocampi from WT, Cntnap4^+/−^ and Cntnap4^+/−^ + MNPs@PLX mice and identified 150 DEGs that were increased by *Cntnap4* deficiency and reversed by MNPs@PLX treatment (Figure 7K,L, Figure S24, Supporting Information). Notably, these DEGs were enriched in immune related pathways, such as “Complement and coagulation cascades” “ECM‐receptor interaction,” and “Phagosome” (Figure 7 M,N). Consistently, MNPs@PLX suppressed the expression of inflammatory (*Il‐1b*, *Tnfa*, and *Ifng*) and homeostatic (*Csf1r*, *Tmem119*, and *Cx3cr1*) genes and reduced cytokine (IL‐1β and TNF‐α) expression in the hippocampi of Cntnap4^+/−^ mice as compared with Cntnap4^+/−^ mice with or without NPs@PLX and MNPs (Figure 7O–Q). Furthermore, MNPs@PLX increased the endpoints per cell and microglial branch length without affecting the microglial volume in the hippocampi of Cntnap4^+/−^ mice (Figure 7R–U). Moreover, MNPs@PLX also increased microglial GABA expression and the expression of *Esr1* (encoding estrogen receptor 1) and *Esr2* (encoding estrogen receptor 2) as compared with Cntnap4^+/−^ mice with or without NPs@PLX and MNPs (Figures S25 and S26, Supporting Information), suggesting MNPs@PLX treatment works possibly through regulating GABA and estrogen‐related pathways.

Because we also observed low‐level MNPs@PLX localization in the liver, lung, and kidney of mice after intravenous administration, we investigated potential adverse effects of MNPs@PLX on non‐targeted organs. However, we did not detect pathological changes in the liver, heart, kidney, or lung as evaluated by H&E staining, and there were no detectable alterations in serum biochemical indicators of liver or kidney function after NPs@PLX or MNPs@PLX administration (Figure S27 and Table S1, Supporting Information). Taken together, these data indicate that MNPs@PLX attenuates cognitive decline, dysfunctional synaptic plasticity, and microglia activation in female Cntnap4^+/−^ mice.

### 2.7 Inactivation of MAOA may be associated with MNPs@PLX's anti‐inflammatory effects in female Cntnap4^+/−^ mice

**FIGURE 8:**
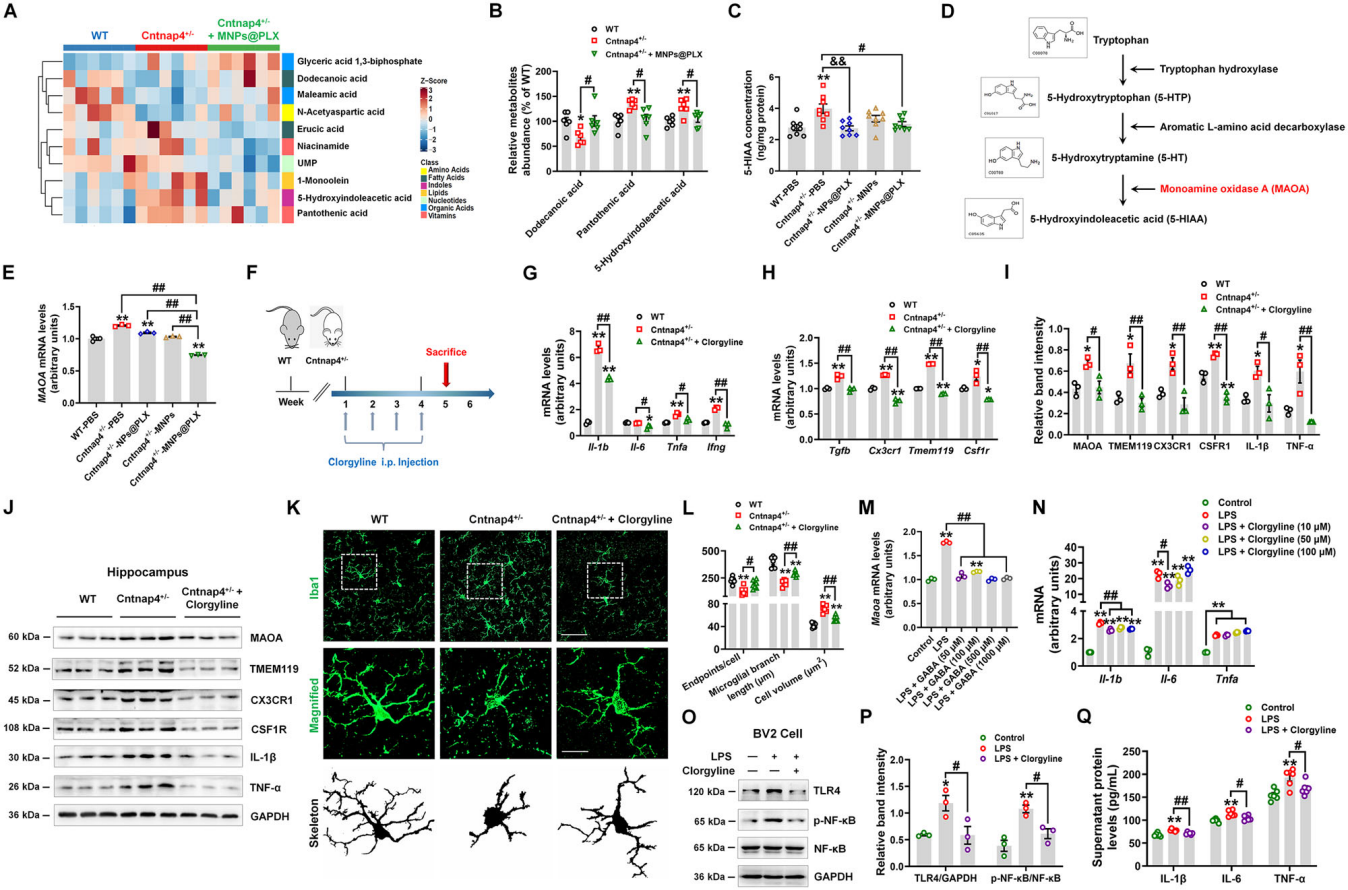
MNPs@PLX suppresses the pro‐inflammatory reaction by modulating MAOA in female Cntnap4^+/−^ mice. A) Hierarchical clustering of differential metabolites among wild‐type (WT), Cntnap4^+/−^ and Cntnap4^+/−^ +MNPs@PLX mice. B) The expression levels of Dodecanoic acid, Pantothenic acid, and 5‐HIAA in the hippocampus from the metabonomic results. C) The expression levels of 5‐HIAA in the hippocampus were examined by ELISA. D) Schematic illustration of tryptophan metabolism to produce 5‐Hydroxyindoleacetic acid (5‐HIAA). MAOA (highlighted in red) catalyzes the last step. E) The mRNA expression level of *Maoa* in hippocampi of Cntnap4^+/−^ mice treated with NPs@PLX, MNPs, or MNPs@PLX. *n* = 3 per group. F) Experimental design for clorgyline administration in Cntnap4^+/−^ mice. G,H) The mRNA expression levels of *Il‐1b*, *Il‐6*, *Tnfa*, *Ifng*, *Tgfb*, *Cx3cr1*, *Tmem119*, and *Csf1r* in hippocampi of Cntnap4^+/−^ mice treated with clorgyline. *n* = 3 per group. I,J) Representative blots and quantification showing MAOA, TMEM119, CX3CR1, CSF1R, IL‐1β, and TNF‐α expression in hippocampi of Cntnap4^+/−^ mice treated with clorgyline. *n* = 3 per group. K) Immunofluorescence staining of Iba1‐positive cells in hippocampi of Cntnap4^+/−^ mice treated with clorgyline. Scale bars, 40 µm. Magnified images are shown in the middle row, and skeletal diagrams of Iba1‐positive cells are shown in the bottom row. Scale bars, 10 µm. L) Quantification of endpoint voxels, branch length and volume of Iba1‐positive cells. *n* = 6−7. M) The mRNA expression level of *Maoa* in LPS‐treated cells with different concentrations of GABA (50, 100, 500, and 1000 µM). *n* = 3 per group. N) The mRNA expression levels of *Il‐1b*, *Il‐6*, and *Tnfa* in LPS‐treated cells with different concentrations of clorgyline (10, 50, and 100 µM). *n* = 3 per group. O,P) Representative blots and quantification showing TLR4, p‐NF‐κB, and NF‐κB expression in cells treated with LPS and/or 10 µM clorgyline. *n* = 3 per group. Q) Cellular supernatant levels of IL‐1β, IL‐6, and TNF‐α in cells treated with LPS and/or 10 µM clorgyline. *n* = 6 per group. Results are expressed as the mean ± SEM. ^**^ *p* < 0.01, ^*^ *p* < 0.05 versus WT or control; ^##^ *p* < 0.01, ^#^ *p* < 0.05 versus Cntnap4^+/−^ + MNPs@PLX or Cntnap4^+/−^ + clorgyline or LPS + clorgyline; ^&&^ *p* < 0.01 versus Cntnap4^+/−^ + NPs@PLX. Statistical significance was determined using one‐way ANOVA and Tukey's tests for post hoc comparisons.

To explore the mechanism of MNPs@PLX's anti‐inflammatory activity in female Cntnap4^+/−^ mice, we evaluated its metabolomic effects. There was a distinct metabolomic signature between WT and Cntnap4^+/−^mice, with 6 differential metabolites (2 decreased and 4 increased in Cntnap4^+/−^ versus wild‐type (WT)) (Figure S28A,B, Supporting Information). Furthermore, there were 10 differential metabolites among WT, Cntnap4^+/−^ and Cntnap4^+/−^ + MNPs@PLX mice (Figure 8A, Figure S28C,D, Supporting Information). MNPs@PLX was confirmed to reverse the decrease in Dodecanoic acid and increase in Pantothenic acid and 5‐Hydroxyindoleacetic acid (5‐HIAA) caused by *Cntnap4* deficiency (Figure 8B, Figure S28E, Supporting Information). We further confirmed that MNPs@PLX blunted hippocampal 5‐HIAA levels in Cntnap4^+/−^ and WT mice (Figure 8C, Figure S28F, Supporting Information). 5‐HIAA is the end‐product of tryptophan metabolism in which tryptophan, 5‐hydroxytryptophan (5‐HTP), and 5‐hydroxytryptamine (serotonin, 5‐HT) are catalyzed by tryptophan hydroxylase, aromatic L‐amino acid decarboxylase and monoamine oxidase A (MAOA), respectively (Figure 8D). Therefore, these results support an effect of MNPs@PLX on tryptophan metabolism in Cntnap4^+/−^ mice (Figure S28G, Supporting Information). As additional confirmation of this possibility, MAOA was also reduced by MNPs@PLX administration in the hippocampi of Cntnap4^+/−^ mice (Figure 8E). To investigate whether MAOA inactivation may explain MNPs@PLX's anti‐inflammatory effects, we injected a MAOA inhibitor (clorgyline) into Cntnap4^+/−^ mice for 4 weeks (Figure 8F). Interestingly, clorgyline reversed the effect of *Cntnap4* deficiency on the expression of inflammatory (*Il‐1b*, *Il‐6*, *Tnfa*, and *Ifng*) and homeostatic (*Cx3cr1*, *Tmem119*, and *Csf1r*) genes, as well as the protein expressions of MAOA, TMEM119, CX3CR1, CSF1R, IL‐1β and TNF‐α (Figure 8G–J). Besides, it increased microglial GABA concentration and inhibited microglia activation in Cntnap4^+/−^ mice (Figure 8K,L, Figure S29, Supporting Information). Moreover, GABA supplementation also reduced LPS‐induced *Maoa* expression in vitro (Figure 8 M). Clorgyline also decreased *Il‐1b*, *Il‐6*, and *Tnfa* expression, TLR4/NF‐κB pathway activation, and supernatant levels of IL‐1β, IL‐6, and TNF‐α in LPS‐treated BV2 cells (Figure 8N–Q). Collectively, these findings support the possibility that MAOA activation induces pro‐inflammatory events that underlie MNPs@PLX's anti‐inflammatory effects in female Cntnap4^+/−^ mice.

## 3 DISCUSSION

Previously, *Cntnap4* deficiency has been linked to ASDs, schizophrenia, and epilepsy as a result of its predominant role in regulating neurological development and GABAergic synaptic transmission.^[^ ^5^, ^6^ ^]^ Our group recently provided evidence that ablation of *Cntnap4* in DA neurons contributes to PD pathogenesis via regulation of mitophagy, and *Cntnap4* deficiency alters fear memory by modulating GABAergic transmission in basolateral amygdala.^[^ ^8^, ^18^ ^]^ Additionally, a *CNTNAP4* CNV polymorphism has been associated with aging and aging‐related conditions in females, such as cognitive impairment and LOAD,^[^ ^7^ ^]^ which is consistent with our findings demonstrating that hippocampal *Cntnap4* deficiency damages cognitive function and synaptic plasticity in female mice. In this study, we describe the dysfunction of GABAergic transmission as a link between microglia activation and cognitive impairment caused by *Cntnap4* deficiency. First, Cntnap4 is highly expressed in developing murine interneurons (mainly PV positive neurons), and it influences GABAergic transmission by regulating the maturation of interneuron, since the percentage of Cntnap4‐positive PV neurons increases with age from 64% (P21) to 94% (P60).^[^ ^6^ ^]^ Consistently, we observed impaired hippocampal inhibitory transmission in both AAV‐Cntnap4 shRNA‐injected mice and Cntnap4^+/−^ mice, and GABA supplementation reverses impaired inhibitory transmission in Cntnap4^+/−^ mice. Second, as a critical inhibitory transmitter, GABA exhibits anti‐inflammatory property, including the ability to diminish inflammatory responses in animal models of multiple sclerosis and cancer and LPS‐treated bovine mammary epithelial cells by inhibiting the TLR4/NF‐κB pathway.^[^ ^12^, ^17^, ^19^ ^]^ Here we also found GABA supplementation suppressed the pro‐inflammatory response and microglia activation mediated by TLR4/NF‐κB pathway in vivo of *Cntnap4* deficiency mice and in in vitro of LPS‐treated microglial BV2 cells. To this end, we determined that GABA supplementation improves cognitive function and synaptic plasticity in Cntnap4^+/−^ mice. We observed the increased GABA immunoreactivity in microglia of Cntnap4^+/−^ mice, and we interpreted it may be a compensatory response. However, we found decreased GABAergic transmission (sIPSC amplitude and frequency) in the hippocampus of Cntnap4^+/−^ mice, and GABA supplementation rescues it. Thus, increased GABA in microglia of Cntnap4^+/−^ mice may be insufficient to suppress the pro‐inflammatory response and cognitive impairment. Additionally, MNPs@PLX and clorgyline further increased microglial GABA content, suggesting microglial GABA is responsible for the anti‐inflammatory effects of MNPs@PLX and MAOA inhibitor.

The cognitive impairment observed in this study was mainly in female mice. There is a strong link between sex hormones, cognitive dysfunction, and inflammation. It has been indicated that low levels of estrogens have been implicated in the etiology of dementia in women, including AD patients,^[^ ^20^ ^]^ suggesting estrogens deficiency may lead to cognitive decline. Women who have had their ovaries removed exhibit cognitive decline and high levels of pro‐inflammatory cytokines, such as IL‐1 and TNF‐α.^[^ ^21^ ^]^ Mechanistically, estrogen could suppress inflammation by inhibiting NF‐κB activity, NLRP3 inflammasome, and so forth.^[^ ^22^ ^]^ In this study, we found decreased estradiol in the hippocampus of *Cntnap4* knockdown mice, suggesting *Cntnap4* insufficient may decrease the levels of estrogen. We also found increased hippocampal PGE2 in *Cntnap4* knockdown mice. Recent study reported that in aged macrophages, increased PGE2 signaling through EP2 receptor induces the sequestration of glucose into glycogen, leading to lower glucose flux and energy insufficiency.^[^ ^23^ ^]^ While blockade of PGE2‐EP2 signaling inhibits the inflammation and reverses cognitive aging.^[^ ^23^ ^]^ Thus, we propose that decreased estradiol and increased PGE2 may underline the sex‐specific effects of *Cntnap4* deficiency on cognitive function. Further studies are needed to uncover the mechanism of differences in female and male mice with *Cntnap4* deficiency. In this study, we found the amplitude and frequency of sIPSC were decreased in female mice injected with Cntnap4 shRNA, while APs showed no difference. We conclude this may be due to sIPSC reflecting the activity of inhibitory synaptic transmission, while APs reflect the individual neuron activity. This phenomenon was also reported by other groups.^[^ ^24^ ^]^

To reveal the role of microglia in cognitive impairment due to *Cntnap4* deficiency, we established a biomimetic microglial delivery strategy. Because microglia are resistant to manipulation by recombinant viruses, such as lentiviruses and AAVs, and the BBB hinders drug delivery to the brain, microglial targeted therapy is a challenging endeavor.^[^ ^25^ ^]^ Recently, photoresponsive vaccine‐like chimeric antigen receptors and neural progenitor cell or stem cell‐derived extracellular vesicle systems have been designed to targeting microglia in inflammation‐related depression and cerebral ischemia.^[^ ^26^ ^]^ Inspired by these findings, we designed a BMNPs system to deliver PLX3397 for inflammation intervention. Our in vitro, ex vivo, and in vivo results confirmed the high efficacy of delivering PLX3397 to microglia using this approach. We revealed that MNPs@PLX significantly improves impaired cognitive function and synaptic plasticity by inhibiting proinflammatory response in female Cntnap4^+/−^ mice. MNPs@PLX seems not to affect the exploratory and memory behaviors of normal mice, which is consistent with previous study.^[^ ^13^ ^]^ However, we need further study to verify whether the clearance of microglia by MNPs@PLX may cause detrimental effect on normal brain function.

In this study, nanoparticles coated with BV2 cell membrane improve brain targeting. However, as a main organ responsible for metabolic function, the liver is susceptible to the circulating nanoparticles. It has been reported that up to 60% of polymer nanospheres accumulated in the liver within 5 min of intravenous administration,^[^ ^27^ ^]^ and preclinical studies showed that large amounts of nanotherapeutic drugs accumulate in the liver.^[^ ^28^ ^]^ In this work, MNPs@PLX used PLGA as a carrier that has good biodegradability in vivo,^[^ ^29^ ^]^ and we found the signal of MNPs@PLX gradually degraded after intravenous administration. MNPs@PLX has good homologous targeting, whilst we may need to perform similar experiments to evaluate the delivery difference between primary microglia and BV2 cells in the future.

Although PLX3397 is a well‐known CSF1R inhibitor with an excellent capacity to clear microglia,^[^ ^13^ ^]^ PLX5622, a highly selective brain‐penetrant CSF1R inhibitor that depletes microglia, has been recently synthesized.^[^ ^30^ ^]^ However, we selected PLX3397, rather than PLX5622 for this study because PLX3397 is FDA‐approved and has been shown to serve as a responsive and effective treatment for patients.^[^ ^14^, ^31^ ^]^ In accordance with previous reports,^[^ ^32^, ^33^ ^]^ PLX3397 effectively depleted microglia. Furthermore, we demonstrated that the novel anti‐inflammatory effects of PLX3397 may be associated with inhibition of MAOA. In this study, we found that MNPs@PLX reverses *Cntnap4* deficiency and increases hippocampal 5‐HIAA levels. As an upstream precursor, 5‐HT has been identified to be an anti‐inflammatory neuromodulator in the brain.^[^ ^34^ ^]^ Though 5‐HIAA was previously thought to be an inactive end‐product of tryptophan catabolism, recently, it has been indicated to be an active serotonin metabolite that ameliorates memory decline by inducing neprilysin in AD.^[^ ^35^ ^]^ Notably, platelet‐derived 5‐HIAA is a novel ligand for G‐protein‐coupled receptor GPR35 and has a role in GPR35‐mediated neutrophil recruitment to inflamed tissue.^[^ ^36^ ^]^ In this study, we demonstrated that increased 5‐HIAA levels associated with *Cntnap4* deficiency may recruit microglia to induce inflammation, and that inhibition of MAOA reduces 5‐HIAA levels and suppresses proinflammatory inflammation. Usually, MAOA is mainly responsible for metabolism of 5‐hydroxytryptamine, and MAOB is involved in dopamine degradation. Studies also indicate MAOB, but not MAOA, is responsible for astrocytic GABA‐mediated tonic inhibitory currents,^[^ ^37^ ^]^ suggesting monoamine oxidase may affect GABAergic transmission. In this study, we found the expression of microglial GABA is increased in the hippocampus of Cntnap4^+/−^ mice, possibly due to compensatory response. While clorgyline further increased microglial GABA and decreased pro‐inflammatory response. These results reveal that MAOA is effective on GABA. Further study is needed to clarify this hypothesis. Microglial‐targeted delivery also has the advantage that it permits a more cost‐effective use of PLX3397, which is usually given in chow at 290 mg kg^−1^ or higher dosage to deplete microglia.^[^ ^13^, ^33^, ^38^ ^]^

Overall, our study highlights the role of Cntnap4 and its ability to mediate GABAergic transmission in hippocampal memory processing. In addition, our study suggests that the biomimetic microglial delivery strategy may represent an innovative tool for memory decline intervention.

## 4 METHODS

### 4.1 Materials

Anti‐Cntnap4 (bs‐11076R‐2) and Cntnap4 (orb544737) antibodies were purchased from Bioss (Beijing, China) and Biorbyt LLC (San Francisco, CA, USA), respectively. Anti‐Synapsin (#5297), Syntaxin (#18572), Synaptotagmin (#14558), NF‐κB p65 (#8242), Phospho‐NF‐κB p65 (Ser536) (#3033), GAD1 (#41318), GAD2 (#5843) and PSD‐95 (#3450) antibodies were purchased from Cell Signaling Technology (Danvers, MA, USA). Anti‐MAOA (ab126751) antibody was purchased from Abcam (Cambridge, MA, USA). Anti‐TLR4 (#AF7017) antibody was purchased from Affinity Biosciences (Cincinnati, OH, USA). Anti‐Iba1 (019−19741) antibody was purchased from FUJIFILM Wako (Osaka, Japan). Anti‐GFAP (MAB360) and NeuN (MAB377) antibodies were purchased from Millipore (Billerica, MA, USA). Anti‐TMEM119 (66948‐1‐Ig), CX3CR1 (13885‐1‐AP), CSF1R (25949‐1‐AP), IL‐1β (16806‐1‐AP), TNF‐α (17590‐1‐AP), and GAPDH (60004−1) antibodies were purchased from Proteintech Group (Rosemont, IL, USA). DyLight 488 goat anti‐mouse IgG (H + L) (70‐ GAM4882) and DyLight 594 goat anti‐rabbit IgG (H + L) (70‐GAR5942) antibodies were purchased from Multi Sciences (Hangzhou, China). Horseradish peroxidase (HRP)‐labeled goat anti‐rabbit IgG and HRP‐labeled goat anti‐mouse IgG were purchased from Beyotime Biotechnology (Shanghai, China). LPS (L2630), GABA(A5835), MAO‐A inhibitor (Clorgyline, M3778) were purchased from Sigma‐Aldrich (St Louis, MO, USA). PLX3397 (S7818) was purchased from Selleck (Houston, USA). PLGA (lactide, glycolide (75:25)) and 1,2‐distearoyl‐sn‐glycero‐3‐phosphoethanolamine‐*N*‐folatec (DSPE) poly (ethylene glycol) (PEG) 2000 (DSPE‐PEG2000) were purchased from Xi'an ruixi Biological Technology Co. Ltd. (Xi'an, China).

### 4.2 Cell culture and drug treatment

BV2 cells, SH‐SY5Y cells, and MN9D cells were purchased from American Type Culture Collection (ATCC, Manassas, VA, USA) and were cultured with DMEM medium containing 10% fetal bovine serum (GIBCO, Carlsbad, CA, USA), 1% 200 U mL^−1^ penicillin (Beyotime Biotechnology), and 1% 200 mg mL^−1^ streptomycin (Beyotime Biotechnology) at 37°C under 5% CO_2_ air in an incubator. Primary microglia were obtained according to our previous study,^[^ ^39^ ^]^ and they were cultured in DMEM/F12 (GIBCO, Carlsbad, CA, USA) supplemented with 10% FBS and GM‐CSF at 37°C in 5% CO_2_. Primary astrocytes were obtained as we described previously,^[^ ^40^ ^]^ and they were cultured in DMEM/F12 (GIBCO, Carlsbad, CA, USA) supplemented with 10% FBS at 37°C in 5% CO_2_. LPS (1 µg mL^−1^) was used to treat BV2 cells for 6 h to induce inflammation. GABA (50 mM stock concentration) was dissolved in 0.01 M PBS, and clorgyline was dissolved in dimethylsulphoxide (DMSO, 100 mM stock concentration). For detecting the anti‐inflammatory effects of GABA and clorgyline, the medium was removed after 6 h LPS treatment, and the cells were then treated with different concentrations of GABA (50, 100, 500, and 1000 µM) or clorgyline (10, 50, and 100 µM) for another 24 h.

### 4.3 Extraction of microglial BV2 cell membranes

BV2 cell membranes were obtained according to a previously reported extrusion approach.^[^ ^41^ ^]^ Briefly, RIPA lysis buffer was added to BV2 cells for 30 min, and the cells were sonicated (VCX130 ultrasonics processor, USA) for 20 s on ice. After centrifugation, the BV2 cell membranes were collected.

### 4.4 MNPs@PLX synthesis

First, 4 mg of PLX3397 and 4 mg PLGA were dissolved in 10 mL CHCl_3_, and 100 µL of the mixture was sonicated for 5 min to form a nanoparticle core (NPs@PLX). Then, the pre‐extracted BV2 cell membranes, 100 µL of DSPE‐PEG2000 (10 mg mL^−1^) lipid dispersion, and NPs@PLX were mixed and extruded through a 220 nm polycarbonate membrane for approximately 10 passes to form MNPs@PLX. Finally, the nanoparticles were dialyzed with PBS (pH 7.4) for 24 h at room temperature.

### 4.5 Characterization of MNPs@PLX

The nanoparticles size distribution, PDI, and ζ‐potential of NPs@PLX and MNPs@PLX were investigated by Zetasizer Nano ZS (Malvern, U.K.). NPs@PLX and MNPs@PLX morphologies were examined by transmission electron microscopy (FEI Tecnai G2 F20 S‐Twin, USA).

### 4.6 Encapsulation efficiency and drug loading

Free PLX, NPs@PLX, and MNPs@PLX were separated by ultracentrifugation (Millipore, Billerica, MA, USA). The content of the initial concentration of PLX3397 in free PLX, NPs@PLX, and MNPs@PLX was determined by UV–vis spectrometry (Evolution220, Thermo Fisher Scientific, Waltham, MA, USA).

The encapsulation efficiency (EE) was calculated as follows:

EE%=(Wt−Wf)/Wt×100%

where *Wt* refers to the initial concentration of the PLX3397 in NPs@PLX or MNPs@PLX, and *Wf* refers to the free PLX in the filtrate.

The loading efficiency (LE) was calculated as follows:

LE%=WF/WN×100%

where *WF* refers to the weight of the PLX3397 in NPs@PLX and MNPs@PLX, and *WN* refers to the weight of NPs@PLX or MNPs@PLX.

### 4.7 Drug release profile of MNPs@PLX

Dialysis method was used to measure the release kinetics of PLX3397 from NPs@PLX and MNPs@PLX. Briefly, 1 mL NPs@PLX and MNPs@PLX (containing 2 mg mL^−1^ PLX3397) were suspended, and then the solutions were dialyzed at 37°C. Ultimately, PBS was collected at different time points (1–72 h) and the resulting PLX3397 was measured.

### 4.8 Flow cytometry

Flow cytometry was performed according to our recent work.^[^ ^42^ ^]^ Briefly, BV2 cells were incubated with fresh medium containing NPs@PLX or MNPs@PLX (20 µg mL^−1^ PLX, 0.2 µg mL^−1^ Cy5.5). After 3 h incubation, the cells were collected for flow cytometry analysis (Ex: 638 nm, Em: 712/25 nm) (Beckman Cytoflex, Indianapolis, IN, USA).

### 4.9 In vitro BBB permeability evaluation of MNPs@PLX

Nearly 1 × 10^5^ bEnd.3 cells were seeded in polyester transwell insert (6 wells, pore diameter of 0.4 µm, 4.67 cm^2^) (Corning, NY, USA) for 7 days. The Millicell‐ERS volt‐ohmmeter was then used to detect the trans‐epithelial electrical resistance of bEnd.3 monolayer cells as described.^[^ ^43^ ^]^ To evaluate the capacity of MNPs@PLX‐Cy5.5 to penetrate BBB, the culture medium in the apical chamber was changed for PBS, NPs@PLX‐Cy5.5, and MNPs@PLX‐Cy5.5 (containing 0.5 µg mL^−1^ Cy5.5 in 0.7 mL). Four hours later, the medium in the lower chamber (basolateral chamber) was replaced with fresh DMEM. The fluorescence signal of insert of the transwell and the basolateral chamber of the bEnd.3 layer was imaged using IVIS Spectrum System (Caliper, MA, Boston, USA).

### 4.10 In vivo and ex vivo imaging and biodistribution analysis

In vivo imaging and ex vivo biodistribution analysis was performed according to our previous study.^[^ ^42^ ^]^ Mice were injected with NPs@PLX‐Cy5.5 and MNPs@PLX‐Cy5.5 (200 µL, containing 2 mg mL^−1^ PLX3397 and 20 µg mL^−1^ Cy5.5) via the tail vein. The in vivo fluorescence signals were obtained and semiquantitative analysis using IVIS Spectrum System (Caliper, MA, Boston, USA) (Ex: 674 nm; Em: 692 nm). After injection, mice were euthanized, and the major organs (heart, liver, spleen, lung, kidney, and brain) were collected for semiquantitative biodistribution analysis using IVIS Spectrum System (Caliper, MA, Boston, USA) (Ex: 674 nm; Em: 692 nm).

### 4.11 Animals and drug treatment

All animal experiments were approved by the Institutional Animal Care and Use Committee at Guangzhou Medical University (Approval number: GY2020‐041). Eight‐week‐old C57BL/6 male and female mice were purchased from SPF Biotechnology Co., Ltd. (Beijing, China). Three to four mice were maintained in each cage with 12 h of light and 12 h of darkness, ambient feeding temperature (22 ± 1°C) and relative humidity (60 ± 5%), and continuous access to food and water. All experiments were conducted according to the National Institute of Health guidelines on the care and use of animals (NIH Publications No. 8023, revised 1978).

To detect the effects of *Cntnap4* deficiency on memory processing, AAV‐Cntnap4 shRNA‐injected mice and heterozygous (Cntnap4^+/−^) mice were used in this study. Male and female mice were injected with AAV‐Cntnap4 shRNA for 4 weeks, and then behavioral tests were performed. *Cntnap4* knockout mice mating with WT C57BL/6J mice to generate Cntnap4^+/−^ mice, as described in our previous study and were generated by the Shanghai Model Organisms Center, Inc. (Shanghai, China).^[^ ^8^ ^]^

To test the effect of estrogen on the inflammatory response, female mice were injected with AAV‐Cntnap4 shRNA. Four weeks later, mice were intraperitoneally injected with estrogen at 10 µg kg^−1^ day^−1^, 5 days/week, for 3 weeks as described previously.^[^ ^44^ ^]^ Then the immunofluorescence experiment was performed.

To examine the anti‐inflammatory effects of GABA and clorgyline, Cntnap4^+/−^ mice were injected intraperitoneally with GABA (1 or 5 mg kg^−1^) or clorgyline (10 mg kg^−1^) every other day for 4 weeks. Then behavioral tests, electrophysiological and biological experiments were performed.

To explore the effects of MNPs@PLX in treating Cntnap4^+/−^ mice, mice were administered intravenously with equivalent molecular doses of NPs@PLX, MNPs@PLX, MNPs, NPs@PLX, and MNPs@PLX containing 2 mg mL^−1^ PLX3397 or the control vehicle for 12 continuous days. Then behavioral tests, electrophysiological and biological experiments were performed.

### 4.12 Stereotaxic injection of *Cntnap4* AAV‐shRNA in hippocampus

The shRNA sequence targeting *Cntnap4* was used as we reported previously,^[^ ^8^ ^]^ and AAV2/9‐CAG‐EGFP‐U6 was used as the vector. The stereotaxic injection was performed as described previously,^[^ ^8^ ^]^ and the target site was set as Bregma AP, −2.0 mm, ML, ± 2.0 mm, DV, −2.0 mm. To examine the sex‐specific deficiency in hippocampus, Cntnap4 AAV‐shRNA was injected in male and female mice.

### 4.13 Open field test

The open field test was performed according to our previous work.^[^ ^45^ ^]^ Briefly, the open field consisted of a square arena (40 cm × 40 cm) with a white floor and 40 cm high walls. The field was subdivided into peripheral and central sectors, where the central sector was 20 cm × 20 cm and the peripheral sector encompassed the remaining squares. Mouse was allowed to explore for 15 min, and then the behavioral parameters were recorded using a video tracking system (EthoVisione XT software; Beijing, China). The apparatus was thoroughly cleaned with diluted 75% ethanol between each trial.

### 4.14 Y **maze** test

Y maze test was performed as mentioned previously.^[^ ^46^ ^]^ Briefly, mice were placed in the center zone and allowed to explore three identical arms of the Y maze for 8 min. Spontaneous alternations were defined and collected using EthoVision XT (Beijing, China). The apparatus was thoroughly cleaned with diluted 75% ethanol between each trial.

### 4.15 Morris **water maze** test

The Morris Water Maze test was performed according to our previous work.^[^ ^46^ ^]^ Over five consecutive training days, mice were placed in one of the four quadrants randomly, and the training was performed with four trials per day. In each trial, the mouse swam until it found the hidden platform and stayed on the platform for 15 s. The mouse was guided to the platform and allowed to stay on the platform for 15 s, if it could not find the platform within 60 s. On day 6, the mouse was placed in the pool without the hidden platform, and it was allowed to swim for 60 s. Swimming paths were obtained and analyzed automatically using Smart 3.0 video tracking software (Panlab, Barcelona, Spain).

### 4.16 RNA‐sequencing (RNA‐seq) and bioinformatic analysis

RNA‐seq was performed according to our previous study.^[^ ^42^ ^]^ Briefly, total RNA was isolated from the hippocampus using Trizol (Life Technologies, Carlsbad, CA, USA), and RNA libraries were obtained using the NEBNext® UltraTM RNA Library Prep Kit for Illumina® (NEB, Ipswich, MA, USA). The sequencing was performed on the HiSeq 1000 platform (Illumina). Bioinformatic analysis was performed using R package edgeR, where KEGG and GO analysis proceeded for pathway analysis.

### 4.17 Quantitative **real**‐**time** (RT)‐PCR

Total RNA was extracted from cells or mouse brains using Trizol (Life Technologies, Carlsbad, CA, USA). RNA was then converted to cDNA using a Reverse Transcription Kit (QIAGEN, Waltham, MA, USA), and then the relative gene expression levels were detected. GAPDH mRNA levels were used to normalize the data. The 2^−ΔΔCT^ method was used to analyze the results as described previously.^[^ ^42^ ^]^ Data are from three separate experiments, each of which was performed in triplicate. Primers are listed in Table S2, Supporting Information.

### 4.18 Western blotting

Mouse hippocampus samples or cells were homogenized in RIPA buffer with 1 mM PMSF (Beyotime, Shanghai, China) to obtain the supernatants. Protein concentrations were determined using a BCA kit (Beyotime, Shanghai, China). Protein was separated on 10% SDS‐PAGE gels, and transferred to polyvinylidene difluoride membranes. The membranes were probed with primary antibodies, and then the HRP‐conjugated secondary antibodies. Bands were visualized by chemiluminescence (ECL, Beyotime, Shanghai, China). Band intensities were observed on the GeneGnome XRQ Chemiluminescence imaging system (Gene Company, Hong Kong, China), and analyzed using Image J software.

### 4.19 Immunofluorescence and image analysis

The brains were fixed in 4% paraformaldehyde and dehydrated in 20−30% sucrose solution. Embedded brains were sectioned into slices at a thickness of 30 mm, and brain sections were then blocked with 5% BSA. After incubation with primary antibody and fluorescent‐labeled secondary antibody, images were obtained by confocal microscopy (SP8; Leica, Hamburg, Germany). Images were quantified using the Image‐Pro Plus 6.0 photogram analysis system (IPP 6.0, Media Cybernetics, Bethesda, MD, USA).

### 4.20 Golgi staining

Golgi staining was used to acquire the morphology of neuronal dendrites and dendritic spines using the FD Rapid Golgi Stain Kit (FD Neuro Technologies, Columbia, MD, USA). Briefly, mice were anesthetized and brain tissues were impregnated in mixed solutions of A and B buffer for 2 weeks. The brains were transferred to solution C for 72 h and then sliced at a thickness of 200 µm with a freezing microtome (Leica, Hamburg, Germany). The sections were then stained according to the manufacturer's instructions. Images were obtained using microscope (Leica CS2, Hamburg, Germany), and neuronal dendrites and dendritic spines were analyzed by Image J software.

### 4.21 Enzyme‐linked immunosorbent assay (ELISA)

Cytokine expressions in hippocampus, cell samples, and supernatants were evaluated using ELISA kits (Shanghai Enzyme‐linked Biotechnology Co., Ltd., Shanghai, China), and the detailed procedure was described in our recent study.^[^ ^42^ ^]^ Protein concentrations were expressed as pg mL^−1^ or pg ng^−1^ per mg protein (pg mg^−1^ protein).

### 4.22 Metabolomics analysis

Hippocampal metabolites were analyzed as we described previously.^[^ ^47^ ^]^ LC‐MS analysis was performed on an Ultimate 3000 UHPLC (Thermo Fisher Scientific, Waltham, MA, USA) coupled with a Synapt G2‐Si QToF mass spectrometer (Thermo Fisher Scientific, Waltham, MA, USA), and the data were obtained using MassLynx 4.2 (Thermo Fisher Scientific, Waltham, MA, USA). The sample sequence and data pre‐processing were performed using Compound Discoverer 3.1 software (CD3.1, Thermo Fisher Scientific, Waltham, MA, USA).

### 4.23 Brain‐slice preparation and electrophysiological recording

Hippocampal slices (300 µm) were cut in ice‐cold modified artificial cerebrospinal fluid (ACSF) and then incubated in ACSF. Whole‐cell patch‐clamp recordings were performed according to our previous study,^[^ ^45^ ^]^ and they were performed with a MultiClamp 700B amplifier and 1, 440A digitizer (Molecular Device). Both spontaneous inhibitory post‐synaptic currents (sIPSCs) and evoked inhibitory post‐synaptic currents (eIPSCs) were recorded at a holding potential of −70 mV. For sIPSC and eIPSC recording, glass pipettes were filled with the solution containing (in mM): CsCl (140), HEPES (10), EGTA (0.2), MgCl_2_ (1), MgATP (4), NaGTP (0.3), sodium phosphocreatine (10), QX‐314 (5) at PH 7.3 and 290 mOsm. The resistance of pipettes was 3−5 MΩ. 20 µM CNQX and 50 µM DL‐AP5 was used to block AMPA and NMDA receptors‐mediated currents. Under recording, eIPSCs were generated with a two‐concentric bipolar stimulating electrode (FHC, Inc.). To detect the excitability of CA1 neurons, APs were recorded under the current‐clamp mode by injecting a series of gradually increased depolarizing pulses starting from 0 to 180 pA at a step of 20 pA, with the pipette solution including (in mM): 125 Glu‐K, 5 KCl, 10 HEPES, 0.2 EGTA, 1 MgCl_2_, 4 MgATP, 0.3 NaGTP, 10 Na_2_phosphocreatine (pH 7.40, 285 mOsm). The resting membrane potential and membrane input resistance were calculated in response to a series of hyperpolarizing pulses (0 pA, −25 pA, −50 pA, −75 pA, −100 pA). Hippocampal LTP was recorded as we described previously.^[^ ^48^ ^]^ fEPSPs were evoked in the CA1 stratum radiatum using an Axon instrument with a MultiClamp 700B amplifier (Molecular Devices). LTP induction was performed by two trains of 100 pulses in 1 s, at an interval of 20 s, and its level was evaluated at an average of 50−60 min after tetanus stimulation.

### 4.24 Statistical analysis

Statistical analysis was performed using Prism 9.0 (GraphPad Software, La Jolla CA). The Student's *t*‐test was used for comparisons between two groups, and one‐way analysis of variance (ANOVA) followed by the Tukey's *post‐hoc* test or two‐way ANOVA followed by the Bonferroni *post‐hoc* test was used for multiple comparisons. Data are presented as the mean ± standard error of the mean (SEM), and the statistical significance level was set at *p* < 0.05.

## AUTHOR CONTRIBUTIONS

Yunlong Zhang designed the research. Wenlong Zhang and Mengran Zhang performed Western blotting. Huaqing Chen prepared and provided the biomimetic MNPs@PLX system. Liuyan Ding and Runfang Ma carried out immunostaining assays. Junwei Gong and Shaohui Zheng injected AAVs and performed behavioral tests. Jie Huang and Yan Liu performed electrophysiological experiments. Yunlong Zhang and Wenlong Zhang analyzed the data. Yunlong Zhang wrote the manuscript. Juan C. Piña‐Crespo edited the manuscript. All authors read and commented on it.

## CONFLICT OF INTEREST STATEMENT

The authors declare no conflicts of interest.

## Supporting information

Supporting Information

Supporting Information

## Data Availability

The data used to support the findings of this study are available from the corresponding author upon request.
