# Supplementary material for: Microglial targeted therapy relieves cognitive impairment caused by Cntnap4 deficiency
Source: Exploration (Beijing). 2023 May 10;3(3):20220160. doi: 10.1002/EXP.20220160 (PMC10624376; doi:10.1002/EXP.20220160)
Supplement: Supplementary file 1 — Supporting Information [file EXP2-3-20220160-s001.pdf]

Supporting Information

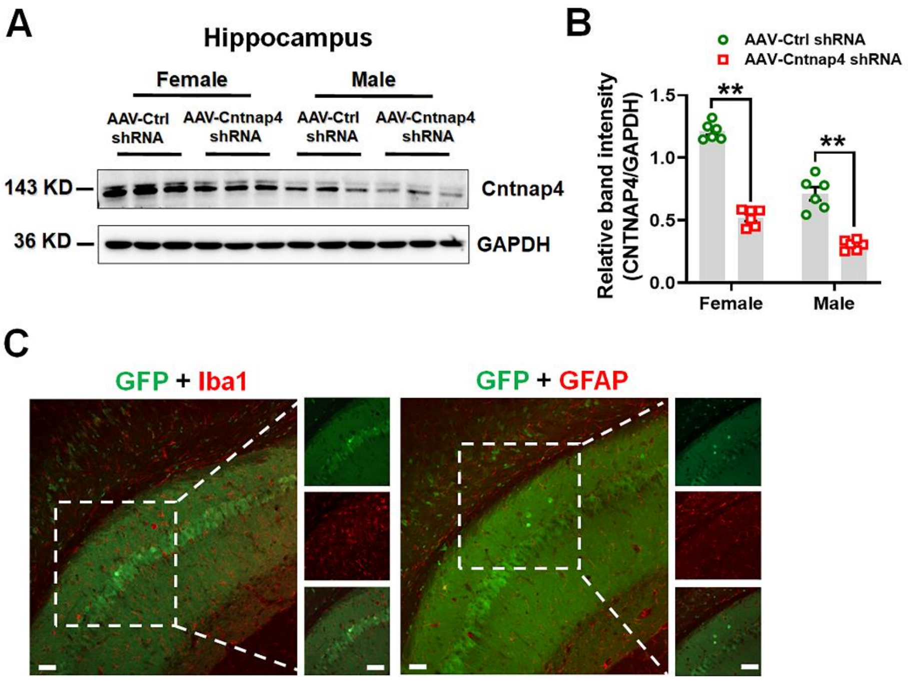

Figure S1. Expression of Cntnap4 after virus injection. (A and B) Representative blots and quantification showing Cntnap4 expression in hippocampi of male and female mice after injection with AAV-Ctrl shRNA or AAV-Cntnap4 shRNA. n = 6 per group. (C) Immunofluorescence staining of GFP with Iba1 and GFAP in the hippocampus. Scale bar in left panel is 50 μm, and in right panel is 25 μm. Results are expressed as the mean ± SEM. $^{**}p < 0.01$ vs. AAV-Ctrl shRNA group. Statistical significance was determined by two-way ANOVA and Bonferroni tests for *post hoc* comparisons.

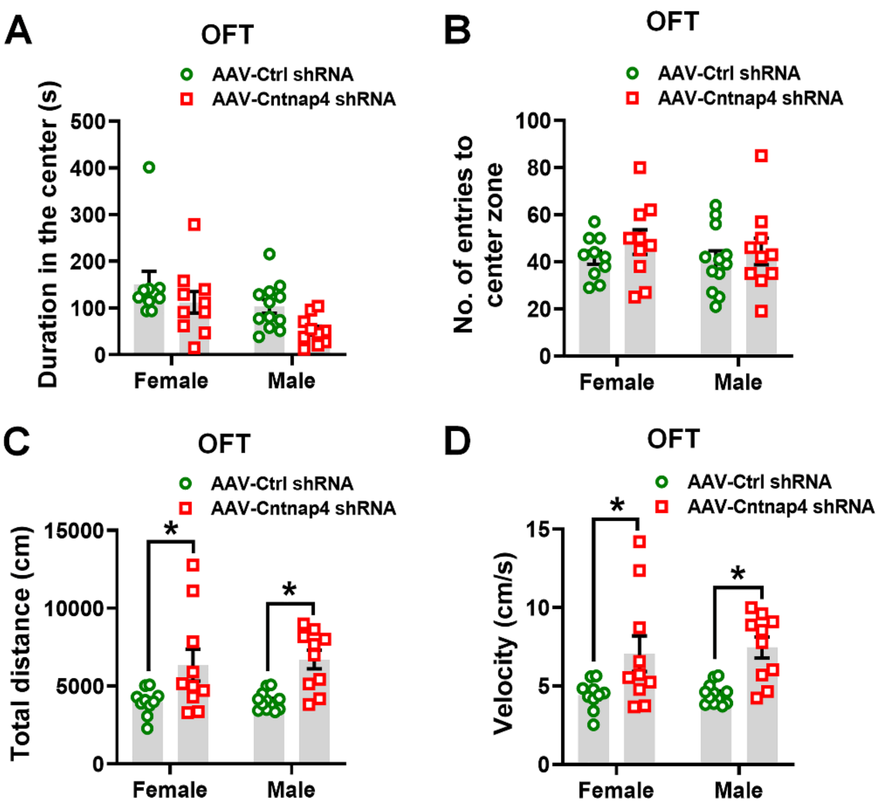

Figure S2. Effect of *Cntnap4* knockdown on the locomotor activity of mice in the open field test. The time spent in the center zone of the open field (A), number of entries to the center zone (B), total travelled distance (C), and movement speed (D) upon *Cntnap4* knockdown in the hippocampus was examined by the open field test. n = 10 in the AAV-Ctrl shRNA—Female, AAV-Cntnap4 shRNA—Female, and AAV-Cntnap4 shRNA—Male groups, n = 12 in the AAV-Ctrl shRNA—Male group. Results are expressed as the mean ± SEM. $^{*}p < 0.05$ vs. AAV-Ctrl shRNA group. Statistical significance was determined by two-way ANOVA and Bonferroni tests for *post hoc* comparisons.

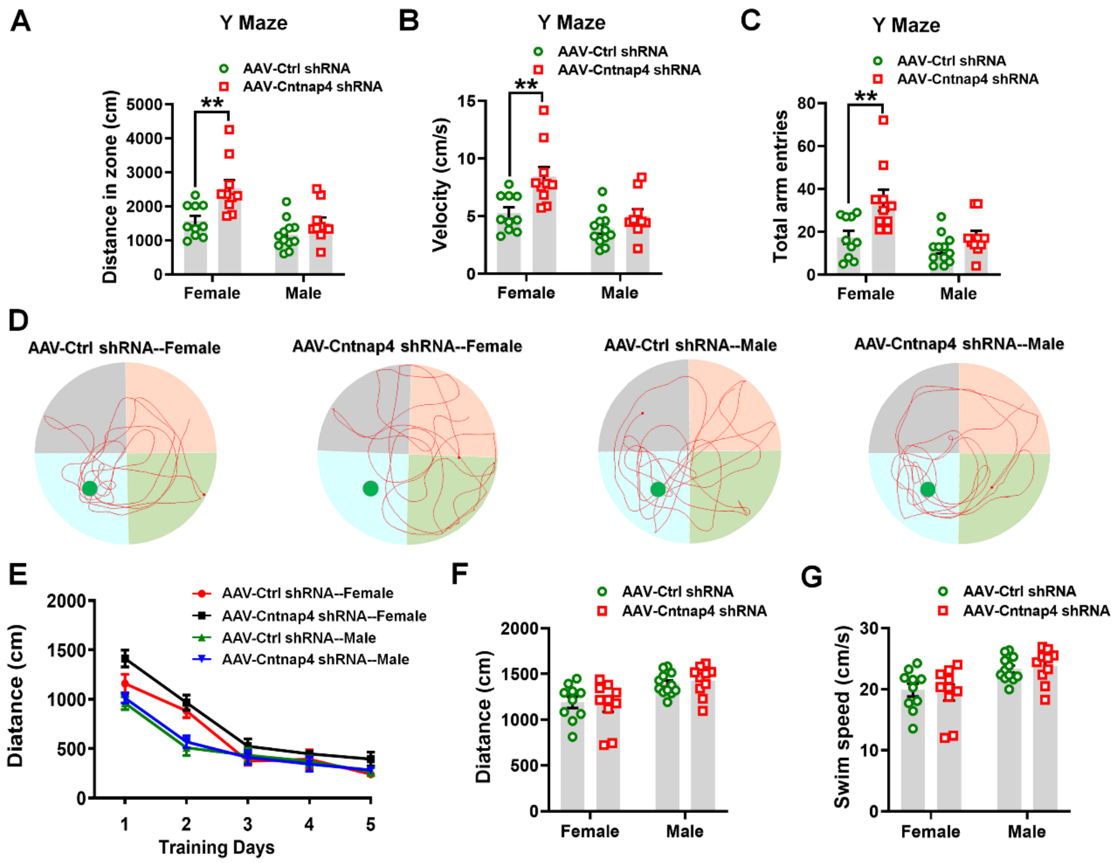

Figure S3. Effect of *Cntnap4* knockdown on the behavioral performance in the Y maze and water maze tests. Total travelled distance (A), movement speed (B) and total arm entries (C) in the Y maze. (D) Representative swimming traces of mice in the water maze. Total swimming distance in the five-day training course (E), and probe tests (F). Swimming speed (G) in the probe test. n = 10 in the AAV-Ctrl shRNA—Female, AAV-Cntnap4 shRNA—Female, and AAV-Cntnap4 shRNA—Male groups, n = 12 in the AAV-Ctrl shRNA—Male group. Results are expressed as the mean ± SEM. $^{**}p < 0.01$ vs. AAV-Ctrl shRNA group. Statistical significance was determined by two-way ANOVA and Bonferroni tests for *post hoc* comparisons.

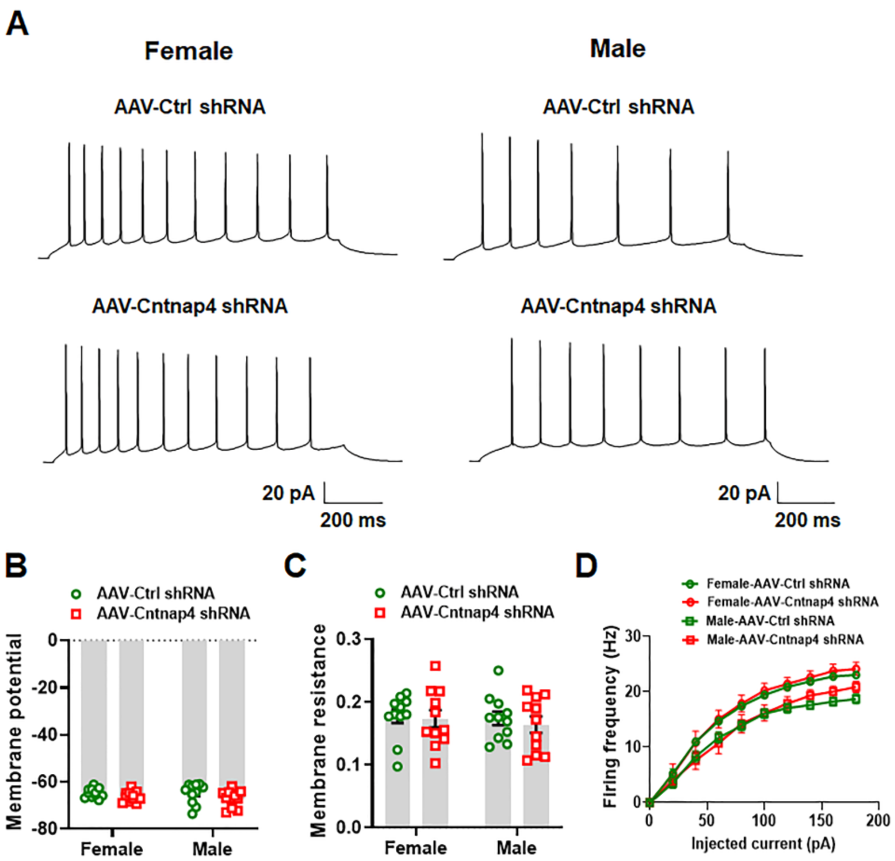

Figure S4. Effect of Cntnap4 knockdown on the action potentials. (A) Representative traces of AP firing. (B-D) The membrane potential, membrane resistance and firing frequency were recorded from CA1 neurons. n = 11 per group. Results are expressed as the mean ± SEM. Statistical significance was determined by two-way ANOVA and Bonferroni tests for *post hoc* comparisons.

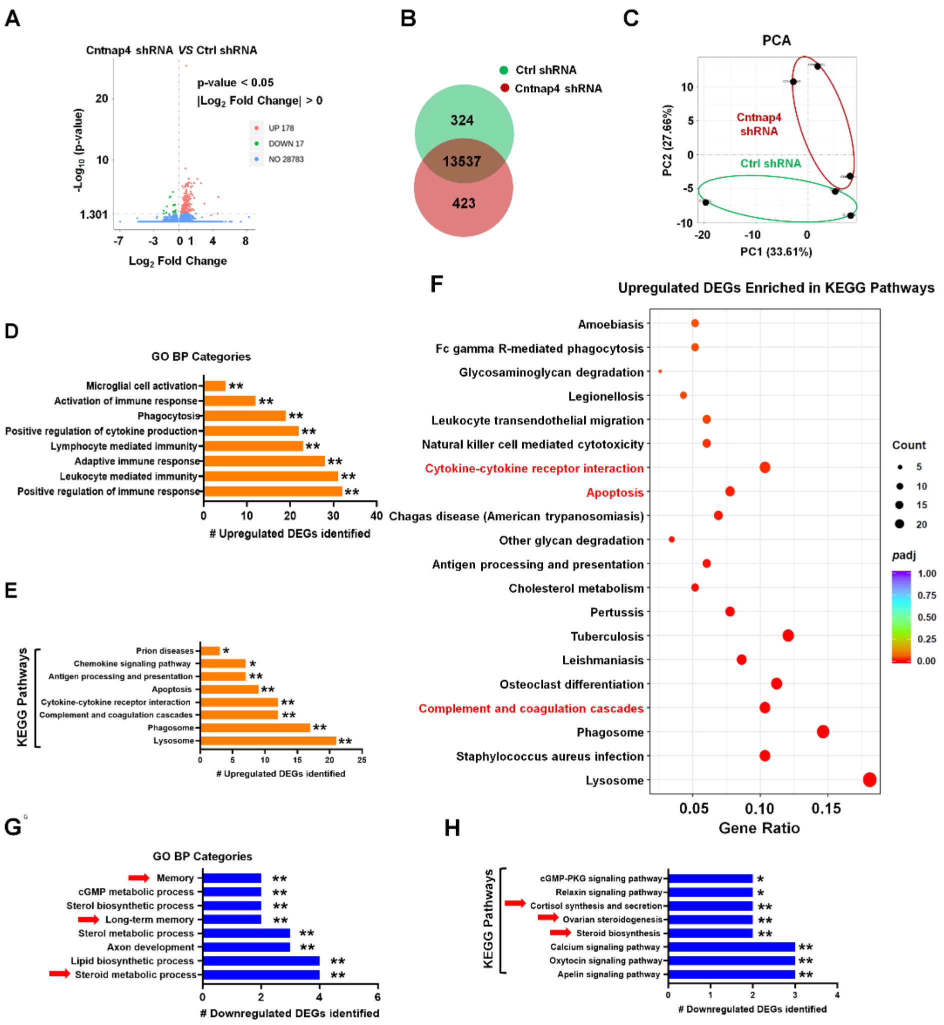

Figure S5. Hippocampal RNA-seq profiles in female *Cntnap4* knockdown mice. (A and B) Volcano plot and Venn diagram showing the DEGs between AAV-Ctrl shRNA and AAV-Cntnap4 shRNA in female mice. (C) PCA plot of RNAs expressed in the AAV-Ctrl shRNA and AAV-Cntnap4 shRNA groups. (D) GO pathways enriched by upregulated DEGs between the AAV-Ctrl shRNA and AAV-Cntnap4 shRNA groups. (E and F) KEGG pathways enriched by upregulated DEGs in hippocampi of AAV-Cntnap4 shRNA versus AAV-Ctrl shRNA. (G) GO pathways enriched by downregulated DEGs between the AAV-Ctrl shRNA and AAV-Cntnap4 shRNA groups. (H) KEGG pathways enriched by downregulated DEGs in the AAV-Cntnap4 shRNA versus AAV-Ctrl shRNA groups.

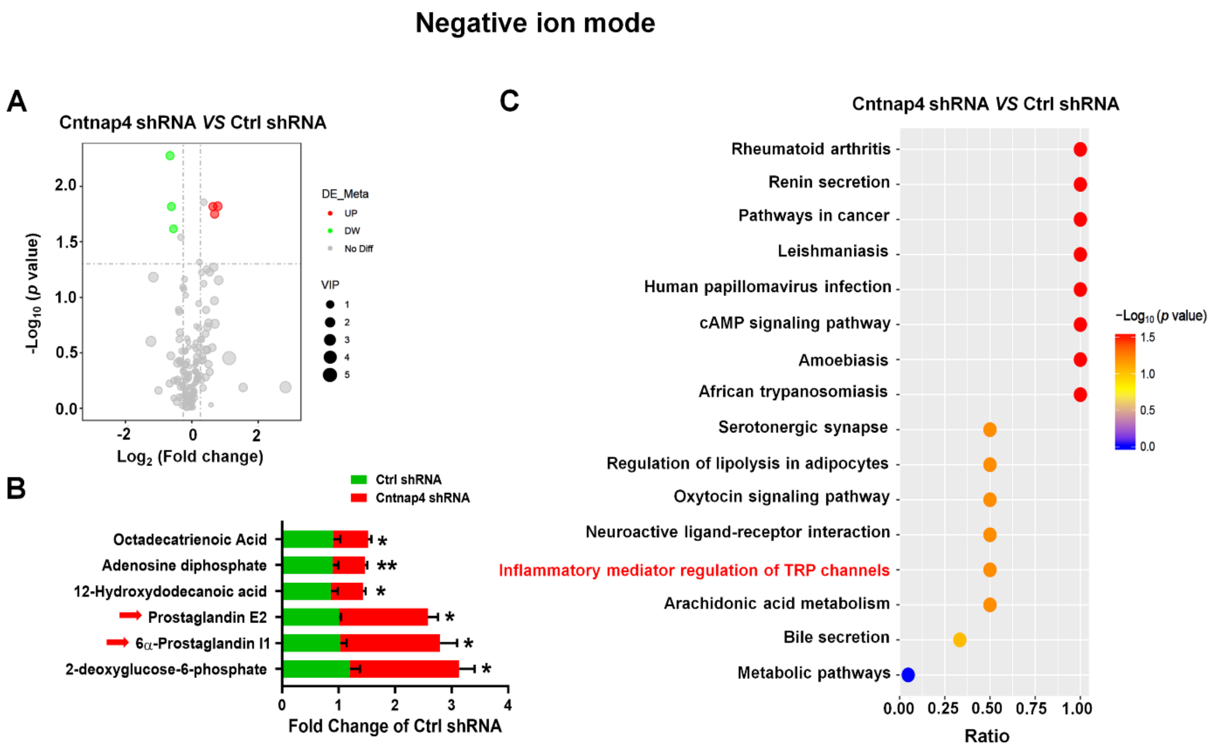

Figure S6. Hippocampal metabolomic analysis of differentially regulated metabolites in female *Cntnap4* knockdown mice in the negative ion mode. (A) Volcano plot showing upregulated and downregulated differential metabolites in hippocampi of AAV-Cntnap4 shRNA versus AAV-Ctrl shRNA mice. (B) The upregulated and downregulated differential metabolites in hippocampi of AAV-Cntnap4 shRNA versus AAV-Ctrl shRNA mice. (C) Altered KEGG pathways enriched by the differential metabolites.

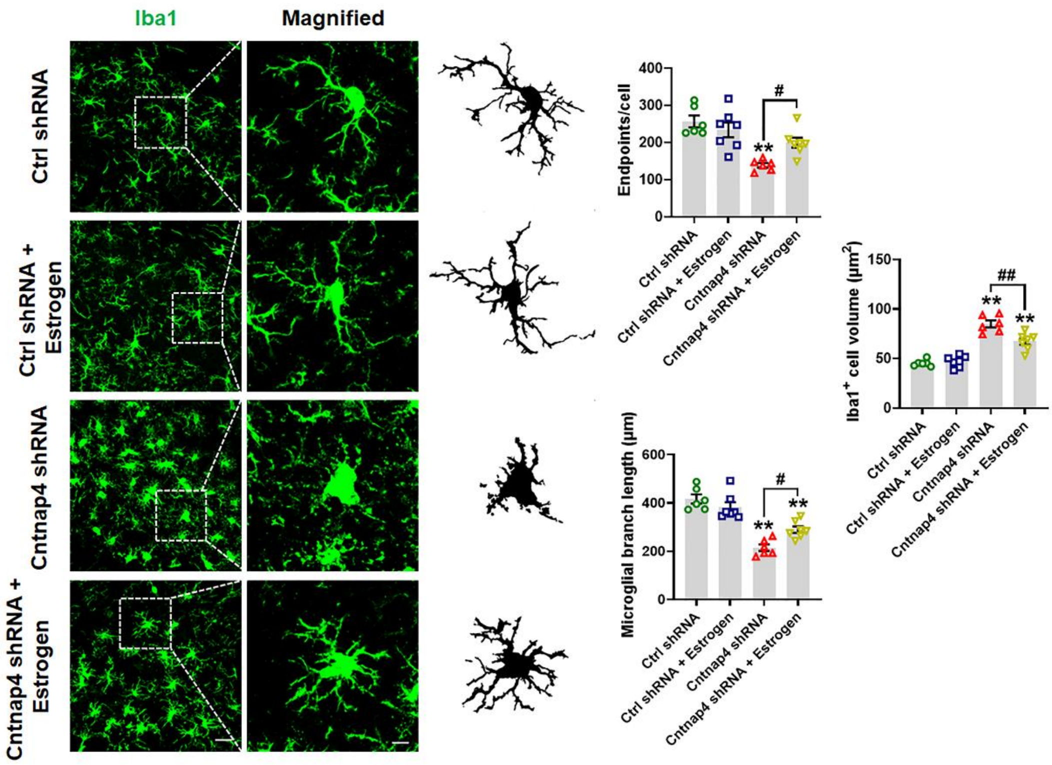

Figure S7. Effect of estrogen on the microglial activation in female *Cntnap4* knockdown mice. Immunofluorescence staining and quantification of endpoint voxels, branch length and volume of Iba1-positive cells in hippocampi of mice injected with AAV-Ctrl shRNA or AAV-Cntnap4 shRNA. Scale bars, 20 μm. Magnified images are shown in the right column. Scale bars, 6 μm. n = 6–7. Results are expressed as the mean ± SEM. $^{**}p < 0.01$ vs. Ctrl shRNA; $^{\#\#}p < 0.01$, $^{\#}p < 0.05$ vs. Cntnap4 shRNA. Statistical significance was determined using one-way ANOVA and Tukey's tests for *post hoc* comparisons.

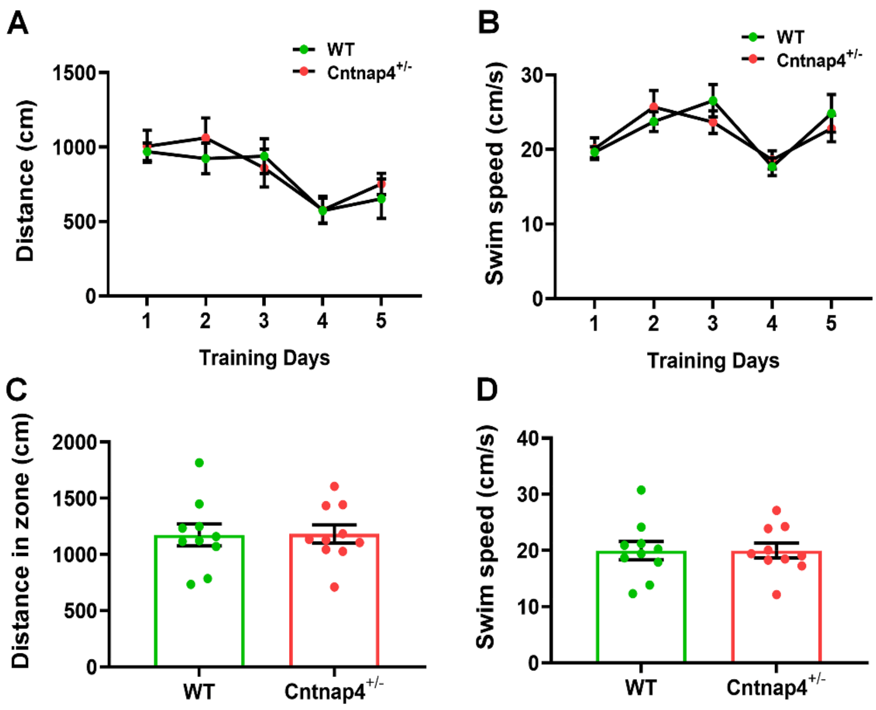

Figure S8. Swimming speed of female WT and Cntnap4$^{+/-}$ mice in the water maze. Total swimming distance (A) and swimming speed (B) of WT and Cntnap4$^{+/-}$ mice over a five-day training course. Total swimming distance (C) and swimming speed (D) of WT and Cntnap4$^{+/-}$ mice in the probe test. Results are expressed as the mean ± SEM. Statistical significance was determined using the Student's *t*-test.

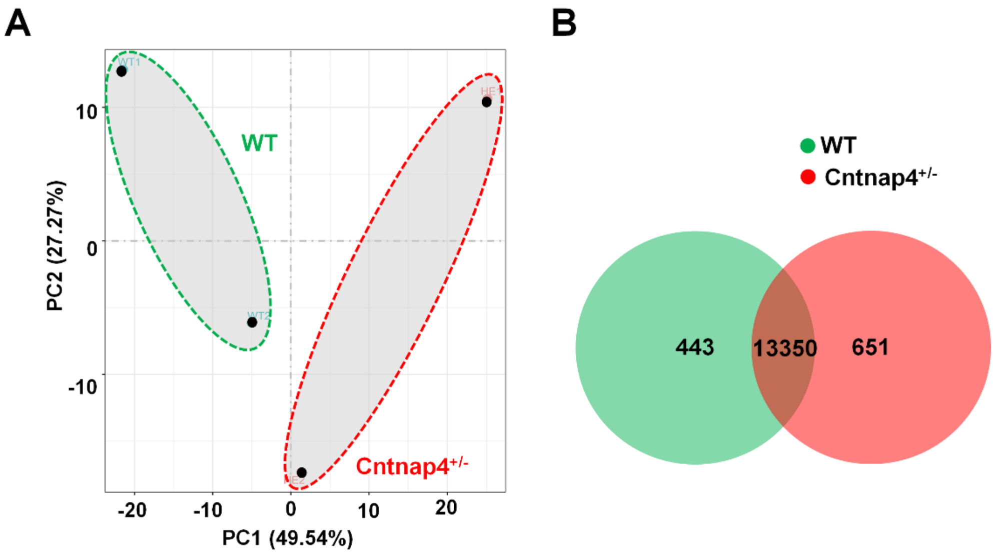

Figure S9. Hippocampal RNA-seq profiles in Cntnap4$^{+/-}$ mice. (A) PCA plot of RNAs from WT and Cntnap4$^{+/-}$ mice. (B) Venn diagram of DEGs between WT and Cntnap4$^{+/-}$ mice.

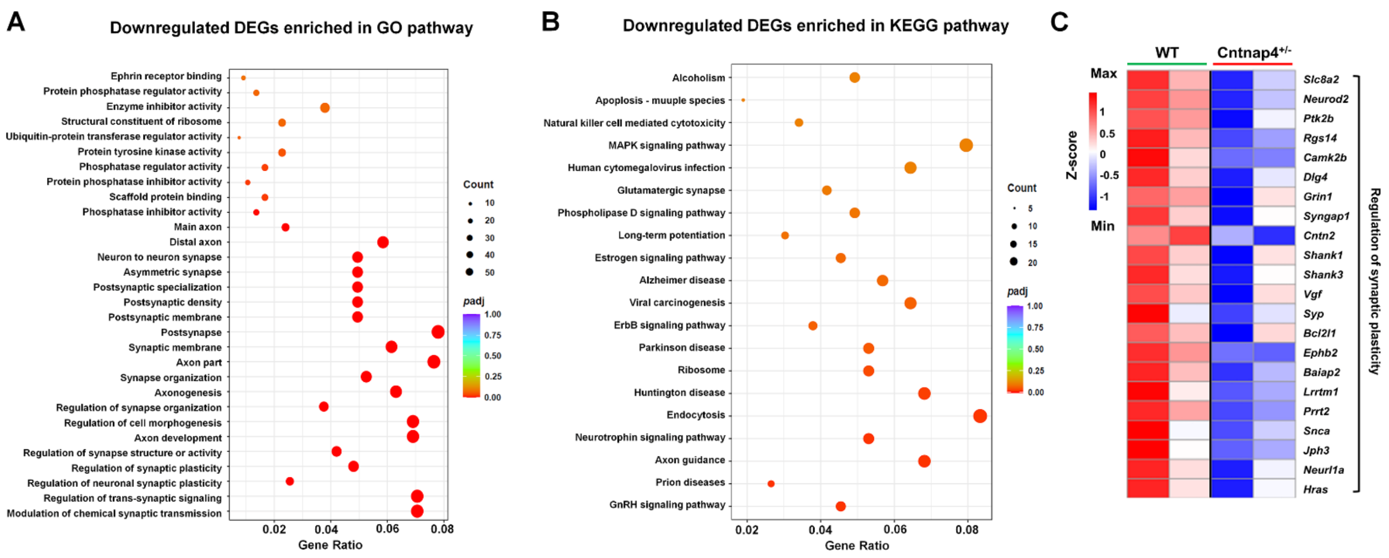

Figure S10. Signaling pathways enriched by downregulated DEGs in Cntnap4$^{+/-}$ mice. GO pathways (A) and KEGG pathways (B) enriched by downregulated DEGs in Cntnap4$^{+/-}$ versus WT mice. (C) Hierarchical clustering of DEGs enriched in the "Regulation of synaptic plasticity" GO pathways between WT and Cntnap4$^{+/-}$ mice.

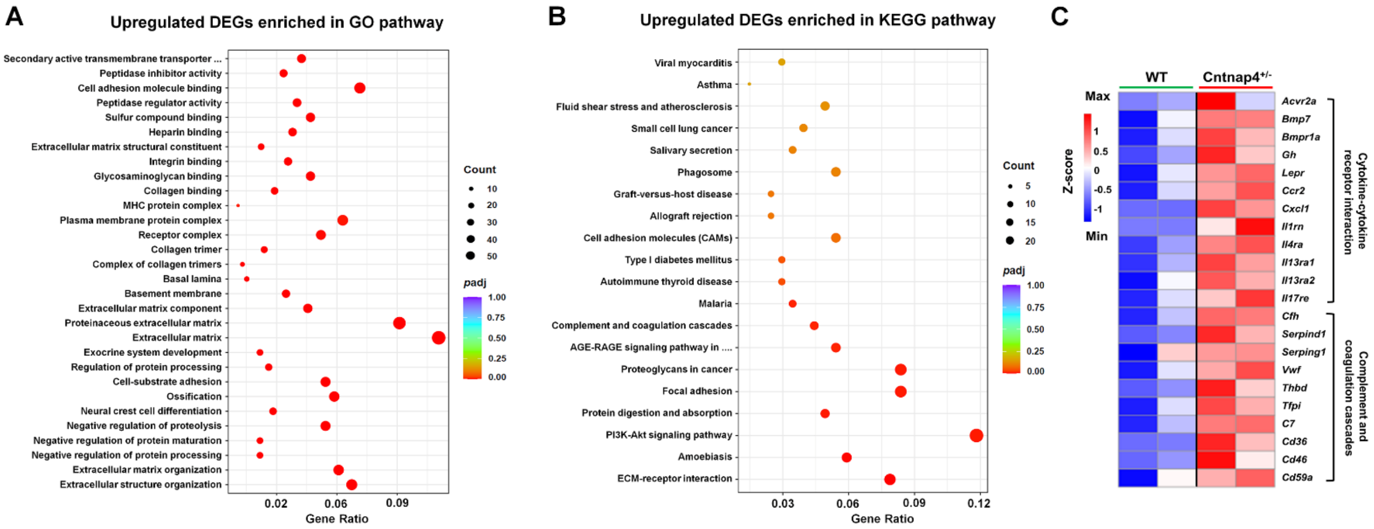

Figure S11. Signaling pathways enriched by upregulated DEGs in Cntnap4$^{+/-}$ mice. GO pathways (A) and KEGG pathways (B) enriched by upregulated DEGs in Cntnap4$^{+/-}$ versus WT mice. (C) Hierarchical clustering of DEGs enriched in the "Cytokine-cytokine receptor interaction" and "Complement and coagulation cascades" KEGG pathways between WT and Cntnap4$^{+/-}$ mice.

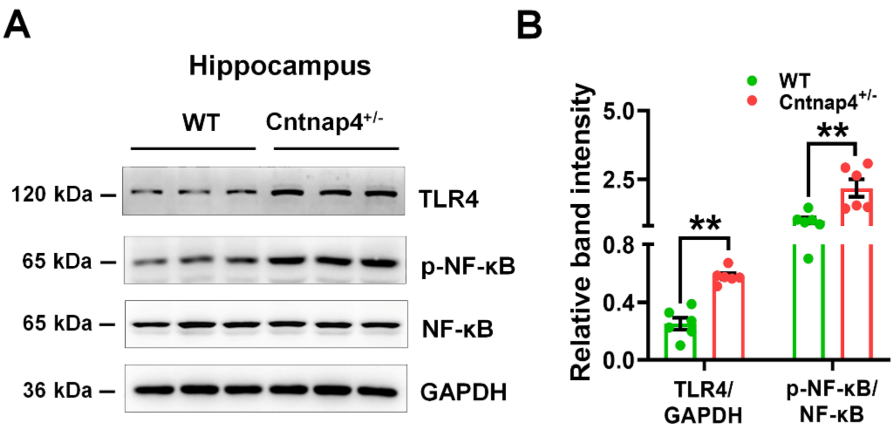

Figure S12. Expression of TLR4/NF-κB pathway in Cntnap4$^{+/-}$ mice. (A and B) Representative blots and quantification showing TLR4, p-NF-κB, and NF-κB expression in hippocampi of WT and Cntnap4$^{+/-}$ mice. n = 6 per group. Results are expressed as the mean ± SEM. $^{**}p < 0.01$ vs. WT. Statistical significance was determined using Student's *t*-test.

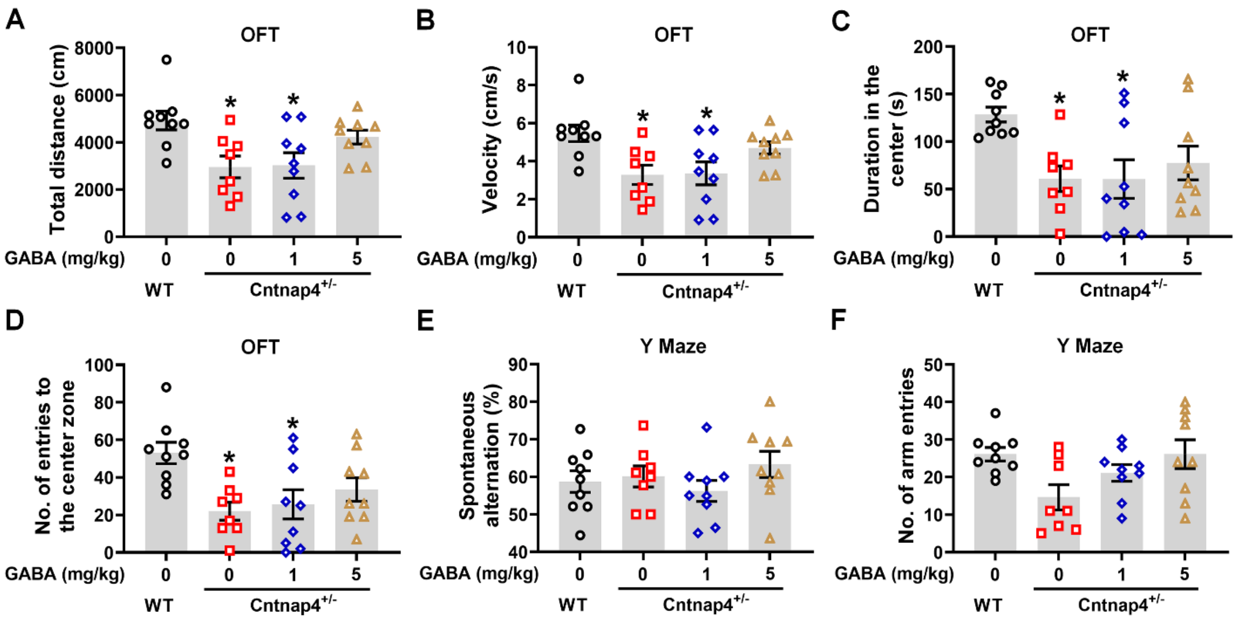

Figure S13. Behavioral performance after GABA supplementation in Cntnap4$^{+/-}$ mice in the open field and Y maze tests. The total travelled distance (A), movement speed (B), time spent in the center zone of the open field (C), and number of entries to the center zone (D) of Cntnap4$^{+/-}$ mice treated with 1 and 5 mg/kg GABA. The spontaneous alterations (E) and number of arm entries in the Y maze for Cntnap4$^{+/-}$ mice treated with 1 and 5 mg/kg GABA (F). Results are expressed as the mean ± SEM. $^{*}p < 0.05$ vs. WT. Statistical significance was determined using one-way ANOVA and Tukey's tests for *post hoc* comparisons.

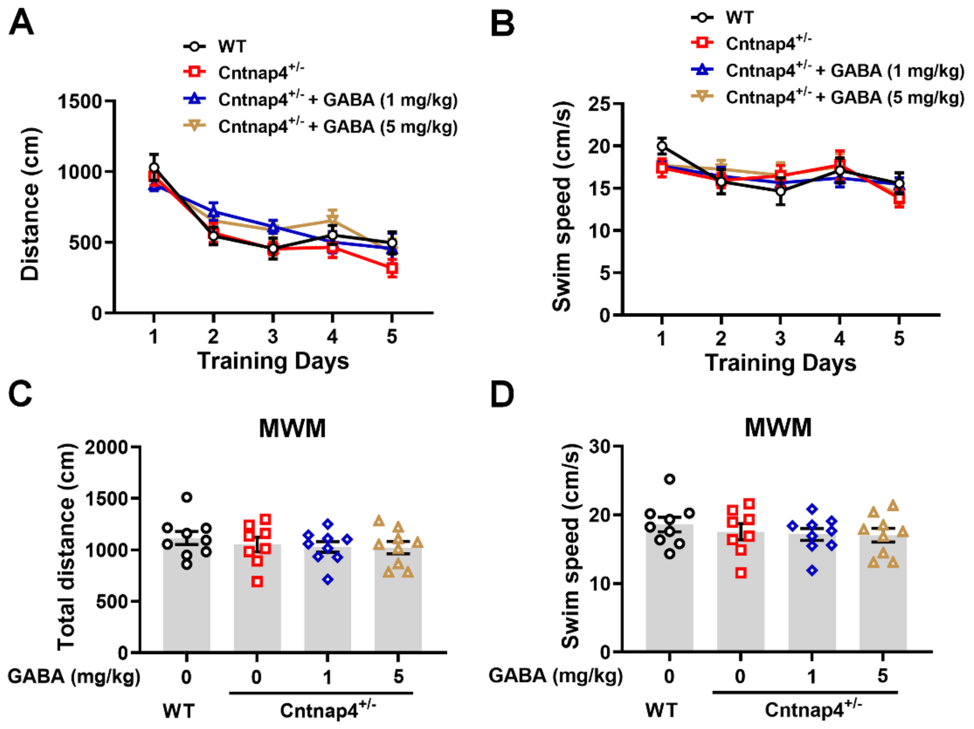

Figure S14. Swimming speed after GABA supplementation in Cntnap4$^{+/-}$ mice in the water maze test. Total swimming distance (A) and swimming speed (B) of Cntnap4$^{+/-}$ mice treated with 1 and 5 mg/kg GABA over a five-day training course. Total swimming distance (C) and swimming speed (D) of Cntnap4$^{+/-}$ mice treated with 1 and 5 mg/kg GABA in the probe tests. Results are expressed as the mean ± SEM. Statistical significance was determined using one-way ANOVA and Tukey’s tests for *post hoc* comparisons.

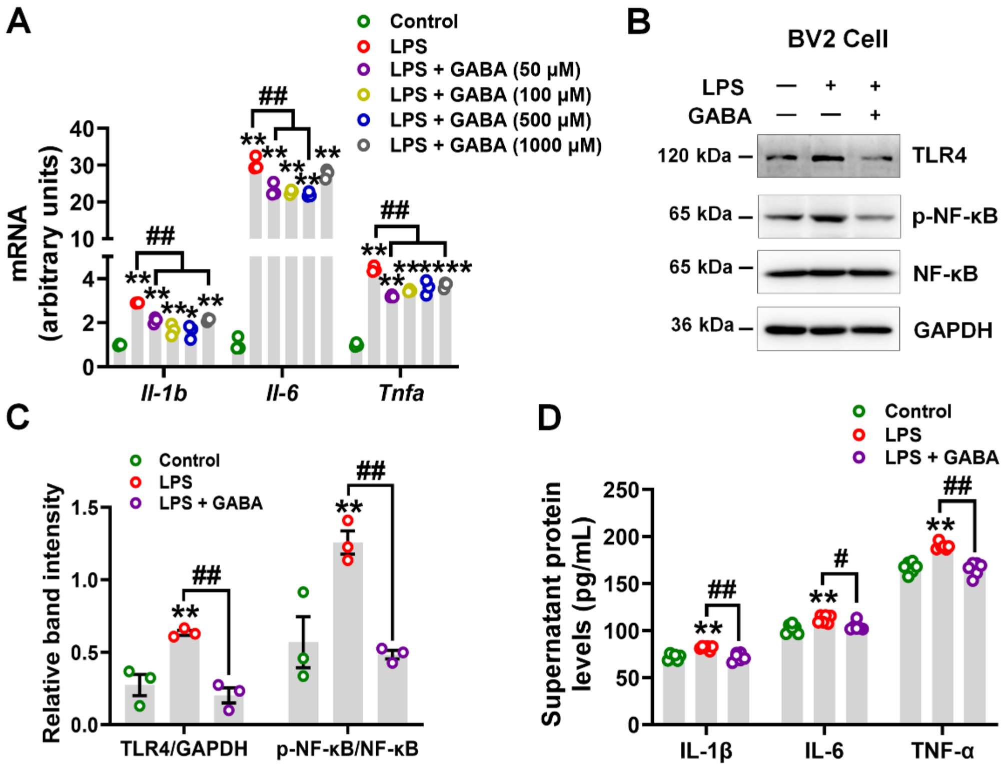

Figure S15. GABA supplementation reduces pro-inflammatory response in LPS-treated BV2 cells. (A) The mRNA expression levels of *Il-1b*, *Il-6*, and *Tnfa* in LPS-treated cells with different concentrations of GABA (50, 100, 500 and 1000 μM). n = 3 per group. (B and C) Representative blots and quantification showing TLR4, p-NF-κB, and NF-κB expression in LPS-treated cells treated without or with 50 μM GABA. n = 3 per group. (D) Cellular supernatant levels of IL-1β, IL-6 and TNF-α in LPS-treated cells without or with 50 μM GABA. n = 6 per group. Results are expressed as the mean ± SEM. $^{**}p < 0.01$, $^{*}p < 0.05$ vs. Control; $^{\#\#}p < 0.01$, $^{\#}p < 0.05$ vs. LPS. Statistical significance was determined using one-way ANOVA and Tukey's tests for *post hoc* comparisons.

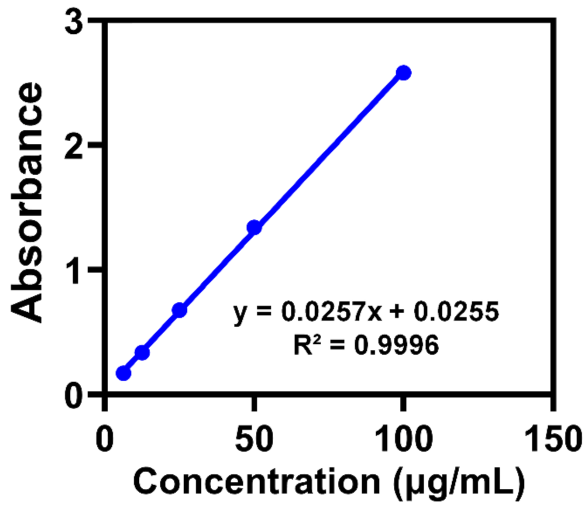

Figure S16. Characterization of PLX. UV-vis spectroscopy was used to the determine the concentration of PLX. The standard curves were linear over the range of 6.25–100 μg/mL.

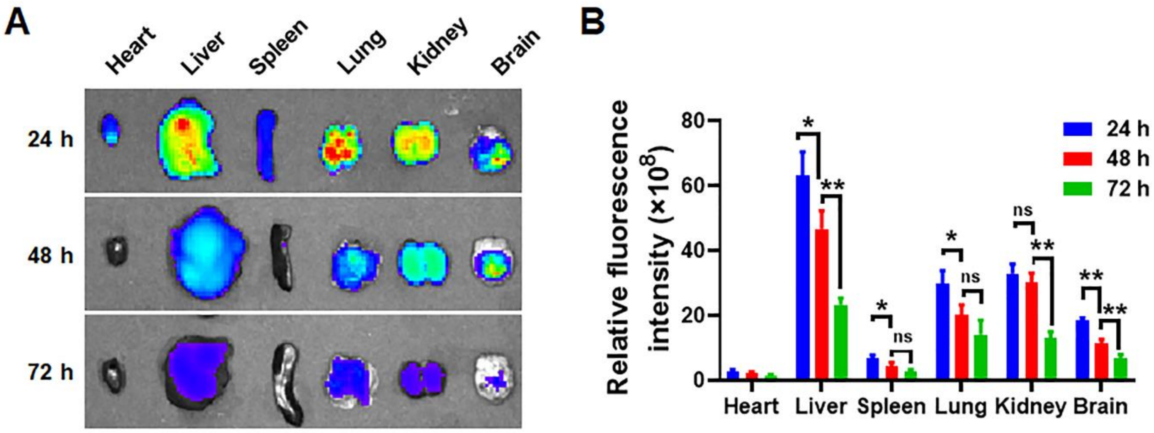

Figure S17. ***Ex vivo*** **evaluation of fluorescence signals in organs at different time points.** (A and B) *Ex vivo* images and corresponding fluorescence analysis of sacrificed tissues (heart, liver, spleen, lung, kidney, and brain) after intravenous injection of Cy5.5-labelled NPs@PLX or MNPs@PLX at 24, 48 and 72 h. n = 3 per group. Results are expressed as the mean ± SEM. $^{**}p < 0.01$, $^{*}p < 0.05$. Statistical significance was determined using one-way ANOVA and Tukey's tests for *post hoc* comparisons.

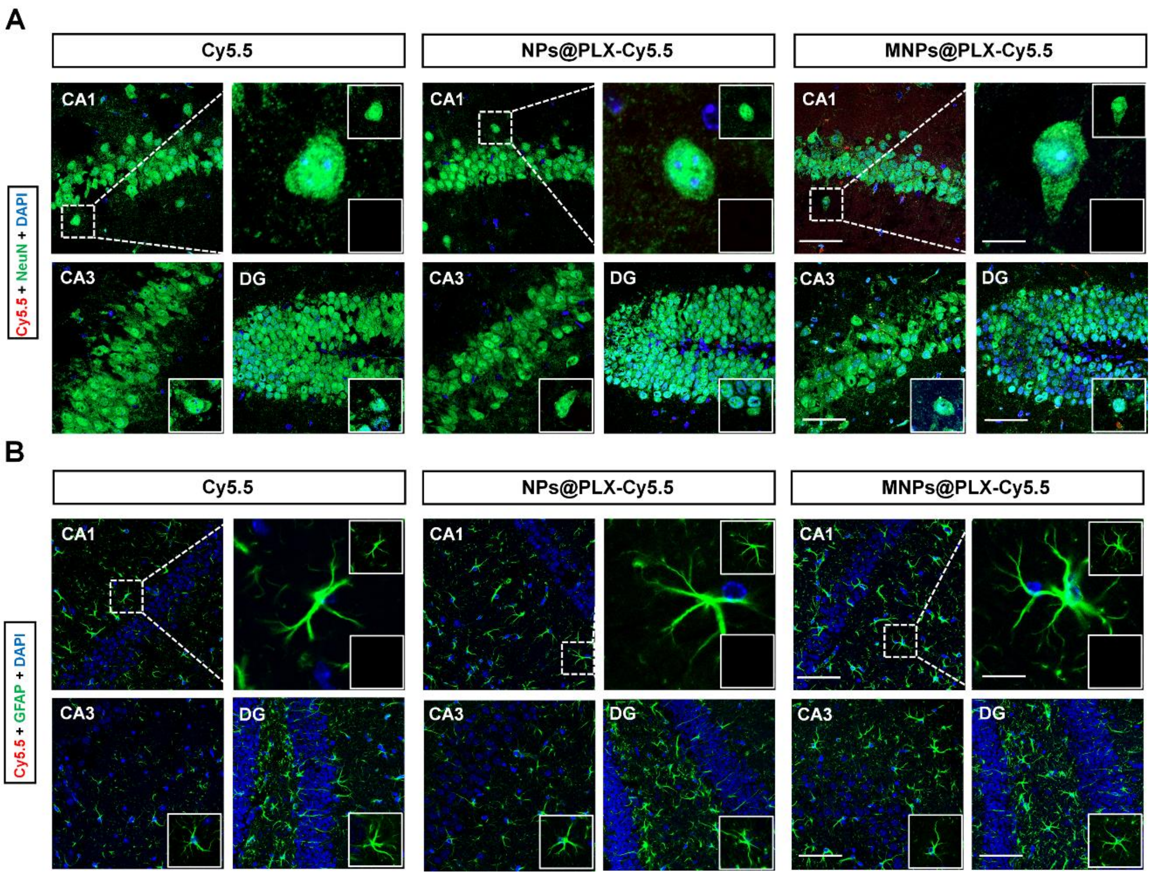

Figure S18. Distribution of MNPs@PLX in the hippocampus. Representative images of NeuN (A) and GFAP (B) staining at 6 h post-injection in the hippocampal CA1, CA3 and DG areas in mice treated with Cy5.5, NPs@PLX-Cy5.5, or MNPs@PLX-Cy5.5. Scale bars, 40 μm. Magnified images are shown in the right panels for CA1. Scale bars, 10 μm.

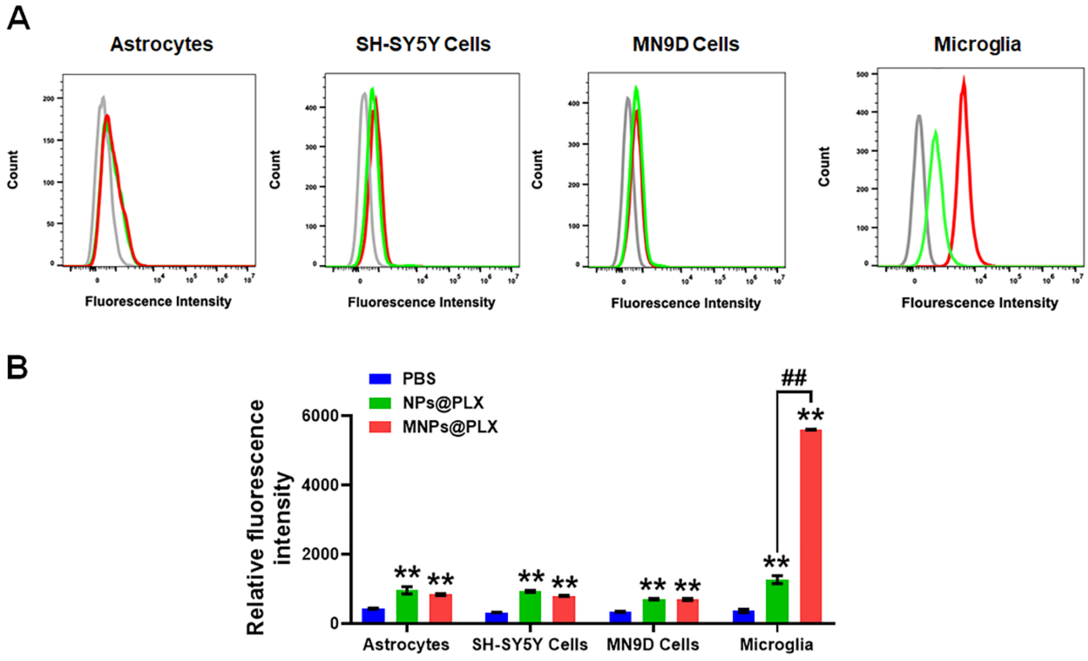

Figure S19. Cellular uptake of MNPs@PLX by different cells. (A and B) Flow cytometry analysis of cellular uptake of NPs@PLX and MNPs@PLX in astrocytes, SH-SY5Y cells, MN9D cells and microglia. n = 3 per group. Results are expressed as the mean ± SEM. $^{**}p < 0.01$ vs. PBS; $^{\#\#}p < 0.01$ vs. NPs@PLX. Statistical significance was determined using one-way ANOVA and Tukey's tests for *post hoc* comparisons.

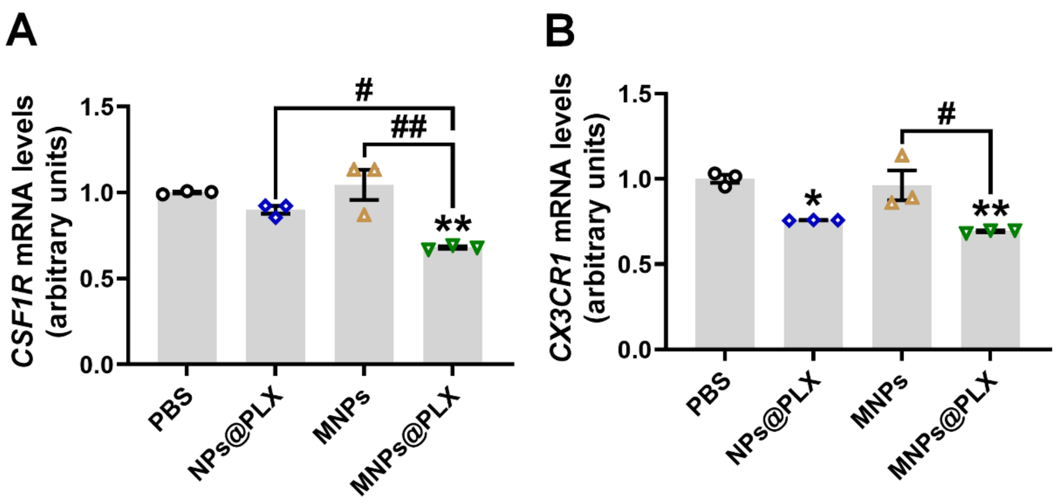

Figure S20. Effect of MNPs@PLX on microglial markers in the hippocampus. The mRNA expression levels of *Csf1r* (A) and *Cx3cr1* (B) in hippocampi of WT mice treated with NPs@PLX, MNPs or MNPs@PLX. n = 3 per group. Results are expressed as the mean ± SEM. $^{**}p < 0.01$, $^{*}p < 0.05$ vs. PBS; $^{\#\#}p < 0.01$, $^{\#}p < 0.05$ vs. MNPs@PLX. Statistical significance was determined using one-way ANOVA and Tukey's tests for *post hoc* comparisons.

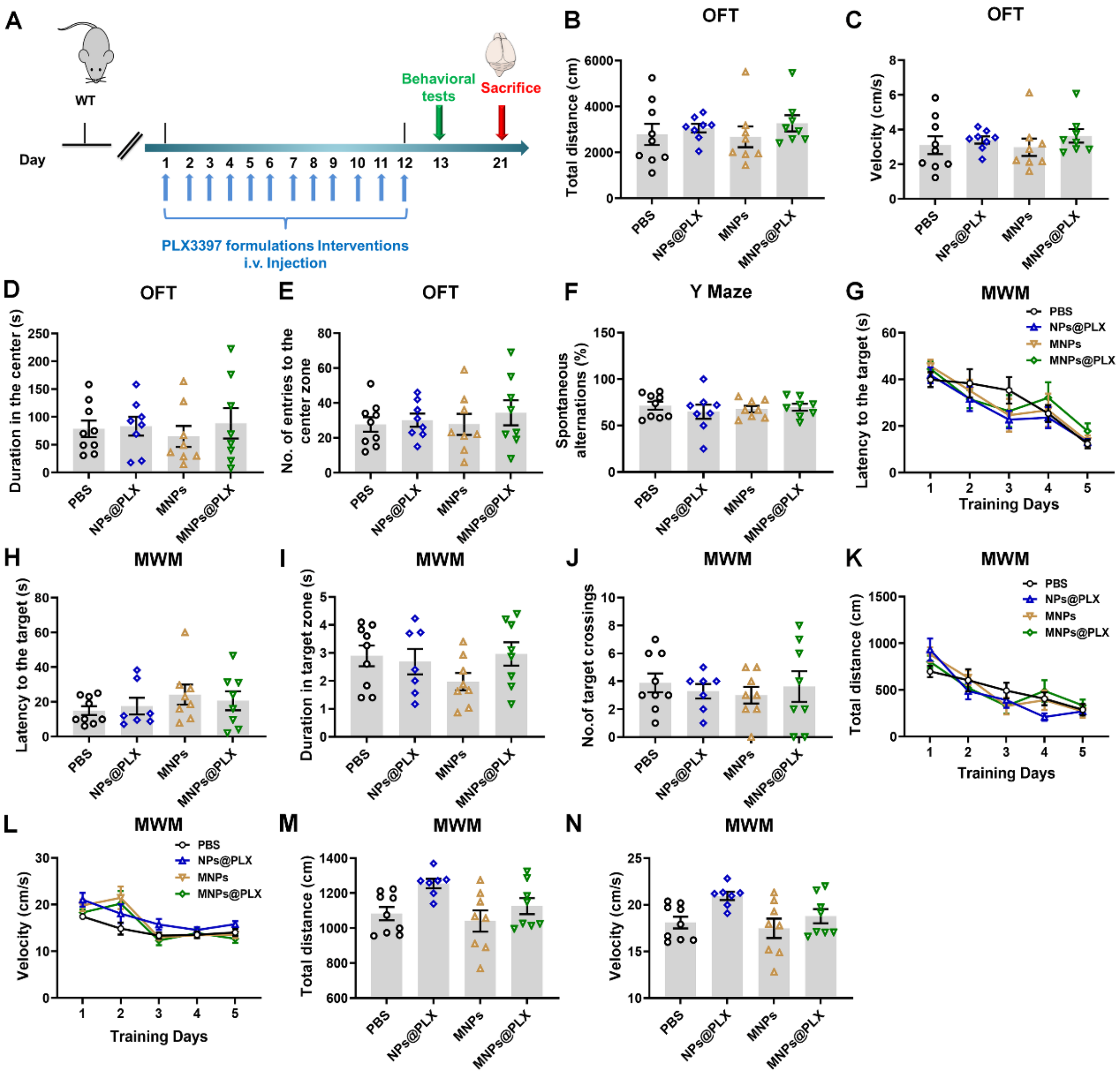
A
WT
Day
Behavioral tests
Sacrifice
1 2 3 4 5 6 7 8 9 10 11 12 13 21
PLX3397 formulations Interventions
i.v. Injection
B OFT
Total distance (cm)
C OFT
Velocity (cm/s)
D OFT
Duration in the center (s)
E OFT
No. of entries to the center zone
F Y Maze
Spontaneous alternations (%)
G MWM
Latency to the target (s)
Training Days
H MWM
Latency to the target (s)
I MWM
Duration in target zone (s)
J MWM
No.of target crossings
K MWM
Total distance (cm)
Training Days
L MWM
Velocity (cm/s)
Training Days
M MWM
Total distance (cm)
N MWM
Velocity (cm/s)
PBS
NPs@PLX
MNPs
MNPs@PLX

Figure S21. Behavioral performance of WT mice treated with MNPs@PLX. (A) Experimental design for NPs@PLX, MNPs and MNPs@PLX administration in WT mice. The total travelled distance (B), movement speed (C), time spent in the center zone of the open field (D), and number of entries to the center zone (E) of WT mice treated with NPs@PLX, MNPs or MNPs@PLX. (F) Spontaneous alterations in the Y maze for WT mice treated with NPs@PLX, MNPs or MNPs@PLX. (G) Escape latency over a five-day training course. In the probe tests, mice were analyzed for the (H) escape latency, (I) time spent in the target zone, and (J) number of target crossing. Total swimming distance (K) and swimming speed (L) of WT mice treated with NPs@PLX, MNPs or MNPs@PLX over a five-day training course. Total swimming distance (M) and swimming speed (N) of WT mice treated with NPs@PLX, MNPs or MNPs@PLX in probe tests. Results are expressed as the mean ± SEM. Statistical significance was determined using one-way ANOVA and Tukey's tests for *post hoc* comparisons.

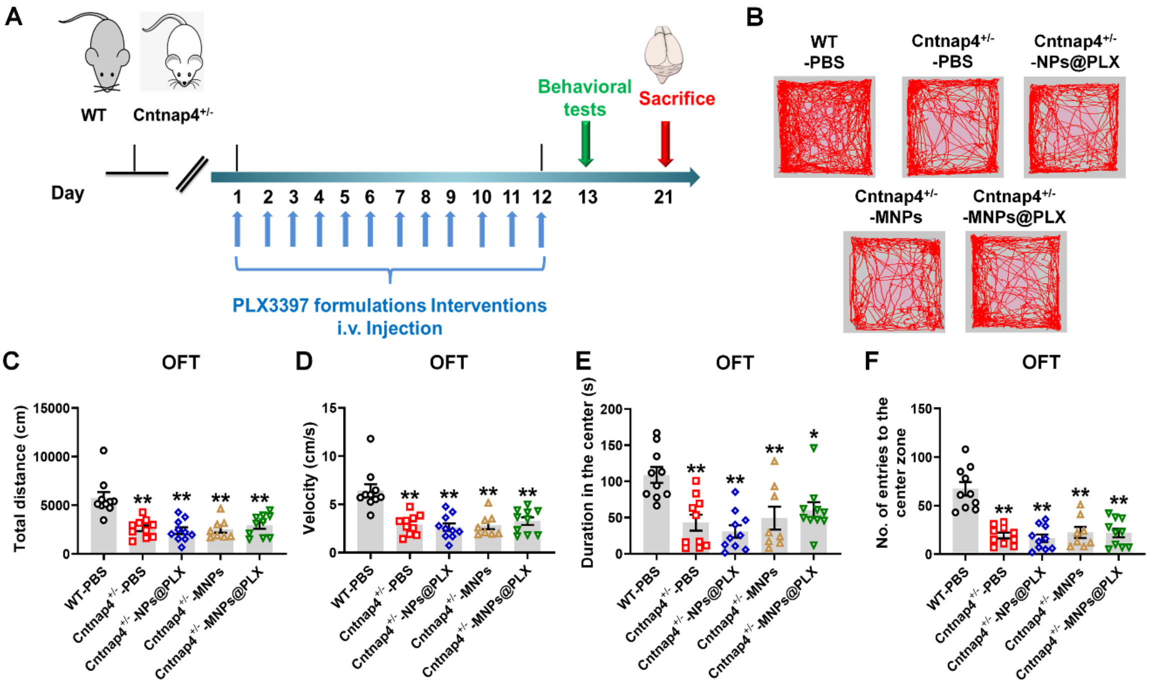

Figure S22. Behavioral tests of MNPs@PLX administration in Cntnap4$^{+/-}$ mice. (A) Experimental design for NPs@PLX, MNPs and MNPs@PLX administration in Cntnap4$^{+/-}$ mice. (B) Representative traces in the open field. (C-F) Total travelled distance, movement speed, time spent in the center zone, and number of entries to the center zone in the open field. n = 8–10. Results are expressed as the mean ± SEM. $^{**}p < 0.01$, $^{*}p < 0.05$ vs. WT-PBS. Statistical significance was determined using one-way ANOVA and Tukey's tests for *post hoc* comparisons.

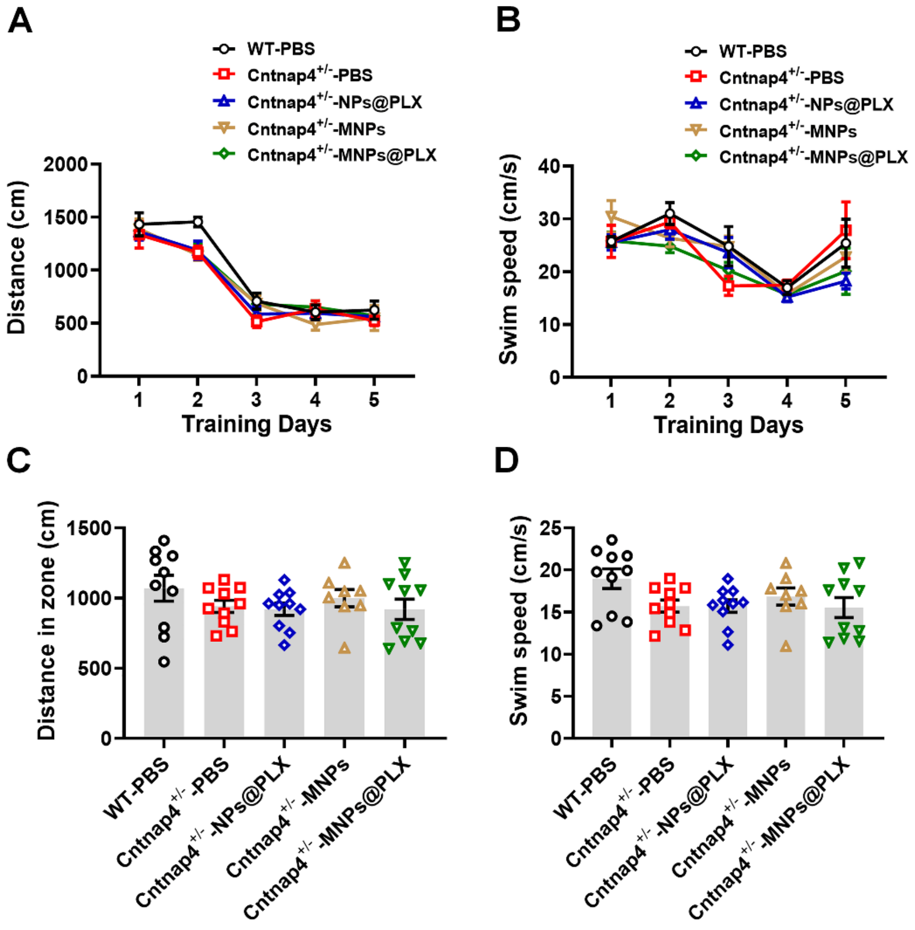

Figure S23. Swimming speed after MNPs@PLX administration in Cntnap4$^{+/-}$ mice in a water maze. Total swimming distance (A) and swimming speed (B) of Cntnap4$^{+/-}$ mice treated with NPs@PLX, MNPs or MNPs@PLX over a five-day training course. Total swimming distance (C) and swimming speed (D) of Cntnap4$^{+/-}$ mice treated with NPs@PLX, MNPs or MNPs@PLX in the probe tests. Results are expressed as the mean ± SEM. Statistical significance was determined using one-way ANOVA and Tukey's tests for *post hoc* comparisons.

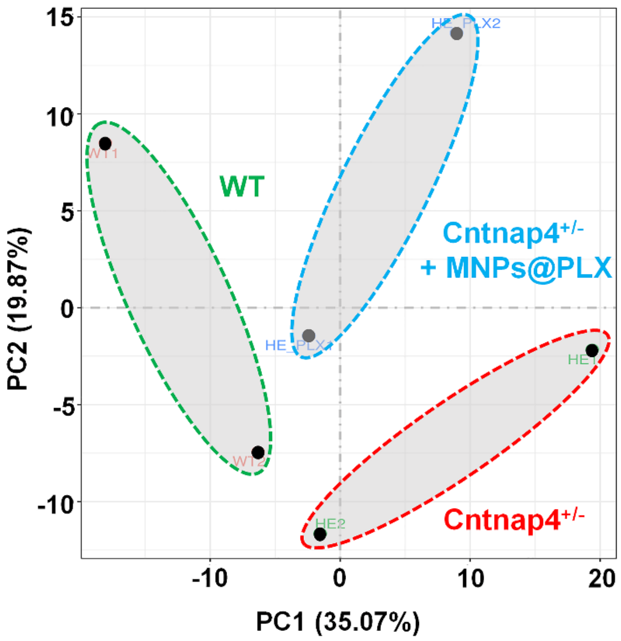

Figure S24. Hippocampal RNA-seq profile of MNPs@PLX supplementation in Cntnap4$^{+/-}$ mice. PCA plot of RNAs from WT, Cntnap4$^{+/-}$ and Cntnap4$^{+/-}$ + MNPs@PLX mice.

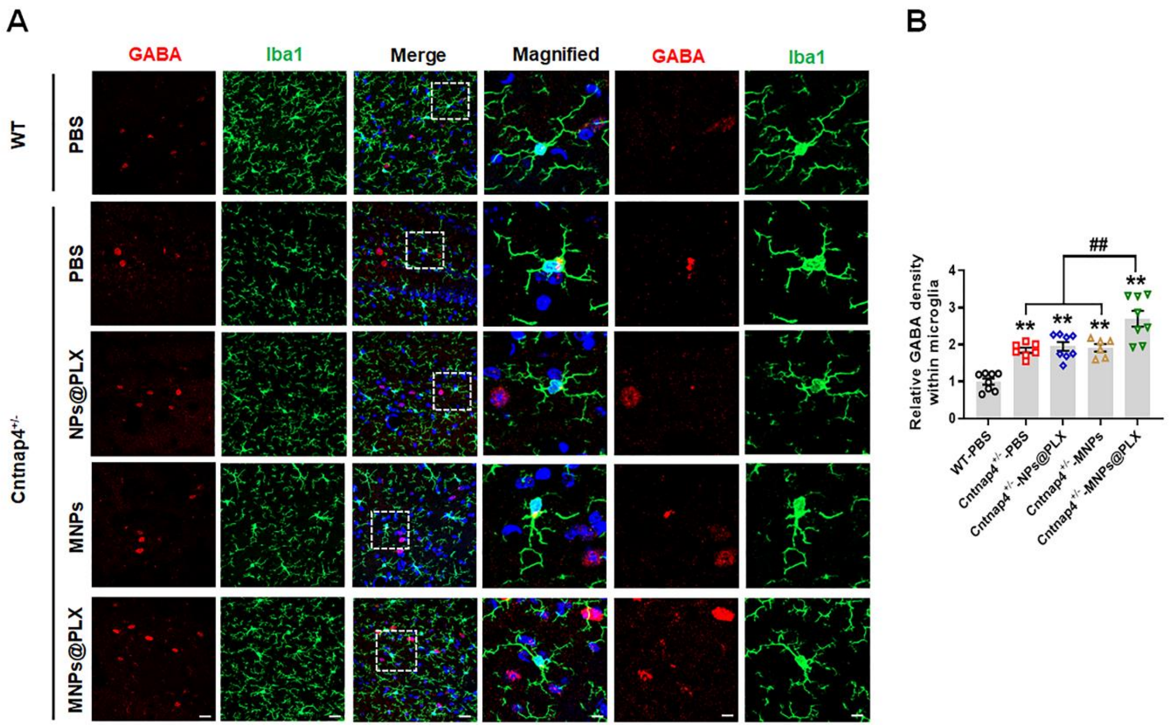

Figure S25. Effect of MNPs@PLX on the GABA in Cntnap4$^{+/-}$ mice. (A and B) Immunofluorescence staining and quantification of Iba1 colocalization with GABA in hippocampi of Cntnap4$^{+/-}$ mice treated with NPs@PLX, MNPs or MNPs@PLX. n = 6–8. Scale bars, 20 μm. Magnified images are shown in the right column. Scale bars, 5 μm. Results are expressed as the mean ± SEM. $^{**}p < 0.01$ vs. WT; $^{\#\#}p < 0.01$ vs. Cntnap4$^{+/-}$ + MNPs@PLX. Statistical significance was determined using one-way ANOVA and Tukey's tests for *post hoc* comparisons.

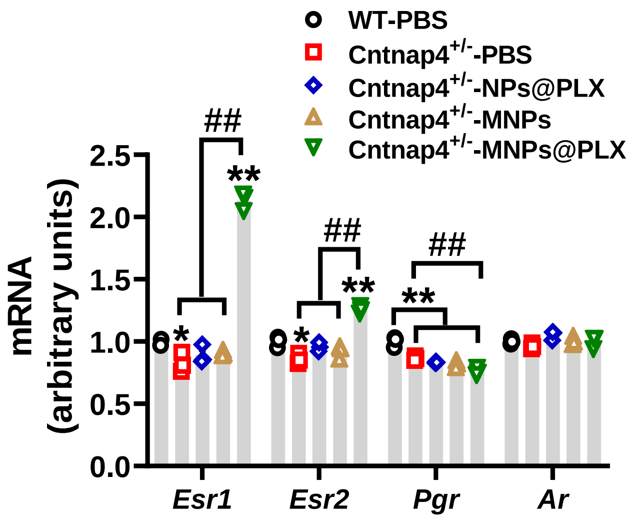

Figure S26. Effect of MNPs@PLX on the hormone receptors in Cntnap4$^{+/-}$ mice. The mRNA expression levels of *Esr1*, *Esr2*, *Pgr* and *Ar* in hippocampi of Cntnap4$^{+/-}$ mice treated with NPs@PLX, MNPs or MNPs@PLX. n = 3 per group. Results are expressed as the mean ± SEM. $^{**}p < 0.01$, $^{*}p < 0.05$ vs. WT-PBS; $^{\#\#}p < 0.01$ vs. Cntnap4$^{+/-}$ + MNPs@PLX. Statistical significance was determined using one-way ANOVA and Tukey's tests for *post hoc* comparison.

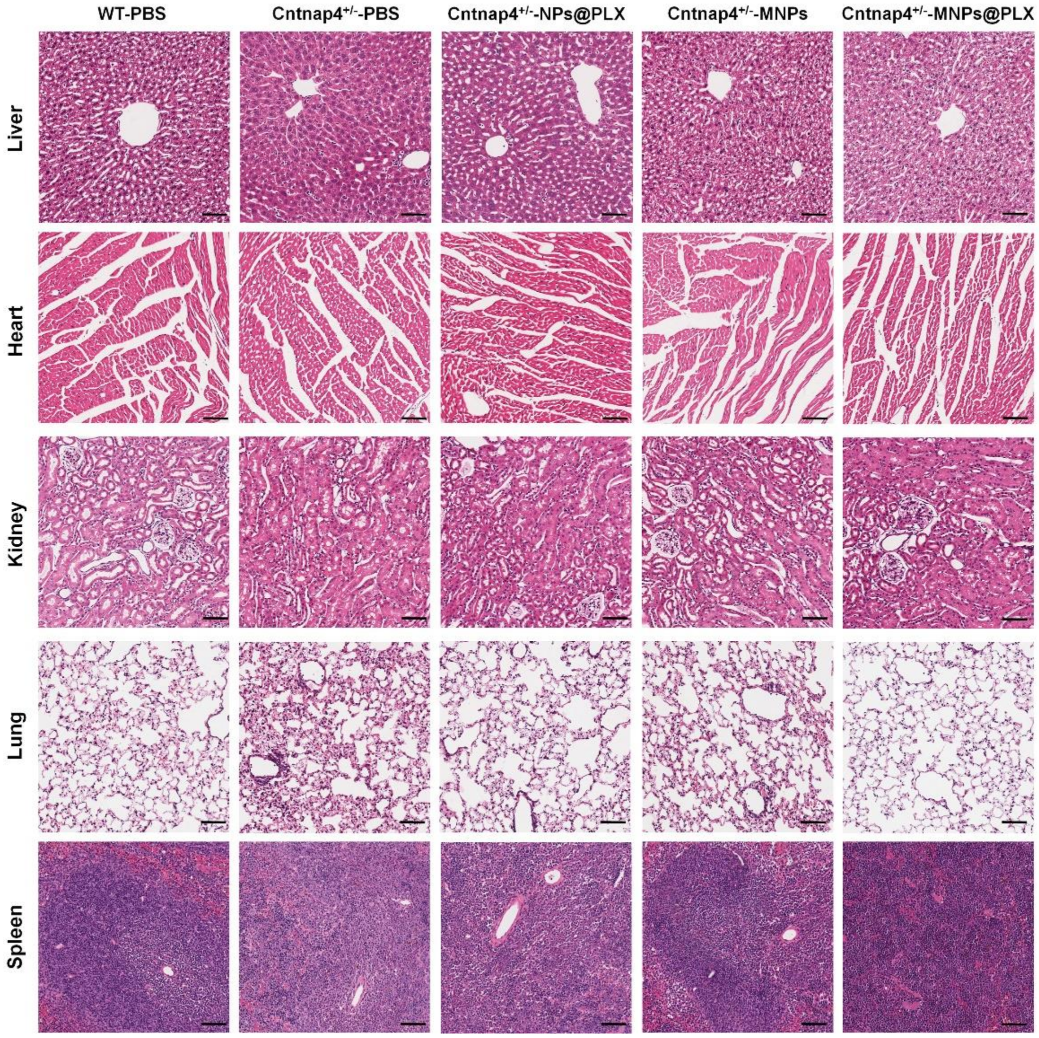

Figure S27. MNPs@PLX exerts no observable toxicity to major organs. Representative images of hematoxylin and eosin (HE) staining of major organs (including liver, heart, kidney, lung and spleen) excised from WT-PBS, Cntnap4$^{+/-}$-PBS, Cntnap4$^{+/-}$-NPs@PLX, Cntnap4$^{+/-}$-MNPs and Cntnap4$^{+/-}$-MNPs@PLX mice, n = 3 per group. No detectable pathological changes in the major organs were observed. Scale bars, 100 µm.

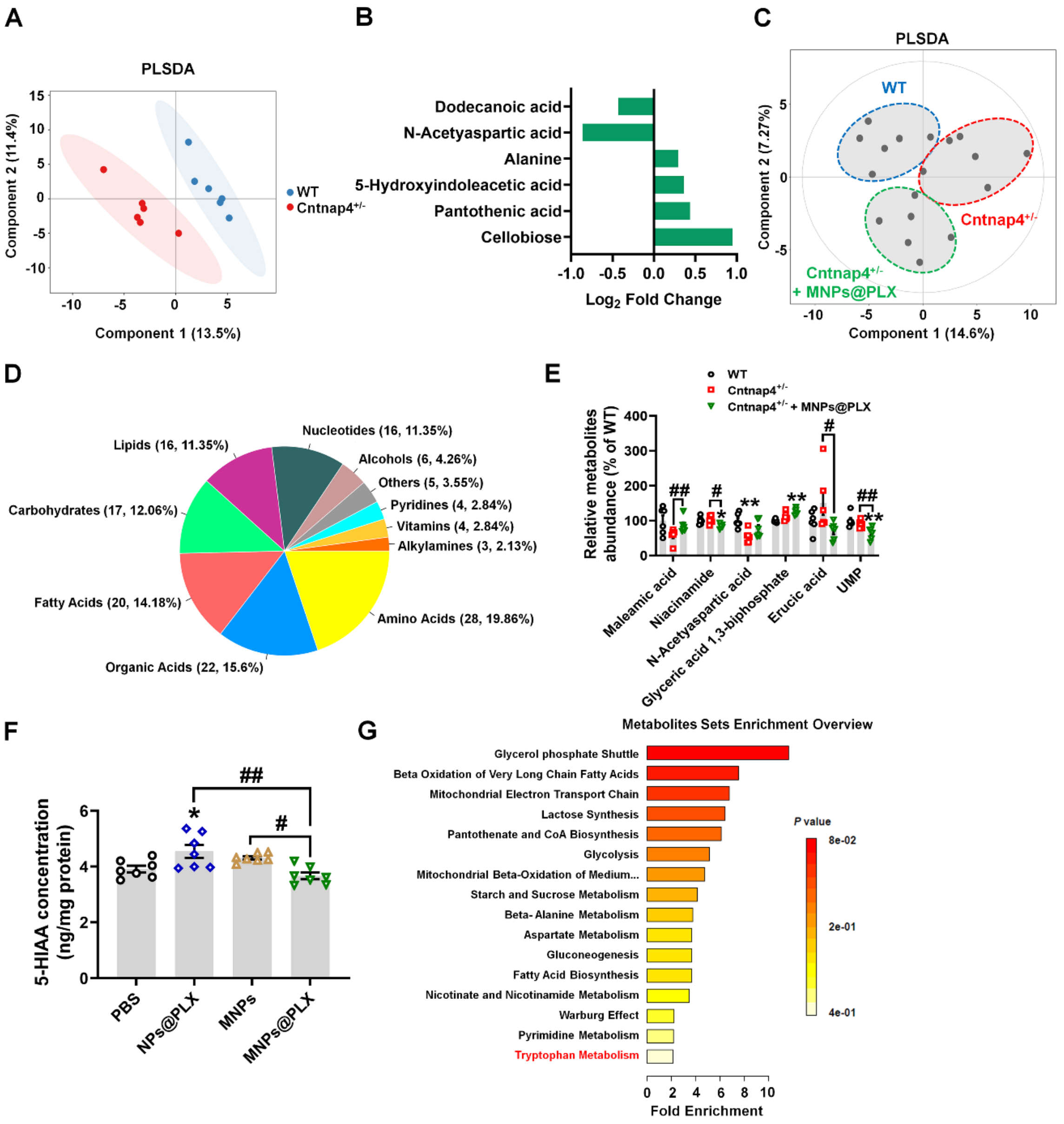
A
PLSDA
Component 2 (11.4%)
Component 1 (13.5%)
WT
Cntnap4+/-
B
Dodecanoic acid
N-Acetyaspartic acid
Alanine
5-Hydroxyindoleacetic acid
Pantothenic acid
Cellobiose
Log2 Fold Change
C
PLSDA
WT
Cntnap4+/-
Cntnap4+/- + MNPs@PLX
Component 2 (7.27%)
Component 1 (14.6%)
D
Nucleotides (16, 11.35%)
Lipids (16, 11.35%)
Alcohols (6, 4.26%)
Others (5, 3.55%)
Pyridines (4, 2.84%)
Carbohydrates (17, 12.06%)
Vitamins (4, 2.84%)
Alkylamines (3, 2.13%)
Fatty Acids (20, 14.18%)
Amino Acids (28, 19.86%)
Organic Acids (22, 15.6%)
E
WT
Cntnap4+/-
Cntnap4+/- + MNPs@PLX
Relative metabolites abundance (% of WT)
Maleamic acid
Niacinamide
N-Acetyaspartic acid
Glyceric acid 1,3-biphosphate
Erucic acid
UMP
F
5-HIAA concentration (ng/mg protein)
PBS
NPs@PLX
MNPs
MNPs@PLX
G
Metabolites Sets Enrichment Overview
Glycerol phosphate Shuttle
Beta Oxidation of Very Long Chain Fatty Acids
Mitochondrial Electron Transport Chain
Lactose Synthesis
Pantothenate and CoA Biosynthesis
Glycolysis
Mitochondrial Beta-Oxidation of Medium...
Starch and Sucrose Metabolism
Beta- Alanine Metabolism
Aspartate Metabolism
Gluconeogenesis
Fatty Acid Biosynthesis
Nicotinate and Nicotinamide Metabolism
Warburg Effect
Pyrimidine Metabolism
Tryptophan Metabolism
P value
8e-02
2e-01
4e-01
Fold Enrichment

Figure S28. Hippocampal metabonomic analysis of MNPs@PLX administration in Cntnap4$^{+/-}$ mice. (A) PLSDA plot of metabolites in WT and Cntnap4$^{+/-}$ mice. (B) The differential metabolites between WT and Cntnap4$^{+/-}$ mice. (C) PLSDA plot of metabolites in WT, Cntnap4$^{+/-}$ and Cntnap4$^{+/-}$ + MNPs@PLX mice. (D) Classification of the metabolites among WT, Cntnap4$^{+/-}$ and Cntnap4$^{+/-}$ + MNPs@PLX mice. (E) Relative levels of Maleamic acid, Niacinamide, N-Acetyaspartic acid, Glyceric acid 1,3-biphosphate, Erucic acid, and UMP in hippocampi of Cntnap4$^{+/-}$ mice treated with NPs@PLX, MNPs or MNPs@PLX. (F) The levels of 5-HIAA in hippocampi of WT mice treated with NPs@PLX, MNPs or MNPs@PLX. (G) Signaling pathways enriched by the differential metabolites among WT, Cntnap4$^{+/-}$ and Cntnap4$^{+/-}$ + MNPs@PLX groups. Results are expressed as the mean ± SEM. $^{**}p < 0.01$, $^{*}p < 0.05$ vs. WT or PBS; $^{\#\#}p < 0.01$, $^{\#}p < 0.05$ vs. Cntnap4$^{+/-}$ + MNPs@PLX or MNPs@PLX. Statistical significance was determined using one-way ANOVA and Tukey's tests for *post hoc* comparisons.

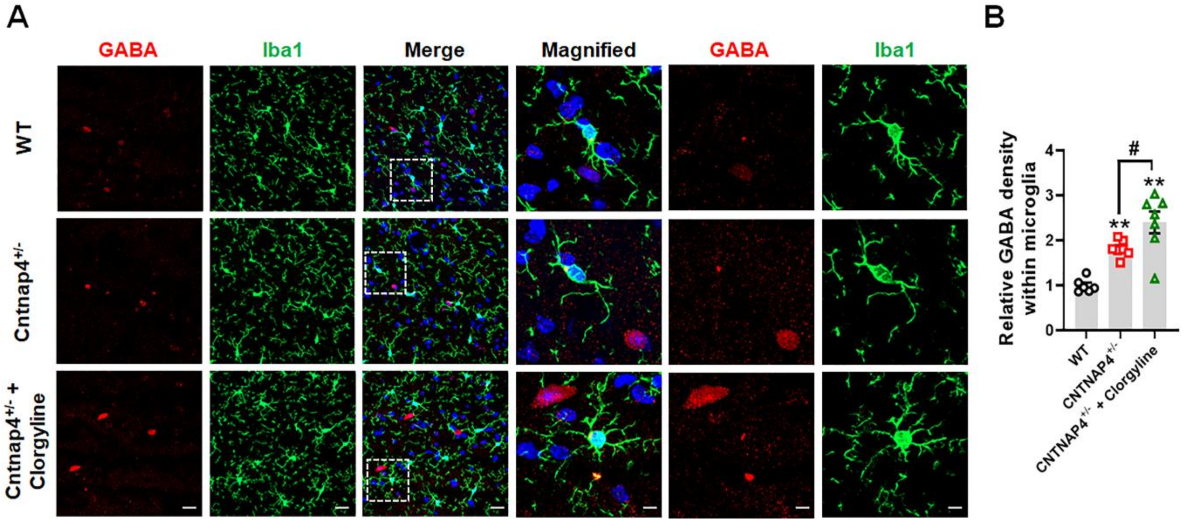

Figure S29. Effect of MAOA inhibitor on the GABA in Cntnap4$^{+/-}$ mice. (A and B) Immunofluorescence staining and quantification of Iba1 colocalization with GABA in hippocampi of WT and Cntnap4$^{+/-}$ mice treated with clorgyline. n = 6–7. Scale bars, 20 μm. Magnified images are shown in the right column. Scale bars, 5 μm. Results are expressed as the mean ± SEM. $^{**}p < 0.01$ vs. WT; $^{\#}p < 0.05$ vs. Cntnap4$^{+/-}$. Statistical significance was determined using one-way ANOVA and Tukey's tests for *post hoc* comparisons.

Table S1. Analysis of liver and kidney function following NPs@PLX, MNPs and MNPs@PLX administration in Cntnap4$^{+/-}$ mice.

	WT (n=4)	Cntnap4$^{+/-}$ (n=4)	Cntnap4$^{+/-}$ + NPs@PLX (n=4)	Cntnap4$^{+/-}$ + MNPs (n=4)	Cntnap4$^{+/-}$ + MNPs@PLX (n=4)	*P* Value
**AST**	98.50 (2.40)	104.25 (3.86)	114.75 (4.50)	124.75 (12.21)	120.50 (9.87)	0.131
**ALT**	44.25 (3.33)	58.50 (4.87)	63.75 (6.13)	58.25 (8.75)	55.50 (3.97)	0.228
**AST/ALT**	2.28 (0.23)	1.81 (0.11)	1.85 (0.19)	2.23 (0.27)	2.20 (0.20)	0.377
**TP**	54.20 (4.13)	65.10 (2.96)	61.98 (3.23)	64.68 (1.51)	67.13 (2.08)	0.054
**ALB**	18.93 (1.43)	20.28 (0.86)	18.08 (1.09)	18.08 (1.01)	20.00 (0.45)	0.459
**GLOB**	37.78 (3.08)	44.83 (3.49)	43.90 (2.48)	46.40 (2.16)	47.13 (2.47)	0.185
**A/G**	0.50 (0.06)	0.45 (0.06)	0.40 (0.04)	0.40 (0.04)	0.43 (0.05)	0.625
**ALP**	100.25 (4.37)	105.75 (11.23)	90.50 (15.92)	99.00 (7.38)	120.25 (5.15)	0.327
**GLU**	5.62 (0.83)	6.38 (0.37)	5.18 (0.44)	4.17 (1.28)	6.68 (0.56)	0.208
**UREA**	7.40 (0.63)	8.98 (0.39)	8.65 (0.33)	8.40 (0.40)	8.95 (0.77)	0.258
**CR**	12.75 (0.48)	12.75 (0.25)	13.25 (0.48)	12.50 (0.29)	13.75 (1.31)	0.712
**UA**	337.00 (25.39)	326.50 (19.26)	367.50 (37.80)	344.50 (30.50)	324.25 (21.99)	0.810
**LDH**	751.25 (167.41)	702.50 (44.02)	819.00 (131.73)	833.00 (79.16)	800.00 (94.70)	0.917

AST: aspartate aminotransferase; ALT: alanine aminotransferase; AST/ALT: aspartate aminotransferase alanine aminotransferase ratio; TP: Total protein; ALB: albumin; GLOB: globulin; A/G: albumin globulin ratio; ALP: alkaline phosphatase; GLU: blood glucose: UREA: urea nitrogen; CR: creatinine; UA: uric acid; LDH: lactate dehydrogenase. Results are expressed as the mean ± SEM. n = 4 per group. Statistical significance was determined by one-way ANOVA and Tukey tests for *post-hoc* comparisons.

Table S2. Primer sequences used for qRT-PCR.

Mice genes	Primer sequence (5′-3′)
Il-1b	F: AATGCCACCTTTTGACAGTGAT
	R: TGCTGCGAGATTTGAAGCTG
Il-6	F: AGGATACCACTCCCAACAGACC
	R: AAGTGCATCATCGTTCATACA
Tnfa	F: CACGTCGTAGCAAACCACC
	R: TGAGATCCATGCCGTTGGC
Ifng	F: TGGCAGGAGATGTCTACACT
	R: GAAGCACCAGGTGTCAAGTC
Tgfb	F: ATTCCTGGCGTTACCTTGG
	R: AGCCCTGTATTCCGTCTCCT
Csf1r	F: CCTCAAACGTGGAGACACCAA
	R: CGTGTGCCAACATCATTGCT
Cx3cr1	F: CAACCCCTTTATCTACGCCTT
	R: GACCCATCTCCCTCGCTTG
Tmem119	F: CTGACATTCTGGCTGCTACC
	R: CACCCTTCACAGGCTTTGCTC
P2ry12	F: TTTGCTGGGCTCATCACGAAC
	R: ACTGAAGTAACTTGGCACACC
Maoa	F: CAAGAGCCTGAGTCCAAGGATGTTC
	R: ACAAAGCAGAGAAGAGCCACAGAAG
Esr1	F: CTACTACCTGGAGAACGAGC
	R: GCGTCGATTGTCAGAATTAGAC
Esr2	F: CTTCGCAAGTGTTACGAAGTAG

	R: GCACTTCTCTGTCTTCGTACTA
Pgr	F: TAGTCTCGCCTATACCGATCTC
	R: CTTCCCTATGAGTGGCTTCTAC
Ar	F: TAAAGACATTTTGAACGAGGCC
	R: GTCAGATATGGTTGAATTGCCC
Gapdh	F: ACGGGAAGCTCACTGGCATGGCCTT
	R: CATGAGGTCCACCACCCTGTTGCTG

The primer sequences of *Il-1b, IL-6, Tnfa*, *Ifng*, *Tgfb, Csf1r*, *Cx3cr1*, *Tmem119, P2ry12*, *Maoa*, *Esr1*, *Esr2, Pgr*, *Ar*, *Gapdh*.
