# Supplementary material for: Microglial targeted therapy relieves cognitive impairment caused by Cntnap4 deficiency
Source: Exploration (Beijing). 2023 May 10;3(3):20220160. doi: 10.1002/EXP.20220160 (PMC10624376; doi:10.1002/EXP.20220160)

**Figure 2I**

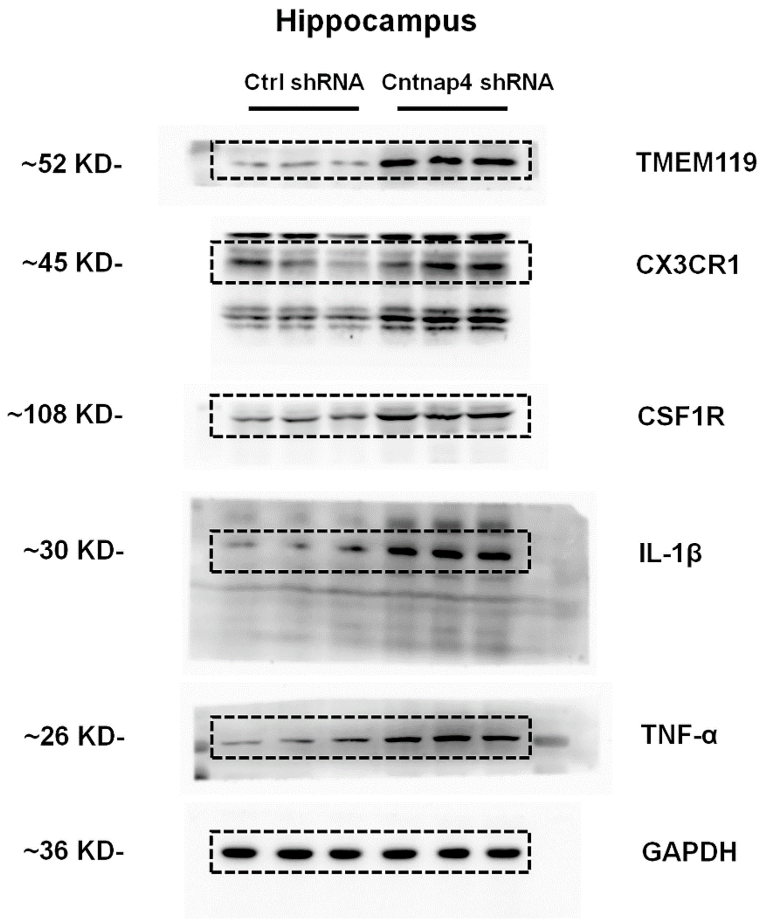


**Figure 3B**

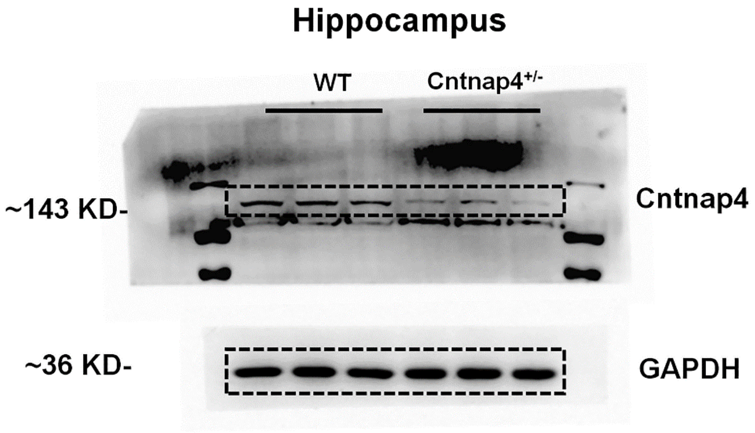

**Figure 3C**

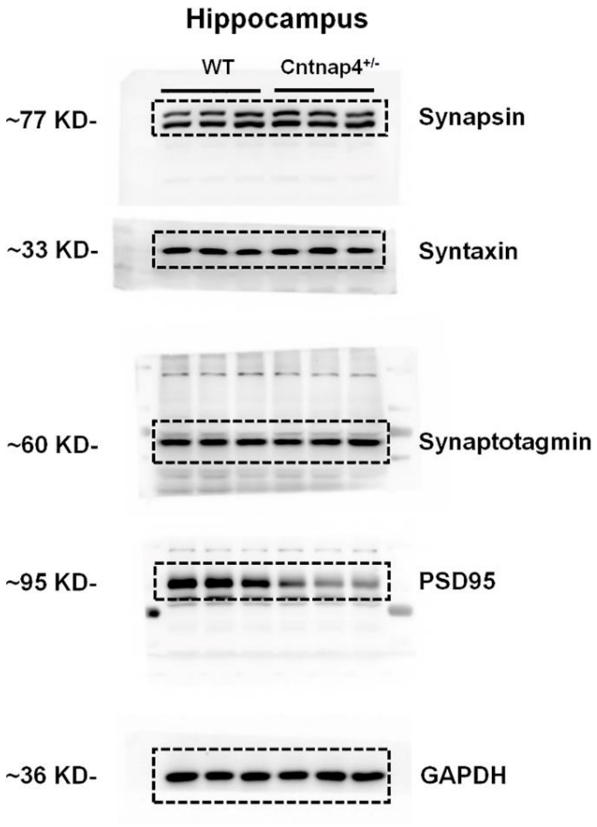


**Figure 3S**

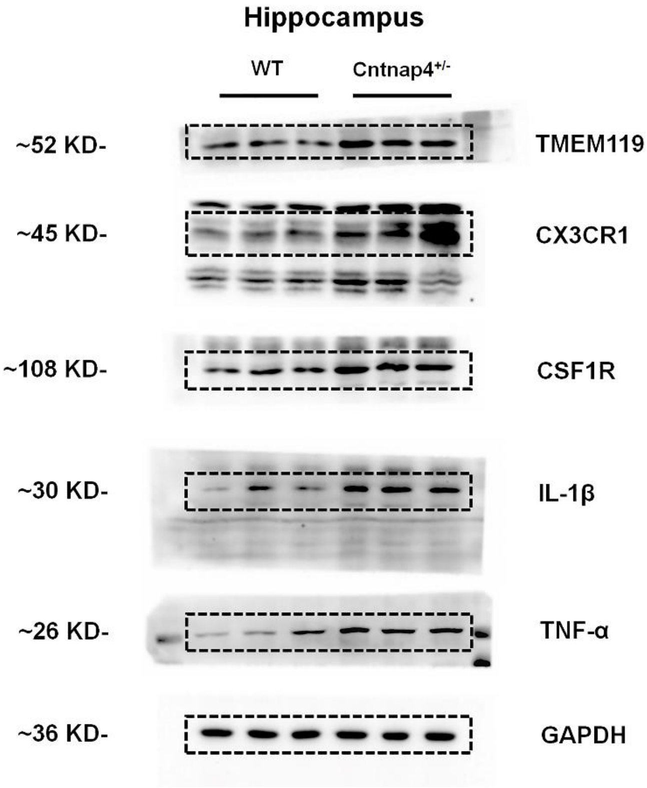

Figure 4B

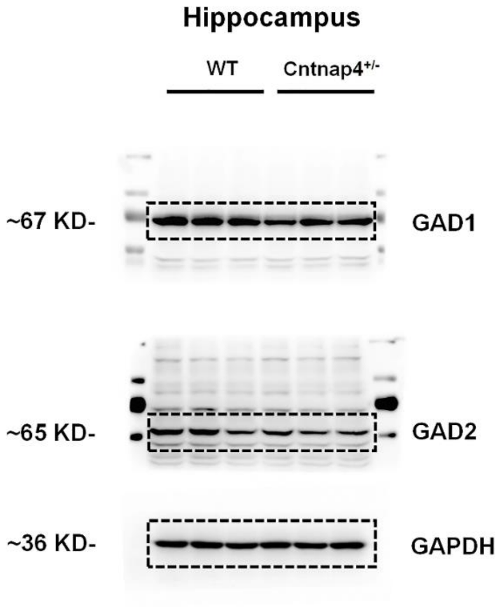


Figure 4V

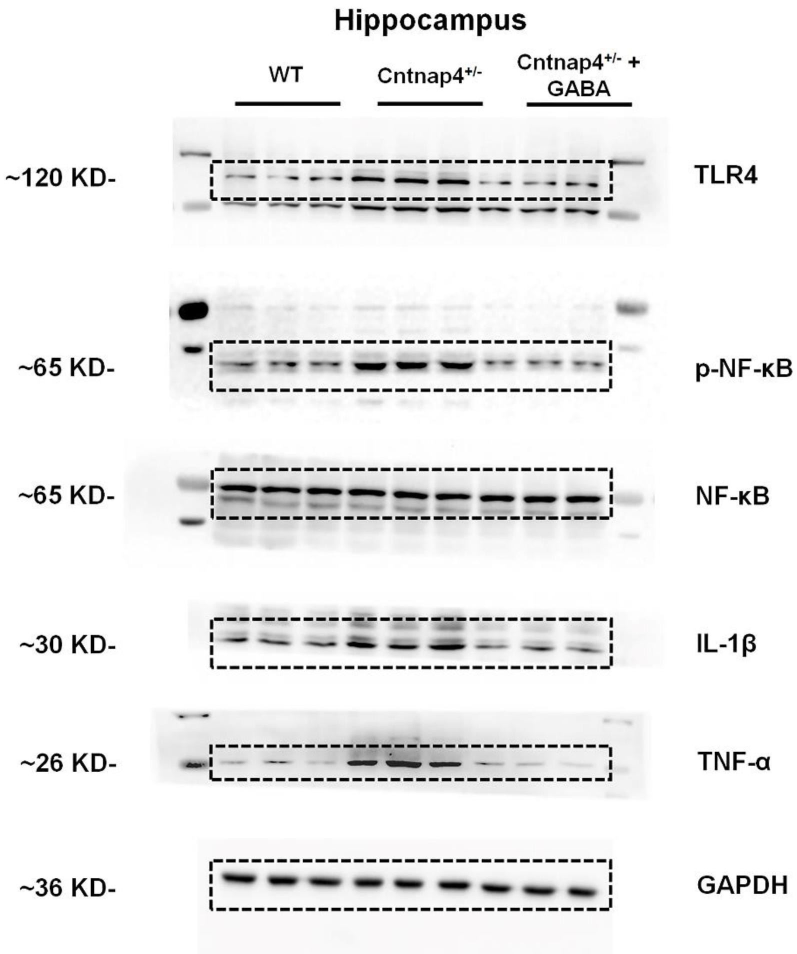

## Figure 8J

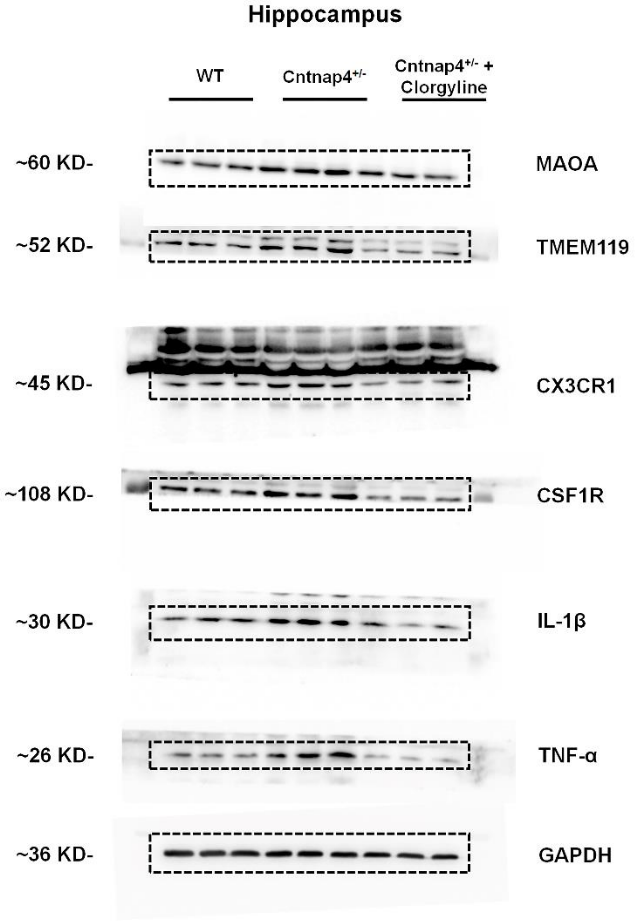


## Figure 8O

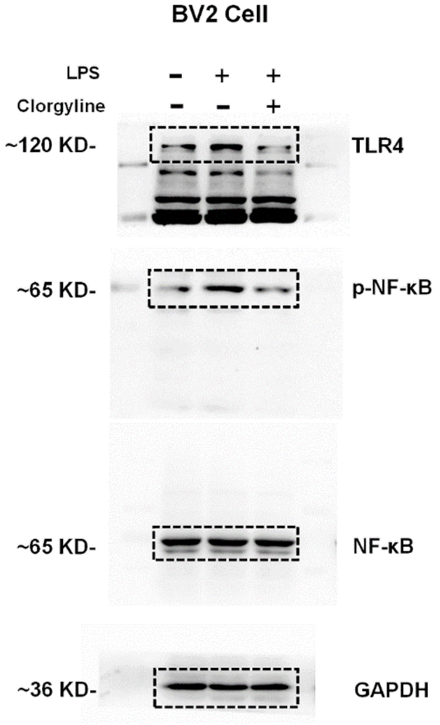

Figure S1A

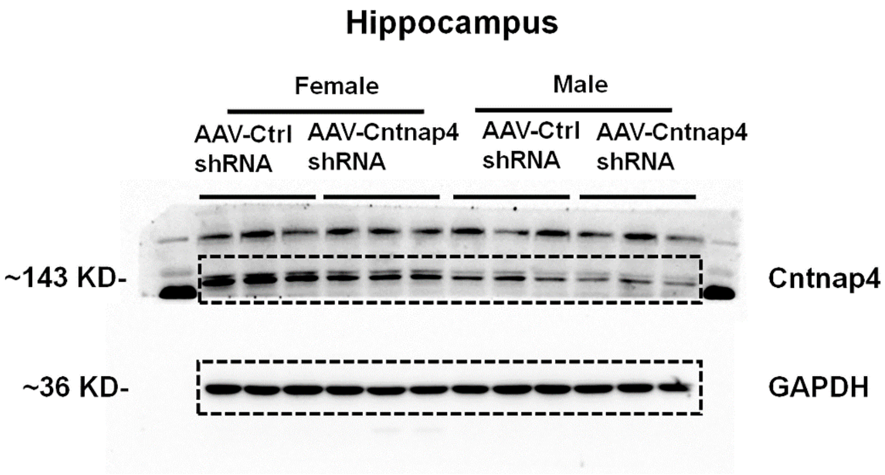


Figure S12A

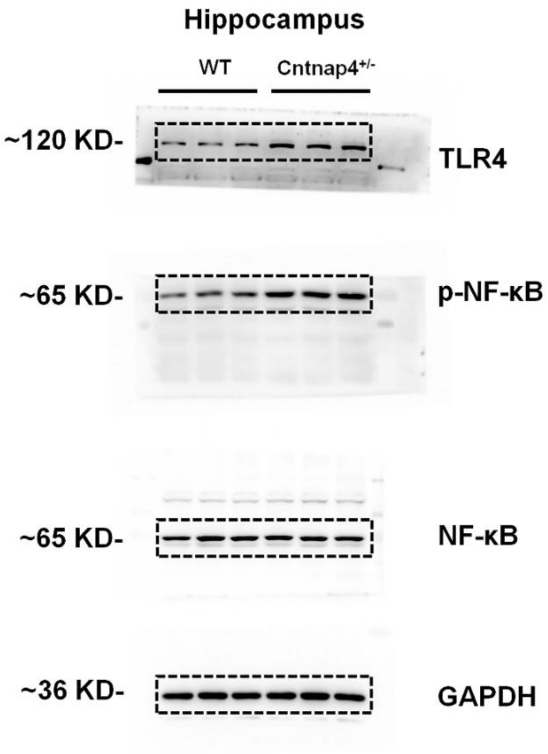

**Figure S15B**

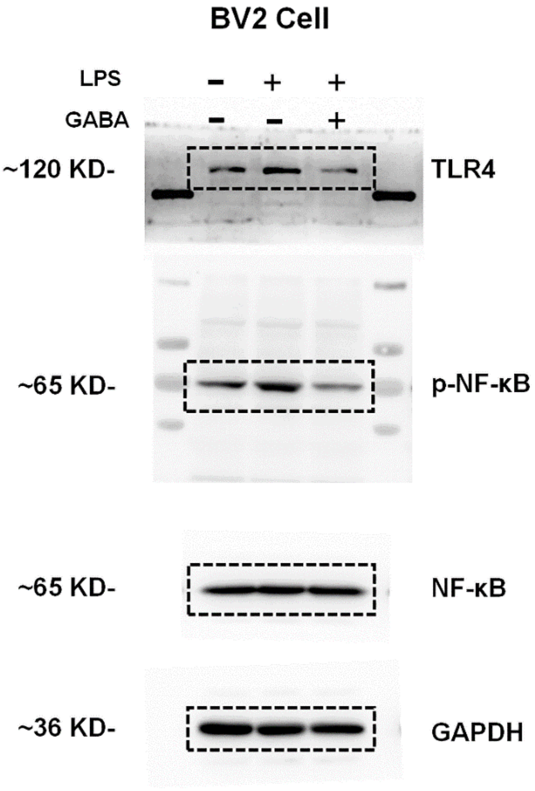

Supplement: Supplementary file 2 — Supporting Information [file EXP2-3-20220160-s002.pdf]
